# Interactome and evolutionary conservation of Dictyostelid small GTPases and their direct regulators

**DOI:** 10.1080/21541248.2021.1984829

**Published:** 2021-10-05

**Authors:** Gillian Forbes, Christina Schilde, Hajara Lawal, Koryu Kin, Qingyou Du, Zhi-hui Chen, Francisco Rivero, Pauline Schaap

**Affiliations:** a School of Life Sciences, University of Dundee, Dundee, UK; b CSIC-Universitat Pompeu Fabra, Institut de Biologia Evolutiva (Csic-universitat Pompeu Fabra), Barcelona, Spain; c Centre for Atherothrombosis and Metabolic Disease, Hull York Medical School, Faculty of Health Sciences, University of Hull, Hull, UK

**Keywords:** GTP binding proteins, guanine nucleotide exchange factors, GTPase activating proteins, stage- and cell-type specific transcriptomics, hierarchical clustering, interactome, vesicle trafficking, cell signalling, cytokinesis, cell motility

## Abstract

GTP binding proteins known as small GTPases make up one of the largest groups of regulatory proteins and control almost all functions of living cells. Their activity is under, respectively, positive and negative regulation by guanine nucleotide exchange factors (GEFs) and GTPase activating proteins (GAPs), which together with their upstream regulators and the downstream targets of the small GTPases form formidable signalling networks. While genomics has revealed the large size of the GTPase, GEF and GAP repertoires, only a small fraction of their interactions and functions have yet been experimentally explored. Dictyostelid social amoebas have been particularly useful in unravelling the roles of many proteins in the Rac-Rho and Ras-Rap families of GTPases in directional cell migration and regulation of the actin cytoskeleton. Genomes and cell-type specific and developmental transcriptomes are available for *Dictyostelium* species that span the 0.5 billion years of evolution of the group from their unicellular ancestors. In this work, we identified all GTPases, GEFs and GAPs from genomes representative of the four major taxon groups and investigated their phylogenetic relationships and evolutionary conservation and changes in their functional domain architecture and in their developmental and cell-type specific expression. We performed a hierarchical cluster analysis of the expression profiles of the ~2000 analysed genes to identify putative interacting sets of GTPases, GEFs and GAPs, which highlight sets known to interact experimentally and many novel combinations. This work represents a valuable resource for research into all fields of cellular regulation.

## Introduction

Small GTPases are major regulators of cellular function throughout the eukaryote domain. Also known as small GTP binding proteins or small G-proteins, they bind GTP and hydrolyse it to GDP and undergo a major conformational change when alternating between GTP and GDP bound states. This conformational switch serves to alter the activity of the effector proteins that they interact with. Small GTPases are generally activated by guanine nucleotide exchange factors or GEFs, which assist the exchange of bound GDP for GTP, and inactivated by GTPase activating proteins or GAPs, which activate the usually low intrinsic GTPase activity of the small GTPases.

The small GTPases are subdivided into four major and some minor families that each has their own GEFs and GAPs, although cross-regulation by GEFs and GAPs from other families also occurs. In alphabetical order, these families are the Arf-Sar GTPases with a range of functions in membrane trafficking, motility and gene expression [1], the Rab-Ran family, with Rabs as major regulators of all aspects of endosomal membrane trafficking [2] and Rans as regulators of transport between the nucleus and cytosol [3], the Rho-Rac family, major regulators of actin remodelling [4] and the Ras-Rap family with major roles in cell proliferation and cell adhesion [5]. Misregulation of members of each of the small GTPase families are a major cause of cancer and a range of other diseases, and the mammalian small GTPases have therefore been intensively studied over the past 30 years.

Many members of each of these families are also present in *Dictyostelium discoideum (Ddis*), an organism in Amoebozoa that is unicellular in its proliferative feeding stage, but aggregates to form migrating slugs and spore-bearing fruiting structures when starvefgd. Due to its ease of culture, well-developed strategies for forwards and reverse genetics and broad repertoire of protein imaging strategies and cell-biological and molecular techniques, it has become a popular model for studying most processes that are regulated by small GTPases [6]. Processes common to most eukaryotes such as motility, cell division, phagocytosis and response to infection can be studied in the feeding stage. However, the multicellular phase of its life cycle with its chemotaxis-driven aggregation and morphogenesis, intercellular signalling and cell adhesion, autophagy, cell wall biosynthesis, cell differentiation and programmed cell death requires the repertoire of small GTPase-mediated processes to its fullest extent. The well-orchestrated succession of morphologies and behaviours during its developmental programme eminently lends itself to the identification of a broad range of mutants in small GTPase regulated processes. Such mutants are predominantly non-lethal because they impair multicellular development without affecting unicellular proliferation. Studies using *Ddis* have made fundamental contributions to our knowledge on the roles of small GTPases in cell migration [7,8], particularly highlighting the role of Ras activated PI3 kinase in local actin polymerization and actin wave formation during chemotaxis, cytokinesis, phagocytosis and macropinocytosis [9–11]. The family of Roco GTPases was initially identified in *Ddis* and then found to be widely distributed throughout eukaryotes [12]. However, despite these advantages only 24% of the 164 *Ddis* small GTPases and their 130 GEFs and 115 GAPs have as yet been functionally analysed.

While experimental strategies, such as pull-down of proteins or organelles with appropriately tagged ‘bait’ proteins, followed by mass-spectrometric identification of the ‘catch’ are excellent methods for identification of interacting proteins, they are expensive to perform on the entire range of GTPases. At a fraction of the cost of the experimental approach, bioinformatic analysis of co-regulated expression and evolutionary co-conservation of proteins can provide hints of putative interactions between proteins and their involvement in conserved processes. Conversely, alterations in developmental expression or functional domain architectures between orthologous proteins may point to molecular changes that gave rise to phenotypic innovations.

Molecular phylogenetics divides Dictyostelia into four major taxon groups with *Ddis* residing in group 4 [13,14]. Groups 1–3 consist of species that form relatively small clustered or branched fruiting bodies with maximally two cell types. Many species in these groups have retained encystation of individual amoebas, the ancestral amoebozoan strategy to survive starvation, in addition to sporulation in fruiting bodies. The group 4 species form large and robust fruiting bodies with up to three additional cell types. Their slugs show extensive migration, but as a group they have lost the ability to encyst [15,16].

High-quality genomes as well as developmental and cell-type specific transcriptomes for representatives of each of the four taxon groups of Dictyostelia are available [17–22]. The presence of small GTPases in several of these genomes has already been investigated [17,19,22–24]. However, these studies did not incorporate all or most genomes nor all GTPase families with their GAP and GEF regulators, and no transcriptome data.

In this study, we comprehensively investigated conservation and change in the presence, domain architecture, developmental regulation and cell-type specificity of all GTPases and their GEFs, GAPs and other direct regulators across the four groups of Dictyostelia. We used this information to associate functions of individual GTPases, GAPs and GEFs with specific cell types and developmental stages, and to identify evolutionary trends in gene gain and loss and changes in developmental regulation in the different families of small GTPases and their regulators. For the vast majority of GTPases, the controlling GAPs and GEFs are unknown. We therefore performed a hierarchical cluster analysis of the transcriptome data to establish an ‘interactome’ of similarly expressed GTPases, GEFs and GAPs to guide experimental studies on this major group of signalling proteins.

## Results

### Identification of GTPases, GAPs and GEFs across five dictyostelid genomes

Previous studies of GTPases and their regulators did not involve all group-representative genomes, while several families were not studied at all or in great depth. The group representative genomes used in this work are those of *Dictyostelium fasciculatum* (*Dfas*, group 1), *Polyspondylium pallidum* (*Ppal*, group 2), *Dictyostelium lacteum* (*Dlac*, group 3) and *Dictyostelium discoideum* (*Ddis*, group 4), which are all high quality, almost fully assembled genomes, and *Dictyostelium purpureum* (*Dpur*, group 4) a draft genome, which, like the other genomes, is accompanied by a developmental transcriptome. We generated Interpro scans [25] of all genomes and isolated GTPases, GAPs, GEFs and other regulators from all families by their Interpro identifiers. After the construction of pilot phylogenetic trees, this initial survey was followed up by extensive BLASTp and tBLASTn searches of species proteomes and genomes to identify any missing genes (see Methods and Legends of the annotated trees in supplemental figures S1-S16 for details). The total number of identified genes in each family is listed in Table 1. A few additional genes were identified in families that were previously analysed, but for several families or species, data were not previously available.

**Table 1.** Numbers of GTPases, GEFs and GAPs in group representative dictyostelid genomes

	**GTP-ases**					**GEFs**					**GAPs**				
Family	*Ddis*	*Dpur*	*Dlac*	*Ppal*	*Dfas*	*Ddis*	*Dpur*	*Dlac*	*Ppal*	*Dfas*	*Ddis*	*Dpur*	*Dlac*	*Ppal*	*Dfas*
Arf-Sar	24	22	24	23	22	7	6	6	7	6	12	11	12	12	13
Rab-Ran	74	60	49	49	52	12	12	13	12	12	30	30	31	31	31
Rac-Rho	22	19	15	22	15	46	46	46	44	45	47	44	48	47	47
Elmo						5	5	6	5	7					
Dock						8	8	8	8	8					
Ras	33	19	16	23	28	29	29	27	28	27	15	15	15	18	15
Rap	3	3	4	4	4						11	11	11	11	11
Roco	11	9	9	10	10										
Gpn/Rag/ Miro/Rol	7	7	7	7	7										
Total	175	139	124	138	138	107	106	106	104	105	115	111	117	119	117

**Figure 1.**
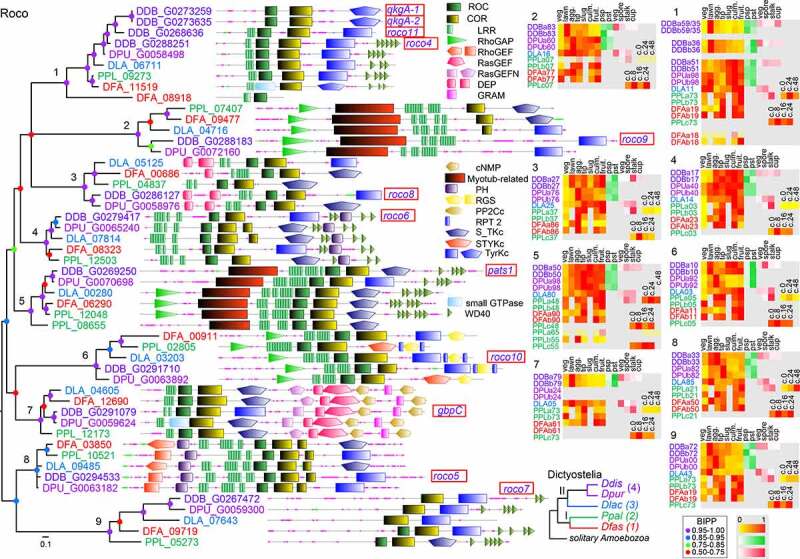
**Conservation and change in the Roco GTPase family**. While most small GTPases only consist of the GTPase domain, the Roco proteins are unusual in combining a Roc (Ras Of Complex) GTPase domain with a COR (C-terminal of Roc) domain and a plethora of other domains. All GTPases in the *Ddis, Dpur, Dlac, Ppal* and *Dfas* genomes were identified from Interpro scans as outlined in Methods, and a pilot phylogenetic tree was prepared from the aligned isolated GTPase sequences. In this tree the Roco GTPases formed a single clade. The sequences in this clade were further supplemented with hits of BLASTp and tBLASTn queries of genomes using Roco sequences as bait. A final tree was then constructed from the Roc sequences by Bayesian inference [26], in which the colour of the gene ID matches that of the species shown in the Dictyostelid phylogeny (lower right). Bayesian posterior probabilities (BIPP) of the nodes are indicated by coloured dots. The tree was annotated with gene names, which were framed in red for genes with known function and with the functional domain architecture of the proteins as analysed in SMART [54]. For overlapping domains, we selected the domain with the lowest E-value. The SMART (full colour) or PFAM (black-shaded rectangle) domain graphics and identifiers are listed in the figure and further domain information can be retrieved from http://smart.embl-heidelberg.de/smart/domain_table.cgi or http://pfam.xfam.org/browse using the identifier as bait. Clades of orthologous proteins and other groupings were further annotated with heatmaps of relative transcript levels at specific developmental stages or in specific cell types, which were retrieved from published RNA sequencing experiments [18,20,21,42] (yellow-red: 0–1 fraction of maximum value), prespore or prestalk cells (white-green: 0–1 fraction of summed reads), or vegetative, spore, stalk and cup cells (white-red: 0–1 fraction of summed reads). Numbers preceded by c. represent hours of starvation in cells set up for encystation. Sets with maximally 10 or less reads are shown in wash-out colour. Note that the phylogeny subdivides the GTPases in clades of conserved orthologs, with orthology further substantiated by similarity of domain architecture. Some *Ddis* genes such as qkgA-1 and qkgA-2 arose from a very recent duplication of part of chromosome 2. For such genes, transcripts were mapped to only one of the replicates, which is indicated by the last two digits of the locus tags of each gene, separated by /.

Final phylogenetic trees were computed by Bayesian inference [26] from alignments of the isolated signature domain sequences of each family (Supplemental_Materials.pdf, Figs. S1-S16). The trees were annotated with the functional domain architecture of the proteins and with heatmaps of the standardized developmental- and cell-type specific expression levels of the genes, as outlined in the example tree for the Roco GTPases in Figure 1. For instance, *roco6* and *pats1* in clades 4 and 5 of this tree are both upregulated in early development in all five species, but show no marked preferential expression in prestalk or prespore cells, and are not consistently upregulated in encystation, or expressed in stalk, spore or cup cells in the fruiting body. Across the other *roco* genes, developmental expression is not well conserved.

In addition to the annotated phylogenies of all GTPase, GAP and GEF families across the major groups of Dictyostelia, the Supplemental Materials section also contains a brief description of the generalized roles of each family across all eukaryotes and a referenced summary of the established functions of specific family members in *Ddis*. The annotated phylogenies should prove useful to researchers in identifying well-conserved genes for which the developmental regulation and cell-type specificity profile suggests that they are likely involved in a stage or cell-type specific function. The presence of functional domains in addition to the signature GTPase, GAP or GEF domains, such as protein kinase or SH3 domains, leucine-rich repeats, WD40 repeats and pleckstrin-homology domains provides further hints of additional signalling activity or specific protein–protein interactions that are associated with the protein of choice.

**Figure 2.**
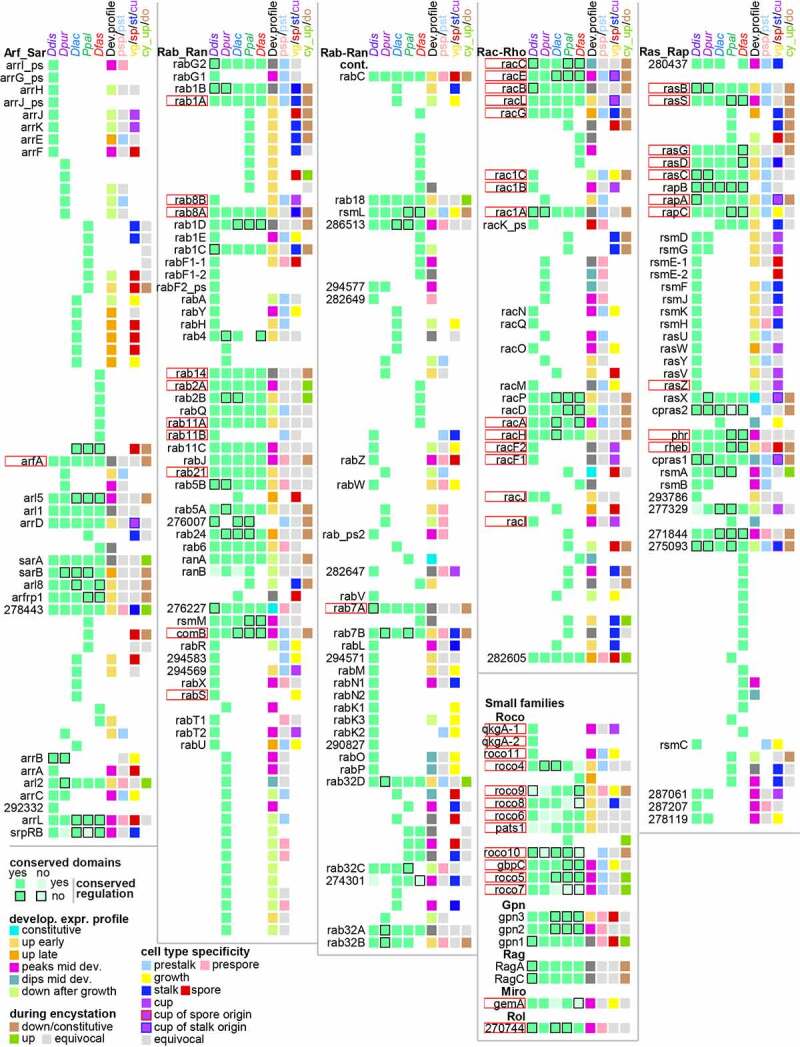
**Summary of conservation and change in Dictyostelid GTPases**. The presence of orthologous GTPases across the *Ddis, Dpur, Dlac, Ppal* and *Dfas* genomes is indicated by green squares in the first 5 columns, which are shown in pale green or with a black border, respectively, when compared to the majority, the functional domains or the developmental regulation are not conserved. Where the number of non-conserved features is larger than 3, pale green or a border is applied to all squares. The colour coding of the 6^th^, 7^th^ and 8^th^ square in each row respectively represent the developmental expression profile in the majority of species, the prestalk/prespore specificity, when conserved between *Ddis* and *Dpur* slugs, the growth, spore or stalk specificity, when conserved between species, and the cup cell specificity in *Ddis*. The 9^th^ square represents up- or down regulation in encystation of *Ppal*. Cup cells are only present in group 4 and are bordered red or blue when the orthologs in group 2 or 3 show spore- or stalk-specific expression, respectively. Grey reflects lack of specificity or conflicting data between species or replicate experiments, and white reflects absence of gene or data. The genes are listed by the *Ddis* gene names or 12 digit Dictybase gene identifiers without the DDB_G0 prefix. Genes with known function in *Ddis* are bordered in red. The gene identifiers and locus tags for the *Dpur, Dlac, Ppal* and *Dfas* genes are listed in Supplemental_Table_S1.xlsx, together with all data on which this figure and Figure 3 are based.

**Figure 3.**
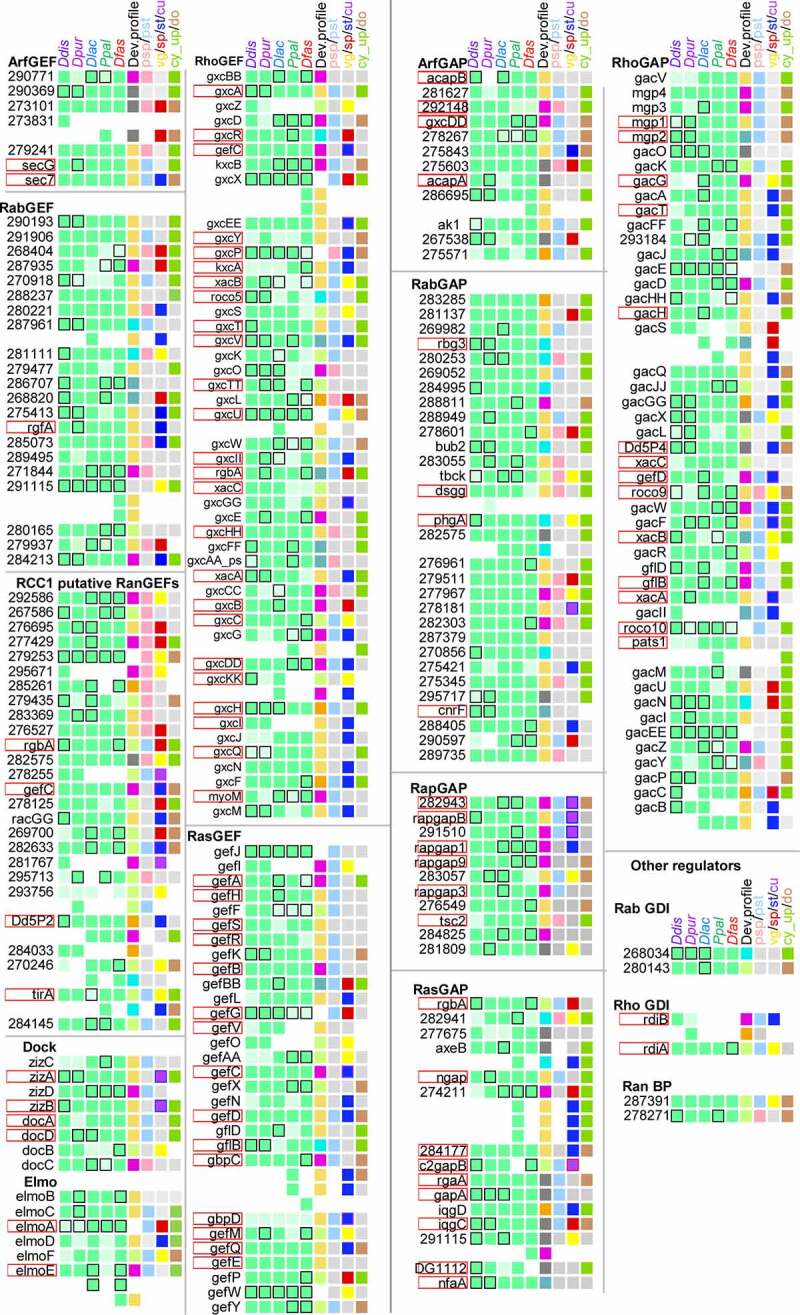
**Summary of conservation and change in Dictyostelid GEFs, GAPs and other GTPase regulators**. Conservation and change in the presence, functional domain architecture and developmental regulation for sets of orthologous GEFs, GAPs and other regulators are summarized in the first five columns of squares, while the majority developmental profile, cell type specificity and regulation in *Ppal* encystation are shown in the next four columns. See Figure 2 and its legend for further explanation. The gene identifiers and data upon which the figure is based are listed in Supplemental_Table_S2.xlsx.

All data on the presence of GTPases and their regulators across species and the conservation of their functional domains and their developmental and cell-type specific expression profiles were compiled in Supplemental_Table_S1.xlsx and Supplemental_Table_S2.xlsx, respectively, and is presented in summary form in Figures 2 and 3.

### Patterns and phylogenetic distribution of gene and gene feature conservation across GTPase families

The compiled data on conservation and change in genes, functional domains and gene expression of the GTPases and their regulators across species also allows to identify evolutionary trends in changes in these features, and trends in cell-type specificity and the stage of development at which members of specific families tend to be expressed. When compared with phenotypic differences between taxon groups, such data may eventually provide hints how molecular change in this group of regulators may have given rise to phenotypic innovation.The preferential expression of specific families at some stages or cell types also provides hints of their involvement in processes unique to that stage- or cell type on one hand, while on the other, they assist to more clearly define the functional role of the cell type.

**Figure 4.**
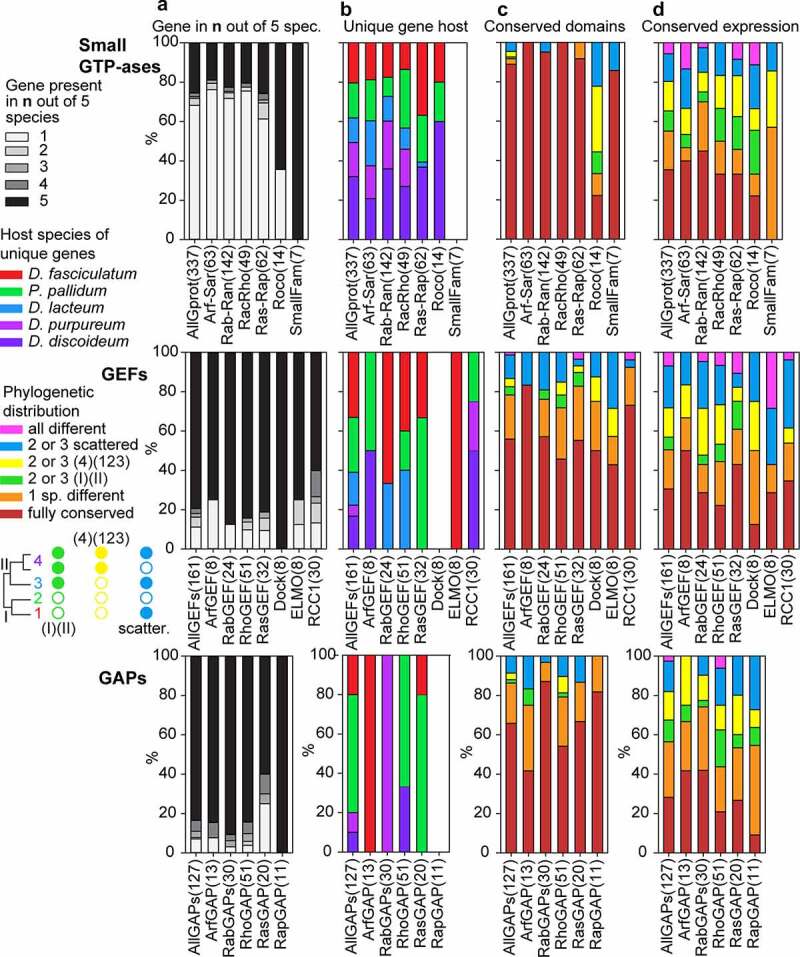
**Conservation profiles of GTPases, GEFs and GAPs**. For each GTPase, GEF or GAP family and all families of each category combined together, we calculated the percentage of the different states of the following features: A. the total number of orthologs out of five species that were conserved for each gene. B. The host species of genes that were unique. C/D. the phylogenetic distribution of genes with conserved domain and conserved regulation, respectively. The name of each family or grouping and its number of members are shown at the X-axis. The figure is based on the data shown in supplemental figures S1-S16 that are compiled in Supplemental_Table_S1.xlsx and Supplemental_Table_S2.xlsx.

**Figure 5.**
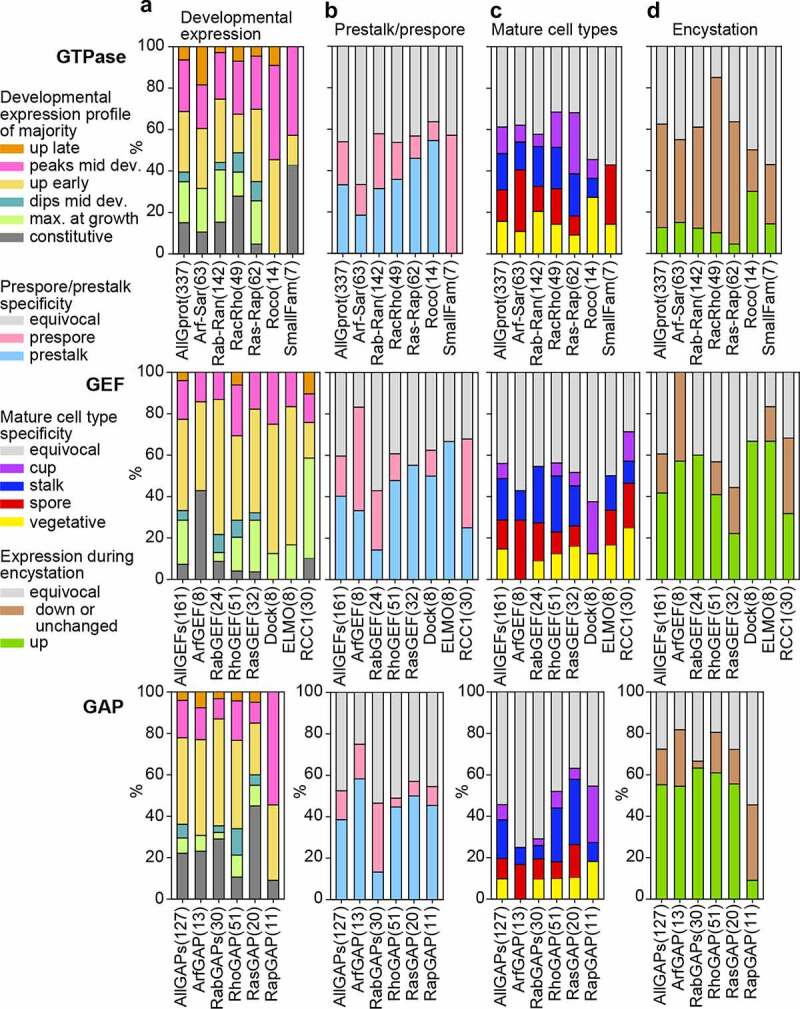
**Developmental expression and cell-type specificity of GTPases, GEFs and GAPs**. For each GTPase, GEF or GAP family and all families of each category combined together, we calculated the percentage of the different states of the following features: A. The developmental expression profile of the majority of genes within orthologous groups. B. Prestalk or prespore specificity in *Ddis* and/or *Dpur* slugs. C. Cell-type specificity in fruiting bodies of the majority of tested species (*Ddis, Dlac* and *Ppal*), compared to vegetative cells. C. Expression during encystation in *Ppal.*

For each recorded feature, we calculated the distribution of the different states of that feature across the individual GTPase, GEF and GAP families and all combined GTPases, GEFs or GAPs (Figures 4 and 5). The most striking difference between the GTPases, on one hand, and the GEFs and GAPs on the other is that the GEFs and GAPs are generally well conserved across all five dictyostelid genomes, while the GTPases of all four large GTPase families show extensive species- or taxon group-specific gene amplification (compare Figures 2 and 3). Only Roco GTPases and other small families are better conserved. Overall, only 26% of GTPases are conserved across all species with 68% being unique to one species (Figure 4a). The gene amplification occurred across all five sequenced genomes, but most extensively in *Ddis* (Figure 4b). For the GEFs and GAPs conservation across all five genomes are 79% and 83%, respectively. The different families of GEFs and GAPs do not seem to have undergone equal gene amplification across species, but due to the low number of amplified genes, the differences may reflect stochastic variation.

Apart from the Roco family, the functional domain architecture of GTPases is over 90% conserved (Figure 4c), which likely reflects that most GTPases only consist of their signature GTPase domain, while the Roco GTPases have many other domains. The GEFs and GAPs are also more domain-rich and show overall 57% and 66% conservation of domains across species, respectively. The altered domain architecture mostly affects only one species or occurs scattered across the phylogeny. The range of functional domains that are associated with GEFs or GAPs is broad. However, the most common are domains that are involved in protein–protein or protein-lipid or phospholipid interactions, such as ANK, LRR, PQQ and WD40 repeats, zinc-finger domains (Znf_CH2, RING, LIM, ZnF_RBZ, zf-MYND, BRCT, TIR, FYVE, BBOX) and other domains (SH3, CH, IQ, GRAM). In addition, domains related to ubiquitin-mediated proteolysis (FBOX, DUSP, UBA_4, FBD) and protein kinase domains are also often found.

The developmental regulation of GTPases, GEFs and GAPs is only conserved across 35%, 31% and 28% of genes, respectively. There are also relatively large contributions of differentially regulated genes that affect only a single species or species scattered across the phylogeny. In the cases that do show clade-specific differences, those in which group 4 is different from groups 1, 2 and 3 are more frequently observed than those where the two more distantly related branches I and II show different developmental regulation (compare the size of the yellow and green bars in Figure 4d).

When comparing specific developmental profiles, respectively, 15%, 8% and 22% of GTPases, GEFs and GAPs are constitutively expressed, while 20%, 22% and 8% of each is only expressed during growth. The remaining 65–70% of the genes are developmentally upregulated, with the GEFs and GAPs mostly being upregulated soon after starvation, and the GTPases showing about equal early upregulation or a peak of upregulation in mid-development (Figure 5a). About half of all GTPases, GEFs and GAP are equally expressed in prestalk and prespore cells, while of the remainder twice more genes are expressed in prestalk than prespore cells. Exceptions are the combined small families (Gpn, Miro, Rag and Rol) of GTPases, which show prespore-specific expression, and the Arf GEFs, the Rab GEFs and GAPs, and the RCC1 proteins, which are preferentially expressed in prespore cells (Figure 5b). Roughly half of the GTPases, GEFs and GAPs are not preferentially expressed in spores, stalk, cup or growing cells. Otherwise, the Rac-Rho and Ras-Rap GTPases and their GEFs and GAPs show preferential expression in the prestalk-derived stalk and cup cells, the Arf-Sar GTPases and their GEFs and GAPs are preferentially expressed in spores, while the Rab-Ran group show a small preference for expression in the prestalk-derived stalk and cup cells (Figure 5c). Rather surprisingly, the majority of GAPs and GEFs are upregulated in *Ppal* encystation, while the GTPases are mostly down-regulated or constitutively expressed (Figure 5d).

### Hierarchical clustering of GTPases, GEFs and GAPs according to their transcription profiles

A subset of GTPases have been experimentally associated with regulation by specific GEFs or GAPs (Supplemental_Table_S3.xlsx) but for the greater majority, the regulatory interactions between GTPases, GEFs and GAPs are unknown. Genes that act together in a complex can be expected to be expressed at the same developmental stage or in the same cell type. Shared developmental and cell-type specific transcription profiles therefore provide information on putative protein interactions and identify yet unknown interactors. We applied hierarchical cluster analysis to associate GAPs and GEFs and other direct regulators with GTPases within and across all families.

The transcription data that were collected for this study were arranged in a linear array for orthologous genes across species, see Supplemental_Table_S4.xlsx, sheet ‘Basic data’. This sheet also collates the data of Supplemental_Table_S3.xlsx on the experimentally established interactions between GTPases and their GEFs, GAPs and other regulators, the subcellular localization of the proteins, the effects of genetic lesions on cellular function, protein association with organelles as determined by proteomics, and transcriptional responses to different food bacteria.

**Figure 6.**
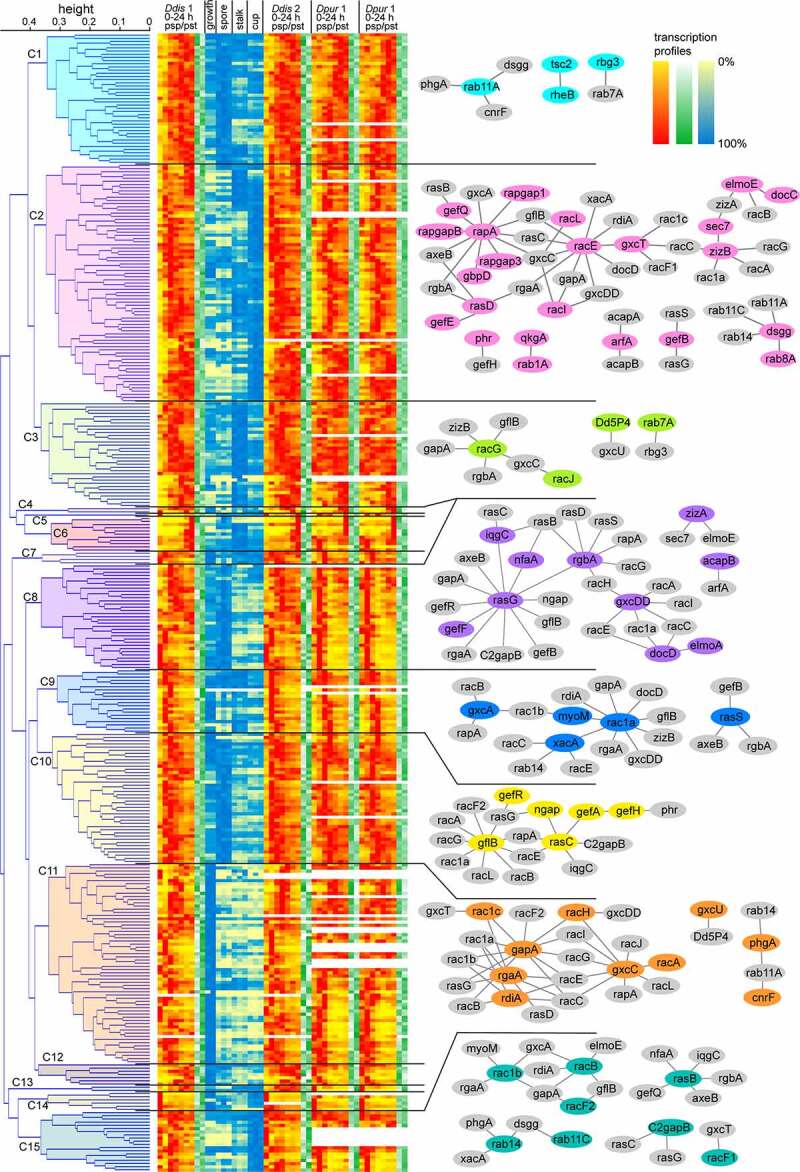
**Hierarchical clustering of GTPases, GEFs and GAPs by expression profile**. Tree obtained by hierarchical clustering of developmental and cell-type specific gene expression data of GTPases and their direct regulators of the group 4 species *Ddis* and *Dpur*, using Pearson correlation to estimate distances between profiles and average linkage to infer the tree. Clusters (C) at a relative branch height of 75% are indicated and heatmaps of all clustered genes are shown. For clusters with >2 known interacting proteins as listed in Supplemental_TableS3.xlsx, sheet 4, the primary interacting partners inside (coloured) and outside (grey) the cluster are visualized as a network using Cytoscape [58]. The complete figure with individual genes annotated with locus tags, interactions, protein function and cellular localization is shown in Supplemental_Fig_S17.pdf and is derived from Supplemental_Table_S4.xlsx, sheet ‘Group4’. A network of all established GTPase and direct regulator interactions is shown in Figure S21.

Hierarchical clustering was performed on the full stage- and cell type-specific transcription profiles of the five *Dictyostelium* species, and on subsets thereof, comprising data from both Branch II (*Ddis, Dpur,* and *Dlac*), Group 4 (*Ddis* and *Dpur*) and *Ddis* only. Distances between profiles were calculated using Pearson correlation and the average linkage algorithm was initially used to infer the trees. Using the Vlookup function in Excel (Supplemental_Table_S4.xlsx, sheet Vlookup), the transcription profiles and the information on protein interactions, localization and function were re-organized to match the gene ordering in the hierarchical trees and copied to sheets ‘Fullprofile’,‘BranchII’, ‘Group4’, ‘Ddisonly’ and ‘Group4Complete’, with the latter inferred by ‘complete linkage’. Figure 6 shows the tree inferred from the Group 4 gene expression data, combined with the ordered expression profiles. Its large size precluded per gene annotation for an A4 size figure, but instead we show networks of established primary interactions for each cluster that contained such interactions. The same tree annotated for each gene with locus tags, primary and secondary interactors (other interactors of the primary partner), as well as the function and localization of the cognate proteins are shown in Supplemental_Fig_S17.pdf.

At 75% branch height, the Group4 and BranchII-based trees are each separated in 15 clusters, while the *Ddis*only and the Full profile based trees separated in 8 and 28 clusters, respectively. Here the height thresholds were altered to yield a similar number of clusters as the other trees. As a means to discriminate between the efficacy of the five clustering analyses to identify interacting proteins, we counted the total number of experimentally established primary and secondary interactions in each cluster. Most (85) interactions were recovered in the Group4-based tree, but the others followed closely behind with 83 or 82 interactions (Table S5). The use of complete linkage clustered only 59 interactions. Trees that were inferred from the different subsets of the data showed different topologies, but a comparison of their cluster content revealed that genes that clustered together in one tree often also clustered together in the other (Supplemental_Fig_S18.pdf). This was particularly the case when comparing the Group4 with the BranchII and *Ddis*only trees, but less so with the Full profile based trees. However, even the latter tree had over 50% of its nodes in common with the other trees (Figure S19).

The purpose of the transcriptome - based cluster analysis is to identify novel interacting networks of GTPases, GEFs and GAPs. Supplemental_Fig_S18.pdf can assist in identifying groups of genes that robustly cluster together, even when different subsets of the transcriptome data are used. Further identification of novel gene interactions can also be guided by the statistical support for the tree nodes that combine genes together. The trees were therefore subjected to approximately unbiassed (AU) and selective inference (SI) bootstrapping [27,28] (Supplemental_FigS20.pdf). The deeper nodes that separate the larger clusters show poor statistical support, but many smaller clusters closer to the tips of the branches are moderate to well supported.

## Discussion

We investigated conservation and change in the small GTPases and their GEFs, GAPs and other direct regulators across the four major taxon groups of Dictyostelia and examined trends in the evolution of their developmental and cell-type specific expression and functional domain architecture. In addition, we performed a hierarchical cluster analysis of the gene expression data to identify sets of genes with similar expression profiles that potentially interact as proteins.

The phylogenetically ordered separate families of all small GTPases, GEFs and GAPs, annotated with developmental expression and functional domains (Supplemental_Materials.pdf, Figs. S1-S16) provides researchers with a complete inventory of all small GTPases and their regulators across Dictyostelia. Conservation across taxon groups of proteins of interest provides a clue to their potential involvement in core regulatory processes. Furthermore, changes in the presence of genes, their developmental regulation or their functional domains yield hints of how their function may have changed in the course of evolution, which together with gene knock-out and gene replacement across species can provide information on how molecular change in this important group of cellular regulators gave rise to phenotypic innovation.

### Small GTPases underwent massive gene amplification, but their regulators did not

The total number of small GTPases in the different families are somewhat higher in taxon group 4 but otherwise similar across Dictyostelia, while the different GEF and GAP families show similar numbers across all taxon groups. For the GEFs and GAPs, this marks their almost complete conservation as orthologs across all taxon groups, but this is not the case for the small GTPases, which underwent extensive gene amplification in individual group-representative species (Figures 2 and 4). A eukaryote-wide study of the Ras-Rap family did not particularly highlight this difference in conservation between GTPases and their regulators [29]. Extensive species-specific expansions of Rab GTPases were observed in Amoebozoa and Excavates [30,31], but GEFs and GAPs were not studied in parallel.

The amplification of GTPase genes in Dictyostelia may mark a species- and/or niche-specific demand for a larger number of small GTPases e.g. to be able to recognize and consume a larger variety of food bacteria or to respond to niche-specific predators, infectious agents or toxins. Some amplified genes are not or poorly expressed under laboratory conditions, which either reflects that the genes are not functional, or only expressed under specific conditions. However, the expression profiles of the amplified unique genes are not markedly different from those of the conserved small GTPases (Table 2), indicating that gene amplification does not occur in response to a single stage-specific challenge. The underlying cause for the GTPase gene amplification is therefore unclear.

**Table 2.** Expression profiles of conserved and unique GTPases

GTPases	% of genes with this expression profile					
	constitutive	decrease after growth	peak in mid. develop.	dip in mid. develop.	up in early develop.	up in late develop.
conserved	18.7	13.2	26.4	2.2	34.1	5.5
unique	14.4	25.5	21.6	6.5	24.8	7.2

### Validity of the newly uncovered putative GAPs and GEFs

The GEFs, GAPs and other regulators of the *Ddis* Rac-Rho and Ras-Rap families were mostly already previously identified [17,19,22–24], and many have been functionally studied by gene manipulation and other experimental approaches (see [7,8,32,33]. However, in the Arf-Sar family, a biological role has only been established for ArfA and for two ArfGEFs and ArfGAPs [34–37], while other GEFs and GAPs were readily identified by their conserved Sec7 and ArfGAP domains, respectively. The Rab-Ran GTPases were least studied in *Ddis*, and their GEFs and most of their GAPs were not previously identified. Metazoan RabGAP activity resides in the well-conserved TBC domain and 31 well-conserved proteins this domain are present across dictyostelid genomes (Fig. S6). For two of those, Dsgg and CnrF, RabGAP activity on Rab8A and Rab11A, respectively, was demonstrated [38–40].

In Metazoa, RabGEF activity is associated with a wide range of structurally divergent proteins, such as the ~12 subunit TRAPP (TRAnsport Protein Particle) I, II, III complexes, which are conserved throughout eukaryotes [41] and proteins with VPS9 and DENN domain. We identified 8 and 18 well-conserved DENN and VPS9 domain proteins in Dictyostelia, respectively, as well as 4 close homologs to other metazoan RabGEFs (Fig. S5). However, no RabGEF activity has been established for any of the *Ddis* proteins. Their assignment as putative RabGEFs should therefore be treated with caution, but simultaneously as a wide-open field for gene function discovery.

### Rac-Rho and Ras-Rap GTPase function is particularly associated with prestalk cells

The evolutionary trends in conservation of GTPases, GEFs and GAPs were unremarkable, with most, functional domains and expression profiles being conserved. As is the case for transcription factors, the only other large family of cellular regulators that was analysed in the same manner [42], there is a tendency for expression profiles of GTPase-related genes to be more different between taxon group 4 and the other three groups, than between the phylogenetically more distant branches I and II that contain groups 1 & 2 and groups 3 & 4, respectively (Figure 4d). Such a trend is not found for differences in functional domains, but is correlated with group 4 being phenotypically the most distinctive. As previously suggested, this could mean that changes in gene expression played a larger role in phenotypic innovation than those in functional domains.

A marked difference between transcription factors and GTPase-related proteins, is that two times more transcription factor genes were expressed in prespore over prestalk cells, whereas almost the opposite is true for the GTPase-related genes. For the mature cell types, both the transcription factors and GTPase-related genes show as groups slightly higher expression in stalk over spore cells. The prestalk preference is particularly high for the Roco proteins and for Rac-Rho and Ras-Rap GTPases and their GEFs and GAPs, whereas the Rab-Ran GTPases are less prestalk-enriched, and their GEFs and GAPs distinctly prespore- enriched. Their prestalk preferences likely reflect the dominant role of the Rac-Rho and Ras-Rap GTPases in regulating the actin cytoskeleton in coordinated cell migration, which in slugs largely depends on the prestalk population [43–45]. Both the relatively low dependence of prestalk cells on novel transcription factors and their high dependence on Rac-Rho and Ras-Rap GTPases suggest that this population is much more involved in morphogenetic cell movement than preparing itself for stalk cell differentiation.

Prespore cells are at this stage involved in the expression of spore coat genes and in laying down the first layer of the spore wall and synthesizing spore wall precursors in Golgi derived vesicles [46,47]. Since the Rab GTPases are major coordinators of vesicle trafficking, among which the transport of vesicles from the endoplasmic reticulum through the Golgi apparatus [2,48], the relative prespore enrichment of this family and its regulators may reflect the involvement of Rabs in prespore vesicle formation.

### Towards elucidation of the GTPase interactome

Proteins that act in complexes, like GTPases and their GAPs and GEFs, have to be expressed at the same developmental stage and in the same cell type to be able to do so. Information on shared transcriptional regulation of their cognate genes can therefore help to recognize interactions between specific proteins. To this end, we subjected the extensive transcription data that we collected for all GTPase-related genes to statistical procedures to identify clusters of similarly related genes. We estimated the efficacy of different clustering methods and subsets of the transcription data by examining to what extent experimentally recognized interacting proteins are also clustered in our analysis. Use of only the group 4 (*Ddis* and *Dpur*) transcription data recovered most of the known interactions in clusters, but use of larger and smaller subsets performed almost equally well and about half of the genes consistently clustered together. If known interacting proteins do not occupy the same cluster, their transcription heatmaps were quite obviously different, which likely means that some interaction partners are used over a wider range of stages and cell types than their counterparts.

The known interactions are mainly between members of the Ras-Rap and Rho-Rac families and they were mostly recovered from clusters C2/C8 and C11, respectively (Figure 6). Cluster C2 contains genes that peak during aggregation and show specificity for stalk and/or cup cells. Cluster 8 contains genes with peak expression in mid-development or growth and early development and show highest expression in both spores and growing cells. Cluster 11 contains genes which are downregulated after growth and early development and are mostly weakly expressed in spore, stalk and cup cells. Only a few known interactions were recovered from clusters that showed upregulation in later development. This bias likely reflects the focus of workers in the *Dictyostelium* field on directed cell migration and regulation of the actin cytoskeleton, which play a major role in aggregation, and in cytokinesis and phagocytosis in the growth stage. GTPase-related proteins in these processes may yet be uncovered by scrutiny of other members of clusters C2, C8 and C11, while the developmental profiles of the remaining clusters may hint at requirements of specific GTPases in slug and fruiting body morphogenesis, and processes like phototaxis and thermotaxis.

In addition, there is still a wide-open field of discovery for elucidation of functions for the Rab-Ran and Arf-Sar families in processes like phagocytosis, autophagy and cell wall biosynthesis that have a strong stage- and/or cell-type specific component. With respect to the Ran GTPases, cluster 14 is of interest. This cluster harbours just six genes but five of them are related to proteins of the Ran pathway, including the two Ran homologs, RanA and RanB, RanBP1 and two uncharacterized genes, DDB_G0278125 and DDB_G0269700, encoding proteins with RCC1 domains characteristic of Ran GEFs. RanBP1 is well conserved across eukaryotes and acts as a guanine nucleotide dissociation inhibitor and as a RanGAP cofactor. Interestingly, both RanA and RanBP1 are localized to the bacteria-containing vacuole upon infection with *Legionella* [49], further strengthening a possible physical interaction. Of the RCC1 proteins, the first has no obvious orthologs in higher eukaryotes, but the second shares domain architecture with human RCBTB1, a protein mutated in a rare form of retinopathy for which an effect on Ran was postulated [50,51]. These two RCC1 proteins emerge as strong candidates for future studies aimed at identifying Ran GEFs in *Ddis*.

However, the biological roles of many of the 400 proteins described here may be relatively minor or overlapping with other family members. Furthermore, important constitutively expressed GEFs or GAPs may interact with several GTPases that show more stage- and cell-type specific expression profiles or *vice versa*, in which cases their interactions are not evident from the cluster analysis. The results of this analysis should therefore be interpreted and used with caution and preferably only to substantiate information acquired from other approaches.

## Methods

### GTPase sequence retrieval

Small GTPases were isolated from Interpro scans [25] of the most recently annotated proteomes of *Ddis, D. purpureum* (*Dpur)* (http://dictybase.org/Downloads/), *D. lacteum* (*Dlac), Polysphondylium pallidum* (*Ppal)* and *D. fasciculatum* (*Dfas)* (http://sacgb.leibniz-fli.de/cgi/index.pl?ssi=free) using the InterPro identifiers IPR001806, IPR002041, IPR003578, IPR020849, IPR021181 and IPR006689 for generic, Ran, Rho, Ras, Miro and Arf GTPases, respectively. Due to the considerable sequence similarity overlap between different categories of GTPases, a single sequence alignment was made of all retrieved proteins using Clustal Omega [52] with five combined iterations, and a pilot tree was inferred by RaxML [53]. This tree subdivided the sequences into four large groupings, which each mostly contained members of Arf-Sar, Rab-Ran, Rac-Rho or Ras-Rap families of GTPases, respectively, and some smaller clades that included the Roco, Miro, Rag, Gpn and Roco-like GTPases. BLASTp searches were performed with clade-representative members within each of the groupings to retrieve any genes that were not detected by the InterPro scans. New alignments were prepared for each of the major and minor groups, and phylogenies were inferred by Bayesian analysis [26]. These trees were scrutinized for any missing members of otherwise orthologous sets, and further BLASTp or tBLASTn searches were performed with a member of the set as bait. Final Bayesian trees were then inferred including the additional hits, using a mixed amino-acid model with rate variation between sites predicted by a gamma distribution. Analyses were run for 1 to 10 million generations but often did not fully converge due to the relatively small number of variable sites in the GTPase domains. Orthologous clades were generally well resolved but deeper nodes of the trees were not.

### Sequence retrieval for GTP-ase regulators

The functional domains of GEFs, GAPs and other regulators of small GTPases are distinctive, and thus far specific for the different subtypes of GTPases. These proteins were identified in the first round from InterPro scans using the InterPro identifiers of their GTPase regulatory domain as indicated in the legends to figures S2-S15 in Supplemental_Materials.pdf. After inferring a pilot tree for each regulator type, BLASTp and tBLASTn searches of proteomes and genomes were performed to identify any missing members of the family as described above and a final tree was inferred by Bayesian analysis.

### Phylogenetic tree annotation

The functional domain architectures, including PFAM domains, signal peptides and internal repeats were analysed in SMART [54], saved as .svg files and juxtaposed to the protein locus tags at the tips of the phylogenetic tree branches. SMART or PFAM domain identifiers are listed in each figure and domain descriptions can be retrieved from http://smart.embl-heidelberg.de/smart/domain_table.cgi or http://pfam.xfam.org/browse. Clades of orthologous genes or other groupings were annotated with relative transcript levels at specific developmental stages or in specific cell types, which are shown as heat maps that represent the fraction of the maximum transcript read count for the developmental profiles and the fraction of the summed read counts for the cell types. The normalized reads were retrieved from published RNA sequencing experiments [18,20,21,42].

### Hierarchical clustering

The full set or subsets of transcriptome data for *Ddis* genes and their orthologs in *Dpur, Dlac, Ppal* and *Dfas*, were re-ordered into a linear array and subjected to hierarchical clustering in Orange 3.27.1 [55], generally using Pearson correlation as the distance metric and average linkage to infer the tree. Data from individual experiments were included for *Ddis* and *Dlac* cell-type specific transcripts, rather than the averaged values used in Figure 1 and Figs. S1-S16. Data were standardized as percentage of the maximum value of a set in general, or of the sum of values, when there were only two. Hierarchical trees were bootstrapped with 1000 bootstrap replicates using the pvclust package in R [28].

In order to compare trees obtained from different subsets of the transcription profiles, two trees were juxtaposed using the tanglegram function in the dendextend package in R [56]. Correlations between common nodes in tree comparisons were calculated and plotted using the dendextend and corrplot packages in R.

### Retrieval of data on protein interaction, function and subcellular localization

Data on protein interactions, subcellular localization and phenotypes or components of Rho signalling were collected from [8]. This dataset and data for other and/or more recently studied GTPases and regulators were retrieved from publications listed on the corresponding gene page in Dictybase (http://dictybase.org/) [57]. The information is compiled in Supplemental_Table_S3.xlsx. Networks of interacting proteins were visualized using Cytoscape [58].

## Supplementary Material

Supplemental Material

## Data access

All original sequence data used in this work were previously archived in the European Nucleotide Archive and/or Genbank with earlier publications. All processed data are provided in the Supplemental spreadsheets Supplemental_Table_S1.xlsx, Supplemental_Table_S2.xlsx, Supplemental_Table_S3.xlsx and Supplemental_Table_S4.xlsx. Supplemental figures S1-S16, S19 and S20 are provided in Supplemental Materials.pdf. Supplemental figures S17, S18 and S19 are provided as separate .pdf files due to their large size.
