# Supplementary material for: Interactome and evolutionary conservation of Dictyostelid small GTPases and their direct regulators
Source: Small GTPases. 2021 Oct 5;13(1):239–54. doi: 10.1080/21541248.2021.1984829 (PMC8923023; doi:10.1080/21541248.2021.1984829)
Supplement: Supplemental Material [file KSGT_A_1984829_SM5747.zip › supplementary/Supplemental_Fig_S18.pdf]

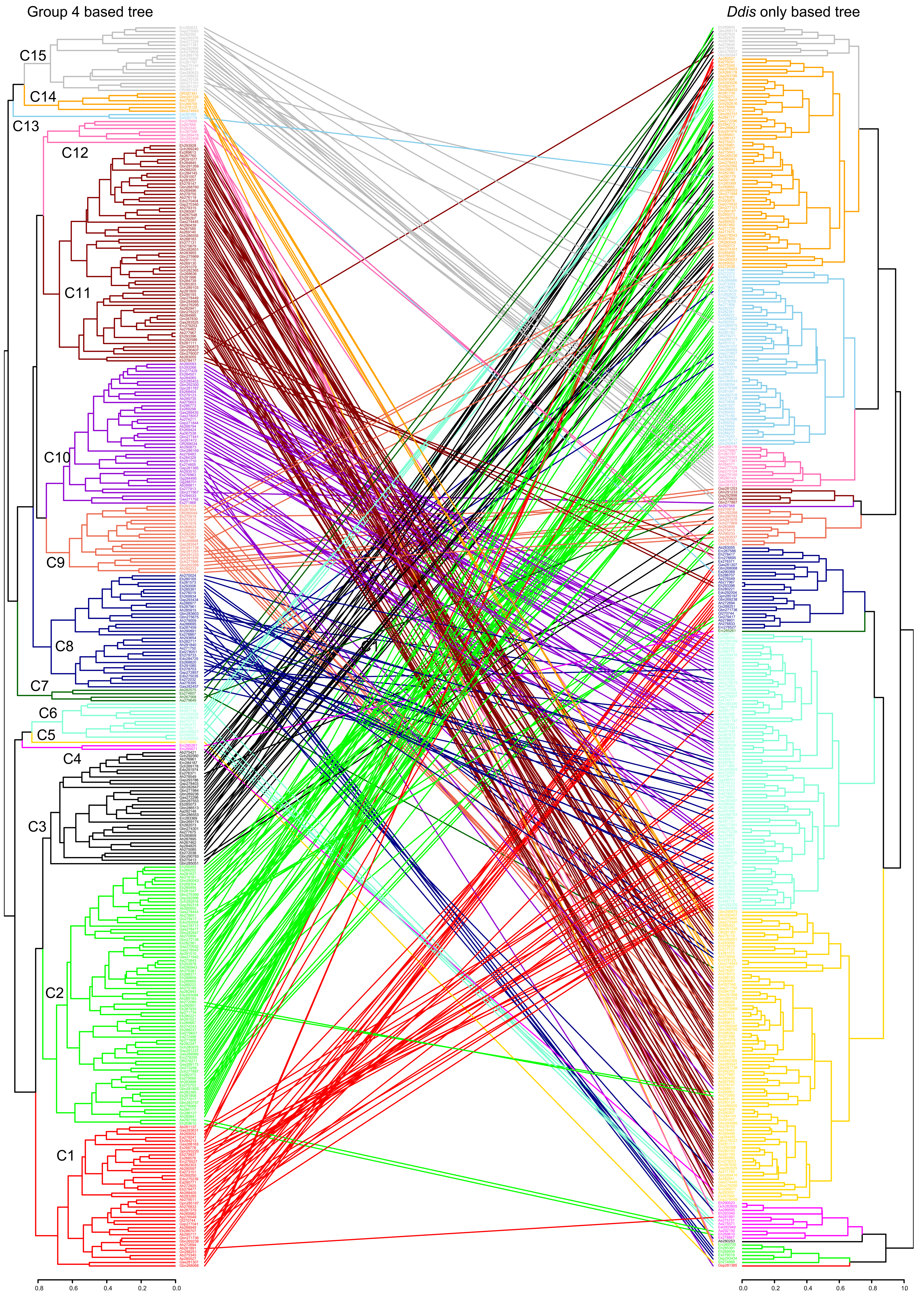
Group 4 based tree
Ddis only based tree
C15
C14
C13
C12
C11
C10
C9
C8
C7
C6
C5
C4
C3
C2
C1
0.8
0.6
0.4
0.2
0.0
0.0
0.2
0.4
0.6
0.8
1.0

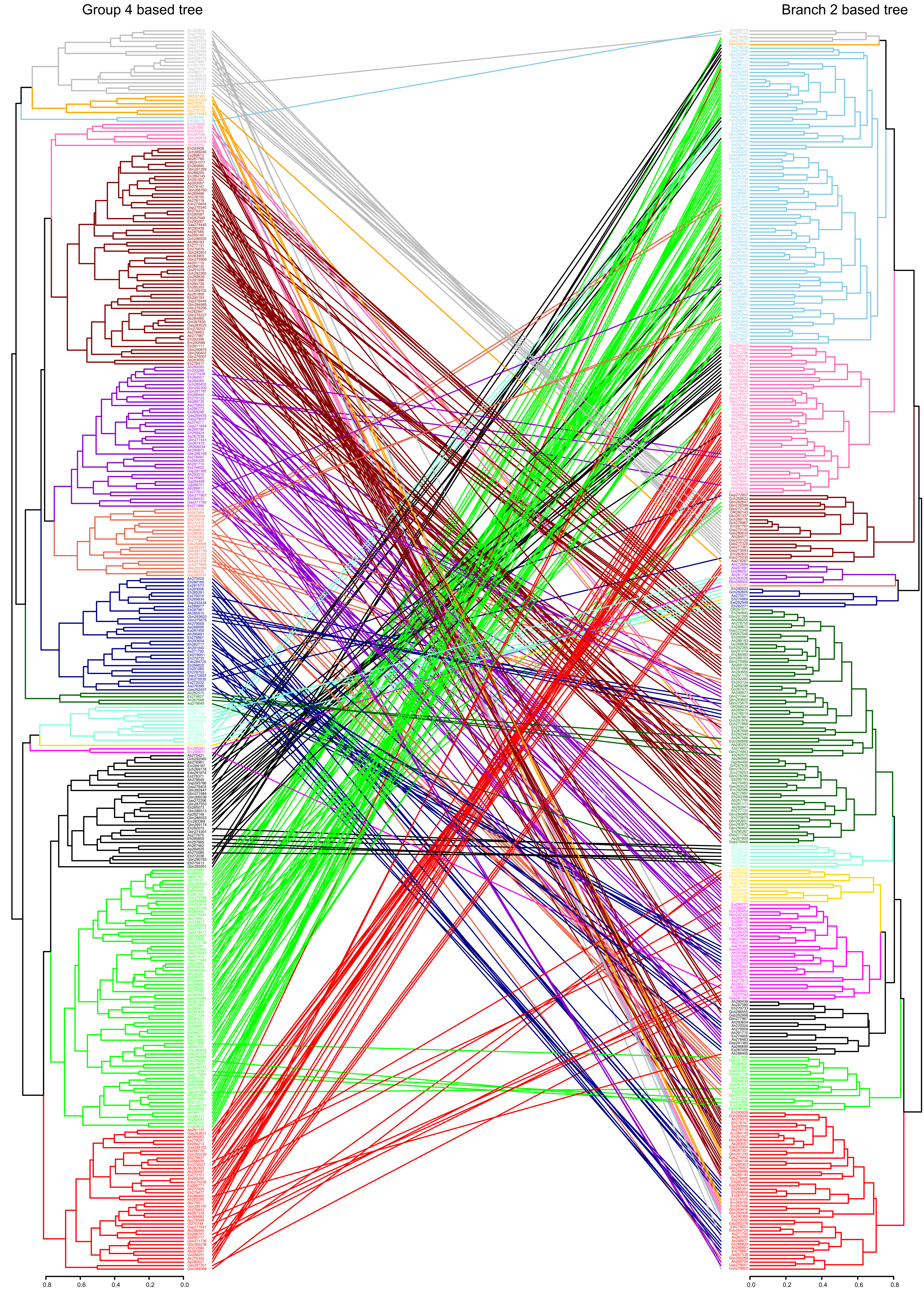

Group 4 based tree
Branch 2 based tree
0.8
0.6
0.4
0.2
0.0
0.0
0.2
0.4
0.6
0.8

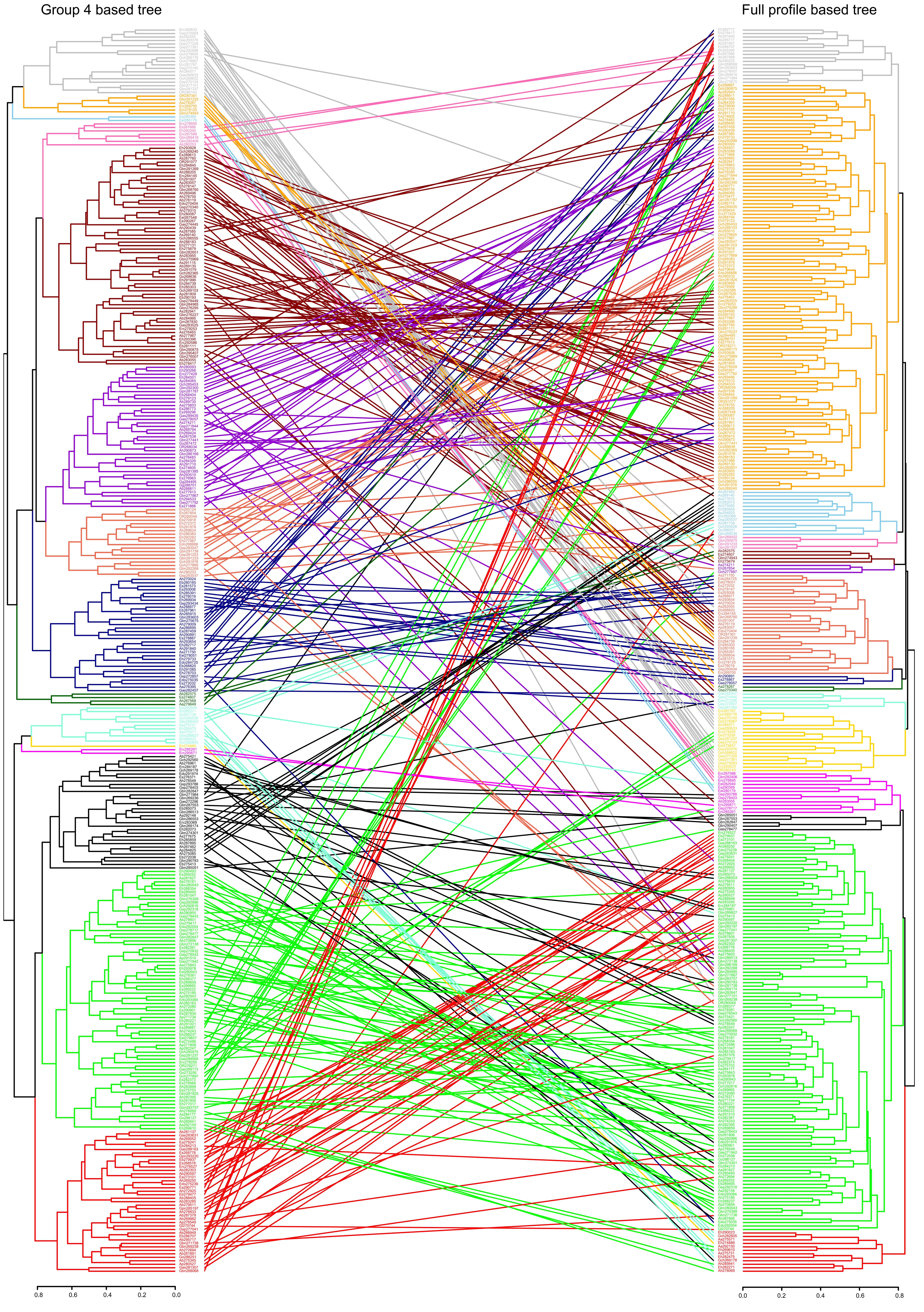
Group 4 based tree
Full profile based tree
