# Supplementary material for: Interactome and evolutionary conservation of Dictyostelid small GTPases and their direct regulators
Source: Small GTPases. 2021 Oct 5;13(1):239–54. doi: 10.1080/21541248.2021.1984829 (PMC8923023; doi:10.1080/21541248.2021.1984829)
Supplement: Supplemental Material [file KSGT_A_1984829_SM5747.zip › supplementary/Supplemental_Materials.pdf]

SUPPLEMENTAL FIGURES AND TABLES

Table of Contents

Arf-Sar family GTPases 2
Arf guanine nucleotide exchange factors 2
Arf GTPase activating proteins 2
Figure S1. Conservation and change in the Arf-Sar GTPases across Dictyostelia. 4
Figure S2. Arf Guanine Nucleotide Exchange Factors 5
Figure S3. Arf GTPase Activating Proteins 6
Rab and Ran GTPases 7
Rab guanine nucleotide exchange factors 7
Rab GTPase activating proteins and guanine nucleotide dissociation inhibitors 7
Figure S4. Conservation and change in the Rab-like GTPases across Dictyostelia. 10
Figure S5. Rab Guanine Nucleotide Exchange Factors 12
Figure S6. Rab GTPase Activating Proteins and GDP Dissociation Inhibitors 14
Ran GTPases 15
Ran guanine nucleotide exchange factors 15
Ran GTPase activating proteins 15
Figure S7. Putative Ran regulators 16
Rho-RAC GTPases 17
Rho guanine nucleotide exchange factors 17
Rho GTPase activating proteins 18
Rho guanine nucleotide dissociation inhibitor 18
Figure S8. Conservation and change of Rac-Rho GTPases across Dictyostelia 20
Figure S9. Rho Guanine Nucleotide Exchange Factors – DH family 23
Figure S10. Rho Guanine Nucleotide Exchange Factors – Dock-Elmo family 24
Figure S11. Rho GTPase Activating Proteins and Rho GDP-Dissociation Inhibitor 27
Ras and Rap GTPases 28
Ras guanine nucleotide exchange factors. 28
Ras GTPase activating proteins 28
Rap GTPase activating proteins 29
Figure S12. Conservation and change in the Ras-like GTPases across Dictyostelia. 31
Figure S13. Ras Guanine Nucleotide Exchange Factors 32
Figure S14. Ras GTPase activating proteins 33
Figure S15. Rap GTPase Activating Proteins 34
Small families of GTPases 35
Figure S16. Roco, Gpn, Rag, Miro and Rol GTPases across Dictyostelia. 36
Cluster analysis 37
Figure S17. Annotated hierarchical tree of group 4 transcription profiles. 37
Figure S18. Comparison of hierarchical trees 37
Figure S19. Common nodes in hierarchical trees derived from different transcriptome subsets 37
Figure S20. Statistical support for clustered genes 37
Figure S21. Network of established interactions between GTPases and their primary regulators 38
SUPPLEMENTAL TABLES 39
Supplemental_Table_S1.xlsx 39
Supplemental_Table_S2.xlsx. 39
Supplemental_Table_S3.xlsx. 39

Supplemental_Table_S4.xlsx. 39
Supplemental_Table_S5. Recognized interactions per cluster over different hierarchical trees 40
REFERENCES 41

ARF-SAR FAMILY GTPASES

The Arf-Sar family GTPases control key functions in vesicle transport, cell movement, cell division and gene expression. Together with the Rab GTPases, they have a particularly dominant role in membrane trafficking from the endoplasmic reticulum (ER) through the Golgi and trans Golgi network (Thomas and Fromme 2020). The Arf-GTPases regulate target proteins in many ways, such as recruitment to membranes and by causing conformational change that alters enzyme activity or binding to other molecules. The Arf family differs from other GTPases in harbouring a short N-terminal extension that is covalently modified by myristoylation, palmitoylation or acetylation. The mammalian Arf-Sar family is subdivided into 6 true Arfs (ADP-Ribosylation Factors), 21 Arf-like factors (Arl), 2 Sar (Secretion-Associated and Ras-related) proteins and Trim23 (Sztul et al. 2019).

Ddis has 1 Arf protein (ArfA), 2 Sars, 6 Arls and 12 proteins annotated as Arr (Arf-related). While the Arf, Sars and Arls are conserved throughout Dictyostelia, this is only the case for two of the Arr proteins. The other Arrs result from extensive gene amplification in individual species or taxon groups (Fig. S1). Many of the amplified genes are not or very poorly expressed throughout growth and development, suggesting that their functions may only be required under specific conditions. Functional information is thus far only available for *Ddis* ArfA, which induces a cytokinesis defect when expressed as a constitutively active mutant form (Dias et al. 2013).

Arf guanine nucleotide exchange factors

The ArfGEFs that exchange GDP for GTP, and thereby activate mammalian Arfs, harbour a defining Sec7 domain that causes the nucleotide exchange and a range of other domains that mediate their specific cellular roles. The Arl GTPases are not or rarely regulated by the Sec7 proteins (Casanova 2007; Sztul et al. 2019). The 15 human ArfGEFs are divided into 6 subtypes. The naming of some ArfGEFs is based on their sensitivity to inhibition by brefeldin A, a toxin that blocks secretion, such as the Golgi-associated brefeldin A-resistance factor (GBF) and brefeldin A-inhibited (BIG) ArfGEFs.

The Dictyostelia contain 6 deeply conserved ArfGEFs (Fig. S2). The proteins in clades 1-3 share the Sec7 domain, an N-terminal DCB domain and a Sec7_N domain with the mammalian GBF1 and BIG proteins, both key regulators of endosomal and secretory pathways (Casanova 2007; Sztul et al. 2019). The proteins in clades 4-6 share a C-terminal PH domain with the cytohesin, EFA6 and BRAG1 mammalian ArfGEF subtypes. Two of the *Ddis* proteins have been studied. Sec7 is located both in the cytosol or at the plasma membrane in regions not involved in pseudopod, phagosome or pinosome formation. Deletion of *sec7* caused impaired phagocytosis and reduced speed and persistence of cell migration (Muller et al. 2013). SecG was detected only in the cytosol. Mutants lacking *secG* developed normally into fruiting bodies, but showed a mild defect in adhesion to the substratum, and a small reduction in movement speed during chemotaxis (Shina et al. 2010).

Arf GTPase activating proteins

The GTPase activating proteins (GAPs) for the Arf GTPases share a conserved ArfGAP domain and a range of subtype-specific domains that mediate their localization and function. In addition to terminating GTPase activity, the ArfGAPs may also act as downstream Arf effectors (Sztul et al. 2019). According to their functional domain architecture, the ~28 mammalian ArfGAPs have been subdivided into 10 subtypes. However, only two members of the ACAP subtype, in which the ArfGAP domain is flanked N-terminally by a BAR and a PH domain and C-terminally by ankyrin (ANK) repeats, can be recognized among 12 Dictyostelid ArfGAPs (Fig. S3). The mammalian ACAPs 1, 2 and 3 have roles in actin remodeling during cell ruffling, phagocytosis and neurite outgrowth, with ACAP2 and 3 acting on Arf6 (Tanna et al. 2019).

Single and combined knock-out of *Ddis acapA* and *acapB* yielded cells with normal growth and development into fruiting bodies. However, the *acapA⁻/acapB⁻* spores were less detergent resistant than wild-type spores, usually a result of defective spore wall synthesis. *acapA⁻/acapB⁻* also showed more but shorter actin-based protrusions after cAMP pulsing, but otherwise normal chemotaxis and cell streaming (Chen et al. 2010). Other workers reported defects in cytokinesis, cell migration and lysosome maturation in *Ddis acapA⁻* mutants that were attributed to defective ArfA mediated actin remodeling (Dias et al. 2013; Bailo et al. 2014). The *Ddis* ArfGAP DDB_G0292148 was identified as the gene disrupted in a facultative cheater mutant (Santorelli et al. 2008). The remaining investigated ArfGAP, gxcDD, also harbours a RhoGEF domain as well as CH, IQ and PH domains.

The CH domain localizes to the plasma membrane, the RhoGEF domain interacts with several Rac GTPases, while the ArfGAP-PH domain binds to PtdIns(3,4,5)P at the leading edge of the cell. Deletion of *gxcDD* causes a mild defect in aggregation streaming (Mondal et al. 2007).

Overall, compared to other classes of small GTPases and their GEFs and GAPs, the Arfs and their regulators are vastly underinvestigated in *Dictyostelium.* The deep conservation of particularly the GAPs and GEFs, inclusive of their domain architecture and developmental regulation, suggests that they have important cellular and developmental roles to play.

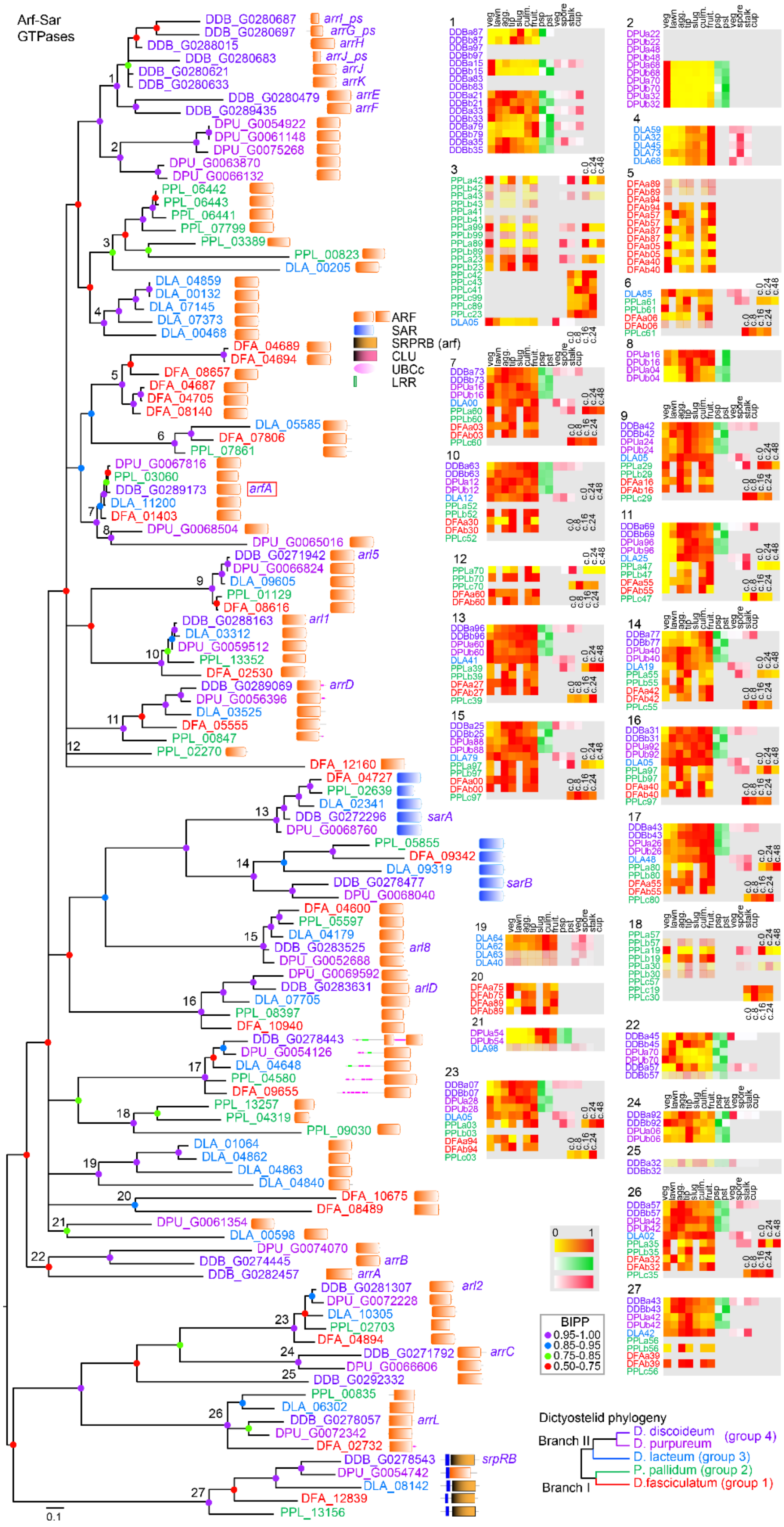

Figure S1. Conservation and change in the Arf-Sar GTPases across Dictyostelia.

GTPases in the *Ddis*, *Dpur*, *Dlac*, *Ppal* and *Dfas* genomes were identified from Interpro scans as outlined in Methods and a pilot phylogenetic tree was prepared from all aligned sequences. In this tree the Arf-Sar GTPases are presented as a single clade. The sequences in this clade were further supplemented with hits of BLASTp and tBLASTn queries of genomes using Arf-Sar sequences as bait. A final tree was then constructed by Bayesian inference (Ronquist and Huelsenbeck 2003), in which the colour of the gene ID matches that of the species shown in the Dictyostelid phylogeny. Bayesian posterior probabilities (BIPP) of the nodes are indicated by coloured dots. The tree was annotated with the functional domain architecture of the proteins as analysed in SMART (Schultz et al. 1998) and gene names, which were framed in red for genes with known function. Clades of orthologous proteins and other groupings were further annotated with heatmaps of relative transcript levels at specific developmental stages or in specific cell types, which were retrieved from published RNA sequencing experiments (Gloeckner et al. 2016; Kin et al. 2018; Forbes et al. 2019; Parikh et al. 2010). Temporal expression (yellow-red: 0-1 fraction of maximum value), prespore or prestalk cells (white-green: 0-1 fraction of summed reads), or vegetative, spore, stalk and cup cells (white-red: 0-1 fraction of summed reads). Numbers preceded by c. represent hours of starvation in cells set up for encystation. Sets with maximally 10 or less reads are shown in wash-out color.

Note that across all trees some *Ddis* genes are present as replicates that result from a recent duplication of a segment of chromosome 2 in *Ddis* strain AX4 that was used for genome sequencing. Since transcripts can be mapped to only one of the replicates, their heatmap label incorporates the last two digits of both gene locus tags (e.g. clade 3 in fig. S2).

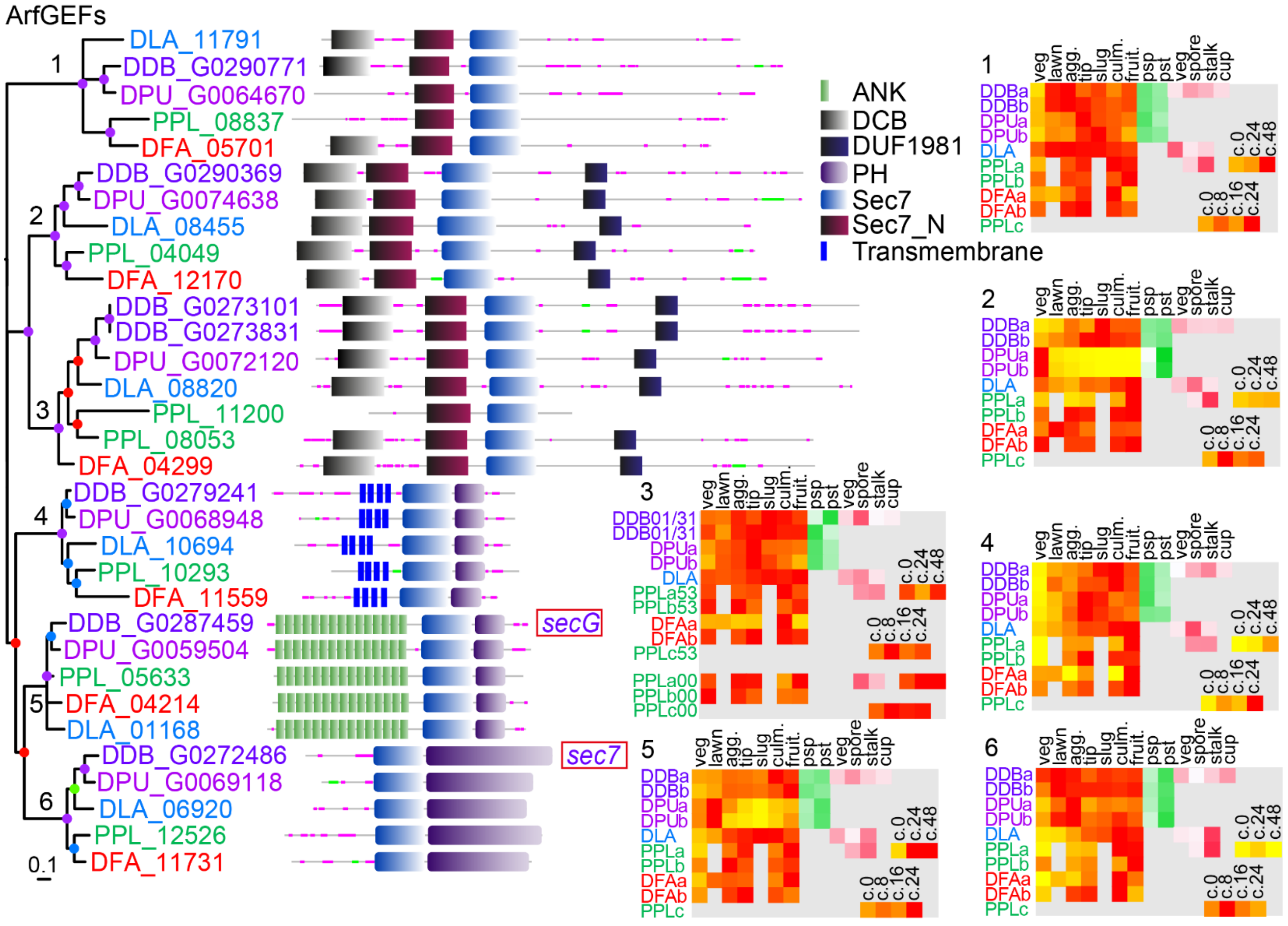

Figure S2. Arf Guanine Nucleotide Exchange Factors

Putative ArfGEFs were identified as proteins containing the Sec7 GEF domain by query of Interpro scans of the five Dictyostelid proteomes with the domain identifiers IPR000904, IPR023394, and IPR035999 and BLAST searches as detailed in Methods. A phylogenetic tree was inferred from the isolated Sec7 domain sequences from all retrieved proteins and annotated with protein functional domains and heatmaps of developmental and cell-type specific expression of the genes as outlined in Methods and the legend to Fig. S1. DDB_G0273101 and DDB_G0273831 in clade 3 result from a recent duplication of a segment of chromosome 2 in *Ddis* strain AX4 that was used for genome sequencing. Since transcripts can be mapped to only one of the duplicate genes, their heatmap label incorporates the last two digits of both gene locus tags.

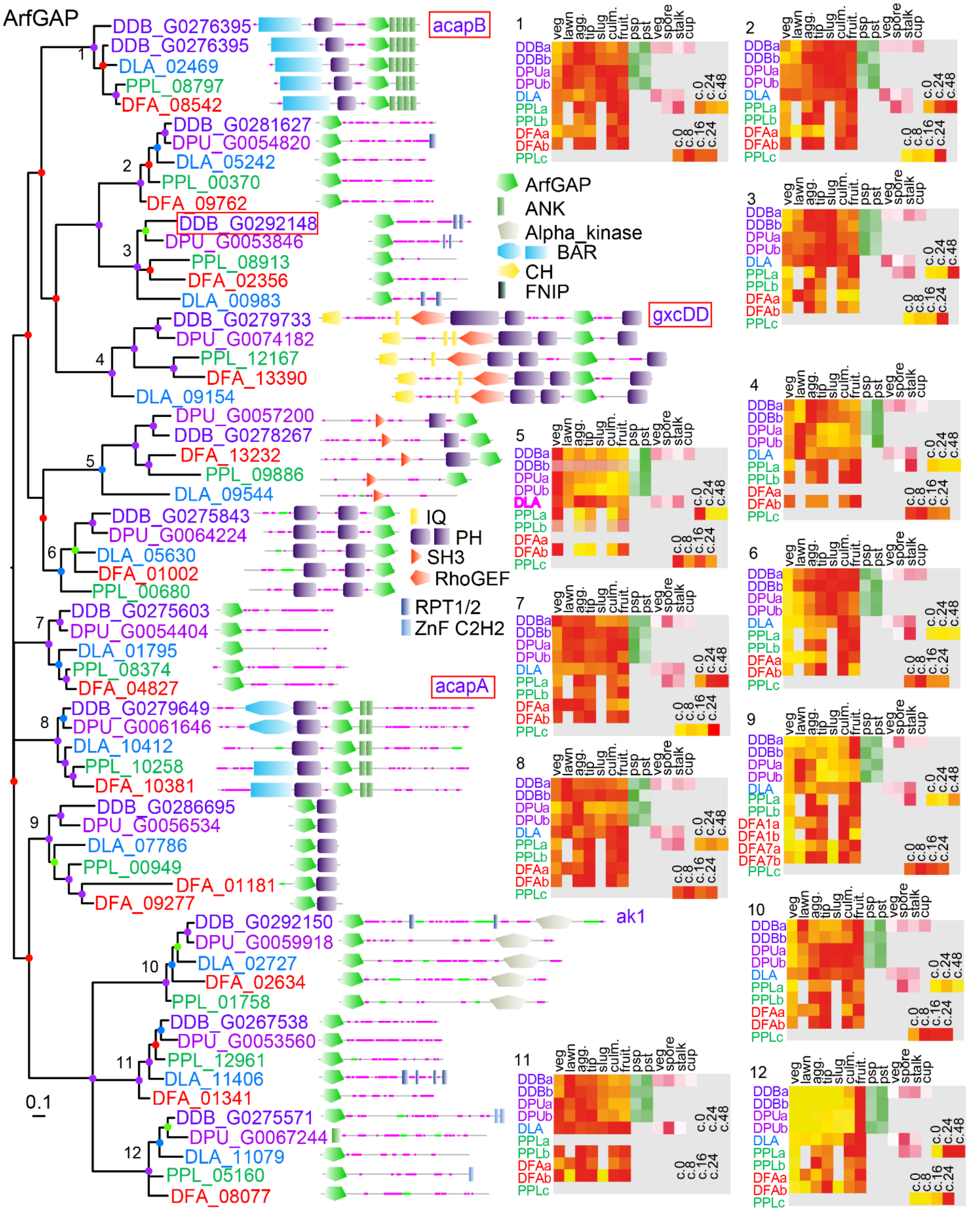

Figure S3. Arf GTPase Activating Proteins

Putative ArfGAPs were identified as proteins containing the ArfGAP domain by query of Interpro scans of the Dictyostelid proteomes with identifiers IPR001164 and IPR038508 and BLAST searches as detailed in Methods. A phylogenetic tree was inferred from the isolated ArfGAP domains, and annotated with protein functional domains and heatmaps of developmental and cell-type specific expression of the genes as outlined in Methods and the legend to Fig. S1.

RAB AND RAN GTPASES

The Rab (RAs-like in rat Brain) GTPases are the major regulators of vesicle-mediated membrane transport. They control the formation, transport and fusion of vesicles and organelles by recruiting tethering components to membranes during vesicle docking. Rab GTPases are typically modified with hydrophobic prenyl moieties at C-terminal cysteine residues, which anchor active GTP-bound Rab to membranes. Upon hydrolysis of GTP to GDP, Rabs bind to a GDP dissociation inhibitor (GDI), which masks the prenyl group and renders the Rab-GDI complex cytosolic and inactive. See (Lamber et al. 2019; Homma et al. 2020) for recent reviews.

A total of 72 Rab and Rab-like GTPases were detected in the *Ddis* genome, of which 39 were also present in *Dpur*, *Dlac*, *Ppal* and *Dfas* (Fig. S4). All genomes, but particularly *Ddis*, showed extensive gene amplification events of individual *Rab* genes. A subset of the Dictyostelid Rabs has been functionally analyzed. Cells overexpressing dominant negative and constitutively active Rab1A show defects in chemotaxis. The effects of Rab1A appear to be mediated by the Roco family member QkgA, with which Rab1A interacts (see Small GTPase families) (Kicka et al. 2011). Rab8A regulates tethering of the contractile vacuole to the plasma membrane, where it causes vacuole discharge after activation of GTP hydrolysis by its RabGAP Disgorgin (*dsgg*) (Du et al. 2008; Essid et al. 2012). The role of its close homolog Rab8B is less clear. Rab14 regulates both phagosome-lysosome fusion and contractile vacuole function and is negatively regulated by the RhoGAP/GEF protein DRG (XacA) discussed in the “Rho-Rac GTPases” section (Bush et al. 1996; Knetsch et al. 2001; Harris and Cardelli 2002). Rab2A and RabS localize to both Golgi membranes and contractile vacuoles and are required for the proper structure and function of both organelles (Maringer et al. 2016). Rab11A also regulates contractile vacuole function by recruiting its effector Drainin/PhgA, a RabGAP without GAP activity (Du et al. 2008), which is required for fusion of the contractile vacuole to the plasma membrane (Becker et al. 1999). Efficient vacuole fusion requires downregulation of Rab11A activity by the RabGAP CnrF, which is itself activated by vacuolar Ca^{2+} release through the P2XA ion channel (Parkinson et al. 2014). Ablation of the related Rab11B does not affect growth, endocytosis and development (Dragoi and OHalloran 1998). Rab21 is required for efficient phagocytosis and is regulated by two LIM-domain containing proteins (Khurana et al. 2005). The Rab GTPase ComB was detected in a screen for mutants that fail to secrete sporulation inducing signals, and may interact with the transporter ComD (Kibler et al. 2003). Rab7A is required for both phagocytosis and pinocytosis (Buczynski et al. 1997; Rupper et al. 2001).

Rab guanine nucleotide exchange factors

RabGEFs play a major role in both activating and localising Rabs at specific membrane compartments. Unlike the Arf, Rho and Ras GTPases, the RabGEFs do not share a common catalytic RabGEF domain. Many contain a longin/roadblock structural fold, which does not show much sequence similarity between RabGEFs, but forms an α-β-α sandwich that interacts with both small GTPases and membranes (Levine et al. 2013). Proteins with DENN (Differentially Expressed in Normal and Neoplastic cells) or VPS9 (Vacuolar Protein Sorting 9) domains make up the largest groups of putative RabGEFs (Ishida et al. 2016), while the other RabGEFs, are a very diverse set (see table S2 in (Homma et al. 2020)). RabGEF activity is also associated with the multisubunit TRAPP (TRAnsport Protein Particle) complexes that act as vesicle tethers (Kim et al. 2016).

To identify *Dictyostelium* RabGEFs, we isolated all VPS9 and DENN domain containing proteins and performed BLAST searches in Dictyostelid genomes using all other mammalian RabGEFs summarized by (Homma et al. 2020) as bait (Fig. S5). From the latter set, only four proteins were hit at sufficiently low E-values (≤ 5E-25) to qualify as homologs. The ~12 subunits of the deeply conserved TRAPP complexes are also present in Dictyostelia, but were not further analysed here. Of all putative *Ddis* RabGEFs, only the VPS9 protein RgfA was functionally analysed, with its overexpression causing delayed development and impaired chemotaxis to folate (Hadwiger 2013).

Rab GTPase activating proteins and guanine nucleotide dissociation inhibitors

Most RabGAPs contain a signature TBC (Tre-2/BUB2/cdc16) domain that harbours the GAP activity (Albert et al. 1999). Dictyostelia have 31 well conserved TBC domain proteins, but only four of those: Rbg3, Dsgg, PhgA and CnrF have been functionally analysed (Fig. S6). Rbg3 interacts with the heterotrimeric G-protein G2 and is required for normal lysosome function and growth and for *Ddis* life span regulation. Its GAP activity is essential for these roles. Rbg3 acts on Rab7A to mediate its effects on growth, but not lifespan regulation (Kuwayama et al. 2013). The roles of Dsgg, PhgA and CnrF were already discussed above.

Two deeply conserved RabGDI proteins are present in Dictyostelia (Fig. S6), but their roles have not been specifically studied in this organism.

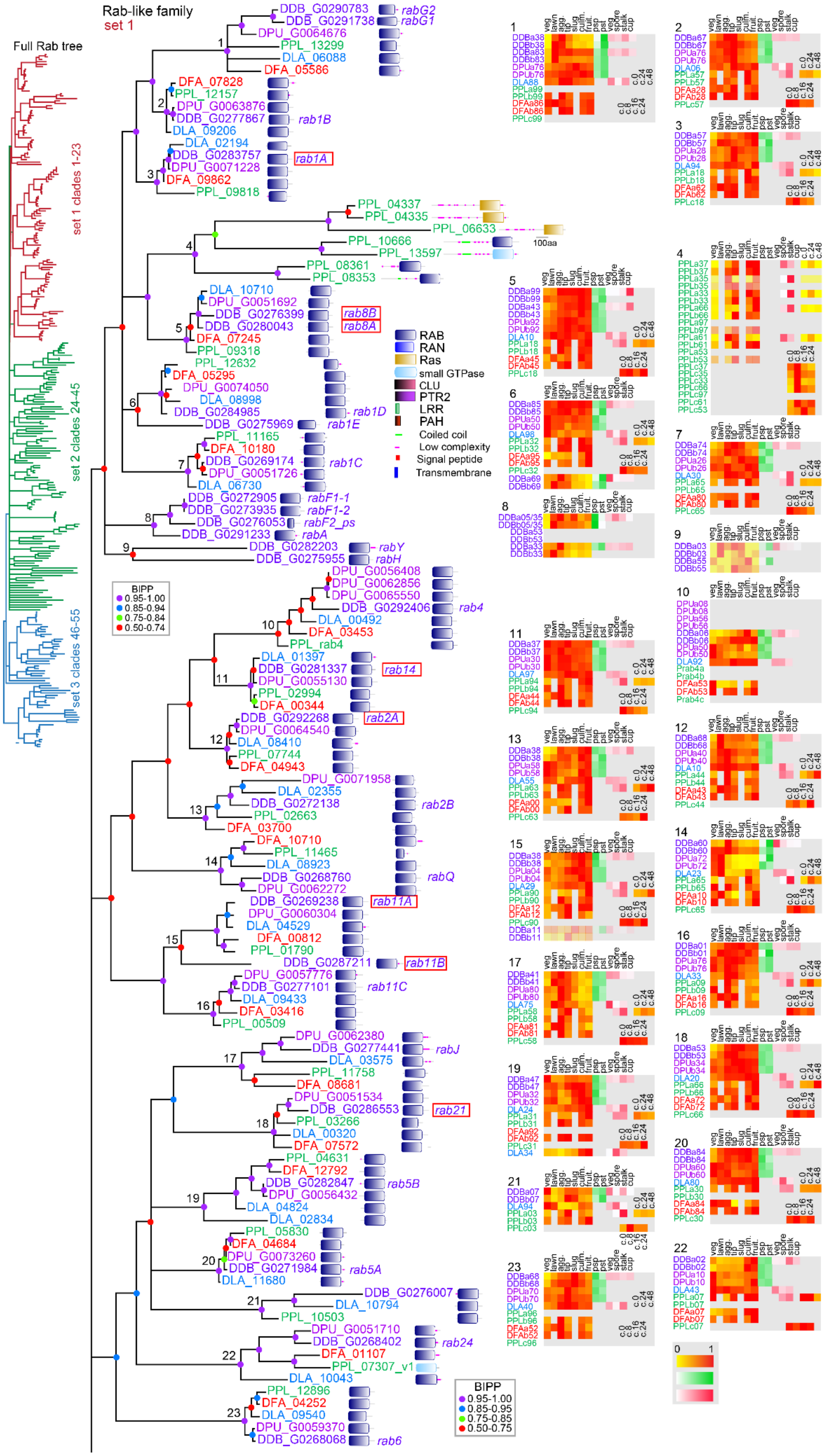
Rab-like family
set 1
Full Rab tree
set 1 clades 1-23
set 2 clades 24-45
set 3 clades 46-55
BIPP
0.95-1.00
0.85-0.94
0.75-0.84
0.50-0.74
RAB
RAN
Ras
small GTPase
CLU
PTR2
LRR
PAH
Coiled coil
Low complexity
Signal peptide
Transmembrane
100aa
rabG2
rabG1
rab1B
rab1A
rab8B
rab8A
rab1D
rab1E
rab1C
rabF1-1
rabF1-2
rabF2_ps
rabA
rabY
rabH
rab4
rab14
rab2A
rab2B
rabQ
rab11A
rab11B
rab11C
rabJ
rab21
rab5B
rab5A
rab24
rab6
veg
lawn
agg.
tip
slug
culm.
fruit.
psp
pst
spore
stalk
cup
c.0
c.8
c.16
c.24
c.48
0
1

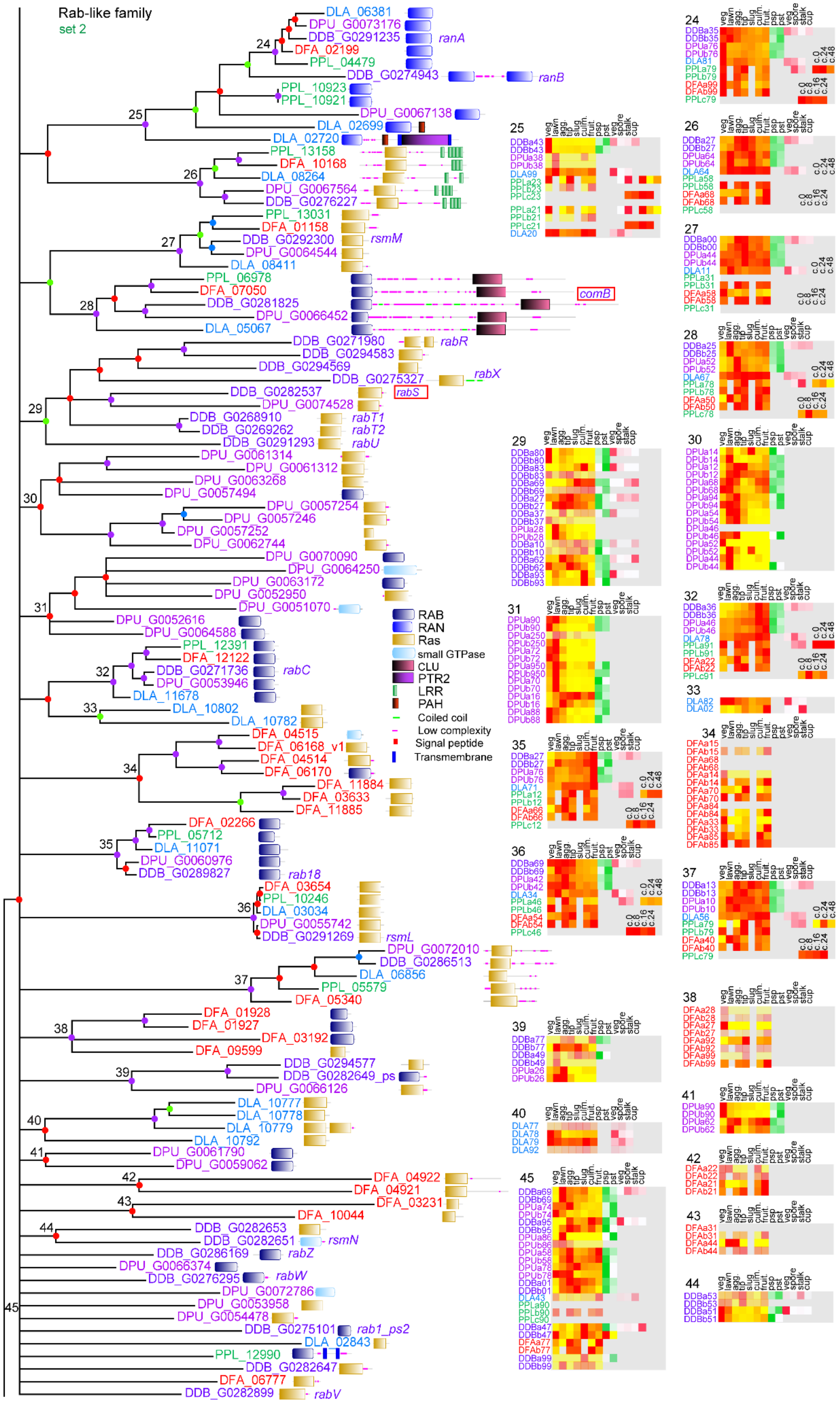

Rab-like family
set 2
DLA_06381
DPU_G0073176
DDB_G0291235
ranA
DFA_02199
PPL_04479
DDB_G0274943
ranB
PPL_10923
PPL_10921
DPU_G0067138
DLA_02699
DLA_02720
PPL_13158
DFA_10168
DLA_08264
DPU_G0067564
DDB_G0276227
PPL_13031
DFA_01158
DDB_G0292300
rsmM
DPU_G0064544
DLA_08411
PPL_06978
DFA_07050
DDB_G0281825
comB
DPU_G0066452
DLA_05067
DDB_G0271980
rabR
DDB_G0294583
DDB_G0294569
DDB_G0275327
rabX
DDB_G0282537
rabS
DPU_G0074528
DDB_G0268910
rabT1
DDB_G0269262
rabT2
DDB_G0291293
rabU
DPU_G0061314
DPU_G0061312
DPU_G0063268
DPU_G0057494
DPU_G0057254
DPU_G0057246
DPU_G0057252
DPU_G0062744
DPU_G0070090
DPU_G0064250
DPU_G0063172
DPU_G0052950
DPU_G0051070
DPU_G0052616
DPU_G0064588
PPL_12391
DFA_12122
DDB_G0271736
rabC
DPU_G0053946
DLA_11678
DLA_10802
DLA_10782
DFA_04515
DFA_06168_v1
DFA_04514
DFA_06170
DFA_11884
DFA_03633
DFA_11885
DFA_02266
PPL_05712
DLA_11071
DPU_G0060976
DDB_G0289827
rab18
DFA_03654
PPL_10246
DLA_03034
DPU_G0055742
DDB_G0291269
rsmL
DPU_G0072010
DDB_G0286513
DLA_06856
PPL_05579
DFA_05340
DFA_01928
DFA_01927
DFA_03192
DFA_09599
DDB_G0294577
DDB_G0282649_ps
DPU_G0066126
DLA_10777
DLA_10778
DLA_10779
DLA_10792
DPU_G0061790
DPU_G0059062
DFA_04922
DFA_04921
DFA_03231
DFA_10044
DDB_G0282653
DDB_G0282651
rsmN
DDB_G0286169
rabZ
DPU_G0066374
DDB_G0276295
rabW
DPU_G0072786
DPU_G0053958
DPU_G0054478
DDB_G0275101
rab1_ps2
DLA_02843
PPL_12990
DDB_G0282647
DFA_06777
DDB_G0282899
rabV
RAB
RAN
Ras
small GTPase
CLU
PTR2
LRR
PAH
Coiled coil
Low complexity
Signal peptide
Transmembrane
veg
lawn
agg.
tip
slug
culm.
fruit.
psp
pst
veg
spore
stalk
cup
c.0
c.8
c.16
c.24
c.48
24
25
26
27
28
29
30
31
32
33
34
35
36
37
38
39
40
41
42
43
44
45

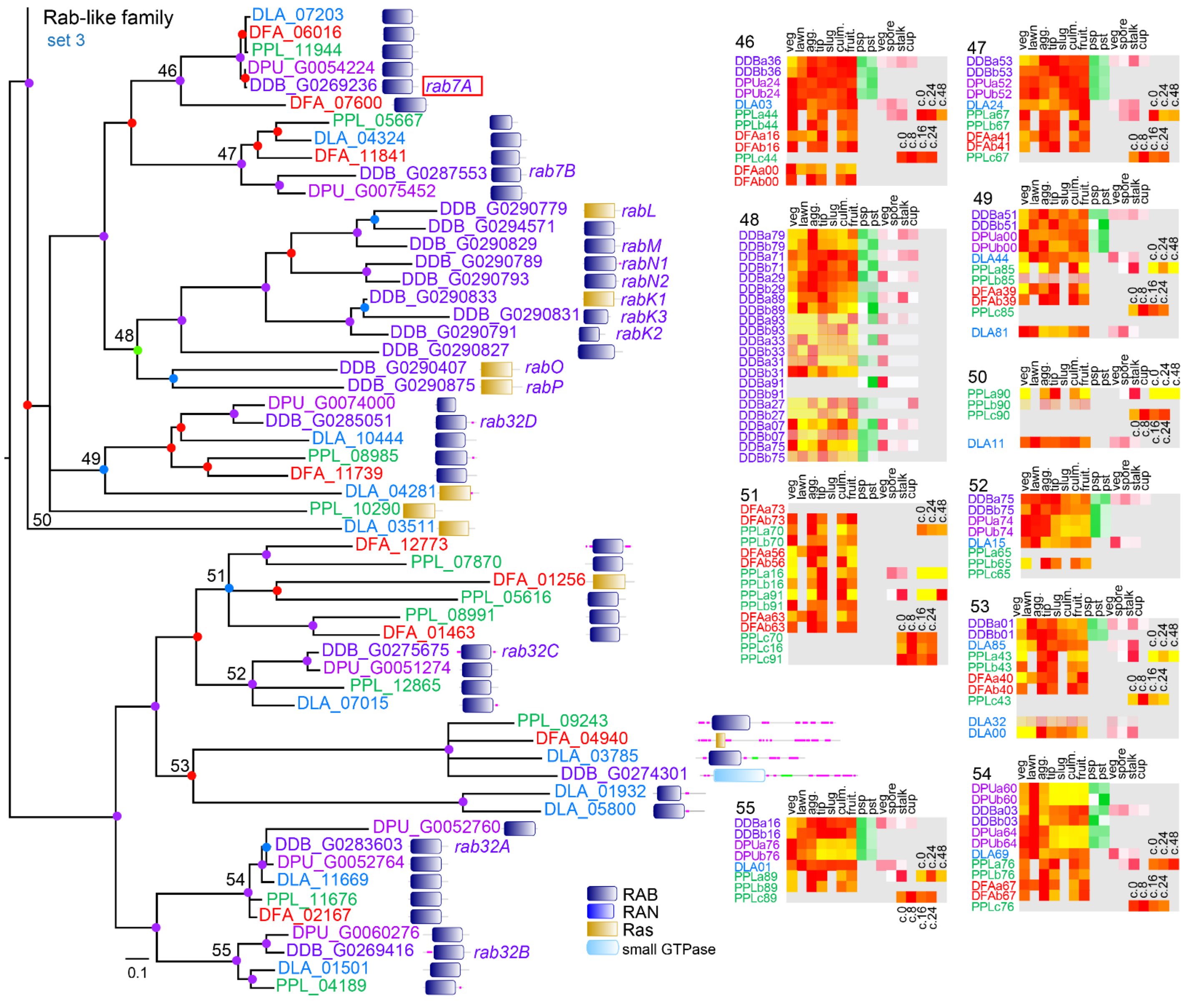

Figure S4. Conservation and change in the Rab-like GTPases across Dictyostelia.
GTPases in the *Ddis*, *Dpur*, *Dlac*, *Ppal* and Dfas genomes were identified from Interpro scans as outlined in Methods and a pilot phylogenetic tree was prepared from all aligned sequences. In this tree the Rab and Ran GTPases formed a single clade. The sequences in this clade were further supplemented by Blast queries using Rab and Ran sequences as bait. A final tree was then constructed (top left, set 1), which due to its size had to be presented in three parts. The tree was annotated with protein functional domains and heatmaps of developmental and cell-type specific expression of the genes as outlined in Methods and the legend to Fig. S1.

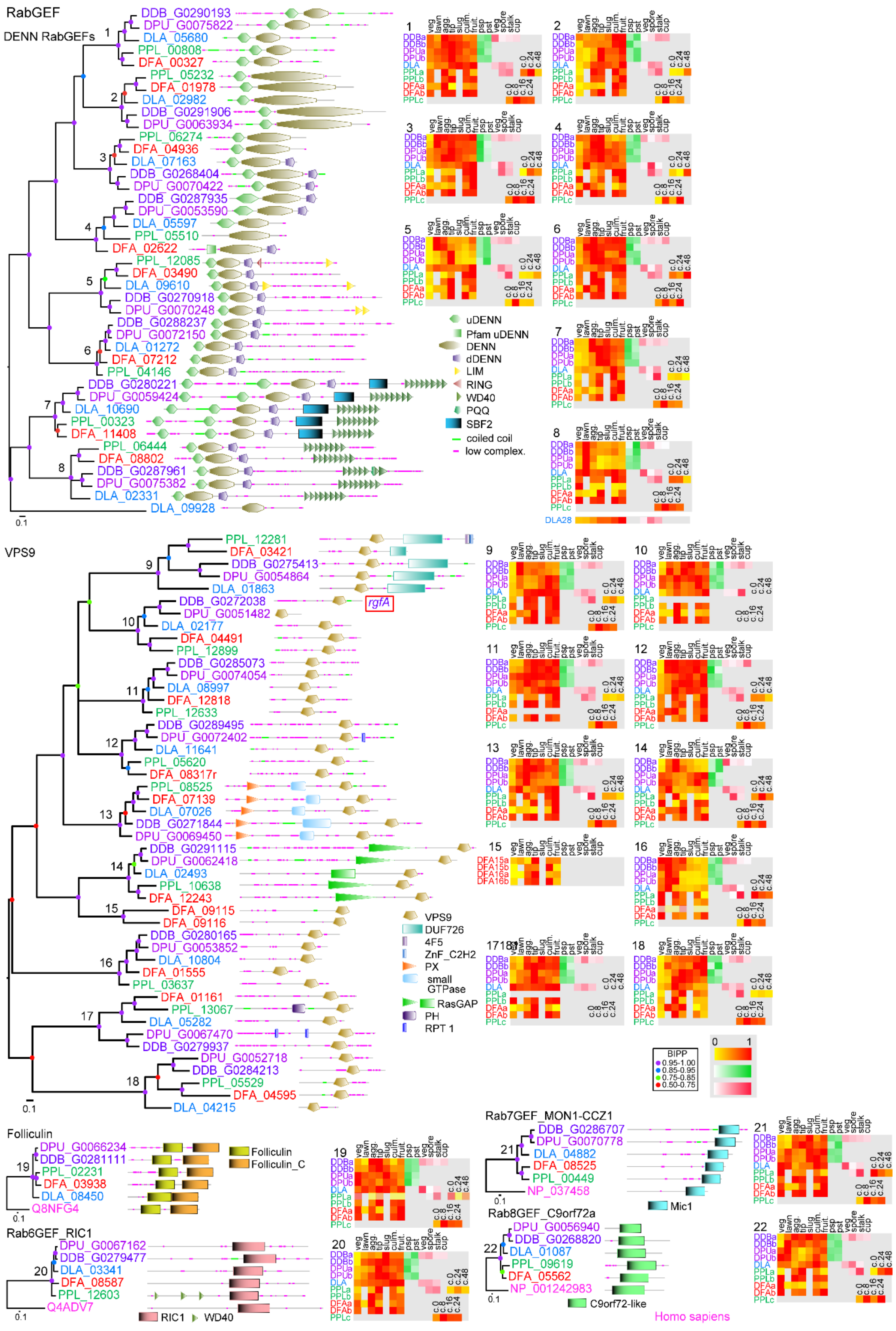
RabGEF
DENN RabGEFs
DDB_G0290193
DPU_G0075822
DLA_05680
PPL_00808
DFA_00327
PPL_05232
DFA_01978
DLA_02982
DDB_G0291906
DPU_G0063934
PPL_06274
DFA_04936
DLA_07163
DDB_G0268404
DPU_G0070422
DDB_G0287935
DPU_G0053590
DLA_05597
PPL_05510
DFA_02622
PPL_12085
DFA_03490
DLA_09610
DDB_G0270918
DPU_G0070248
DDB_G0288237
DPU_G0072150
DLA_01272
DFA_07212
PPL_04146
DDB_G0280221
DPU_G0059424
DLA_10690
PPL_00323
DFA_11408
PPL_06444
DFA_08802
DDB_G0287961
DPU_G0075382
DLA_02331
DLA_09928
uDENN
Pfam uDENN
DENN
dDENN
LIM
RING
WD40
PQQ
SBF2
coiled coil
low complex.
veg lawn agg. tip slug culm. fruit. psp pst veg spore stalk cup
DDBa DDBb DPUa DPUb DLA PPLa PPLb DFAa DFAb PPLc
c.0 c.24 c.48
c.0 c.8 c.16 c.24
DLA28
VPS9
PPL_12281
DFA_03421
DDB_G0275413
DPU_G0054864
DLA_01863
DDB_G0272038
DPU_G0051482
rgfA
DLA_02177
DFA_04491
PPL_12899
DDB_G0285073
DPU_G0074054
DLA_08997
DFA_12818
PPL_12633
DDB_G0289495
DPU_G0072402
DLA_11641
PPL_05620
DFA_08317r
PPL_08525
DFA_07139
DLA_07026
DDB_G0271844
DPU_G0069450
DDB_G0291115
DPU_G0062418
DLA_02493
PPL_10638
DFA_12243
DFA_09115
DFA_09116
DDB_G0280165
DPU_G0053852
DLA_10804
DFA_01555
PPL_03637
DFA_01161
PPL_13067
DLA_05282
DPU_G0067470
DDB_G0279937
DPU_G0052718
DDB_G0284213
PPL_05529
DFA_04595
DLA_04215
VPS9
DUF726
4F5
ZnF_C2H2
PX
small GTPase
RasGAP
PH
RPT 1
DFA15a DFA15b DFA16a DFA16b
BIPP
0.95-1.00
0.85-0.95
0.75-0.85
0.50-0.75
Folliculin
DPU_G0066234
DDB_G0281111
PPL_02231
DFA_03938
DLA_08450
Q8NFG4
Folliculin
Folliculin_C
Rab6GEF_RIC1
DPU_G0067162
DDB_G0279477
DLA_03341
DFA_08587
PPL_12603
Q4ADV7
RIC1
WD40
Rab7GEF_MON1-CCZ1
DDB_G0286707
DPU_G0070778
DLA_04882
DFA_08525
PPL_00449
NP_037458
Mic1
Rab8GEF_C9orf72a
DPU_G0056940
DDB_G0268820
DLA_01087
PPL_09619
DFA_05562
NP_001242983
C9orf72-like
Homo sapiens

Figure S5. Rab Guanine Nucleotide Exchange Factors

The RabGEFs are a very diverse set, with the largest groups using the DENN or VPS9 domains for guanine nucleotide exchange. The DENN and VPS9 proteins were identified from genome Interpro scans by the presence of the Interpro ID IPR001194 and IPR003123 domains, respectively, and phylogenetic trees were inferred. The other RabGEFs were identified by BlastP searches using all human RabGEF sequences listed in Table S1 of (Homma et al. 2020). Spurious hits with E-values > 0.001 were ignored. The human sequences are incorporated in the phylogenetic trees with their likely Dictyostelid orthologs. All trees were annotated with protein functional domains and heatmaps of developmental and cell-type specific expression of the genes as outlined in Methods and the legend to Fig. S1.

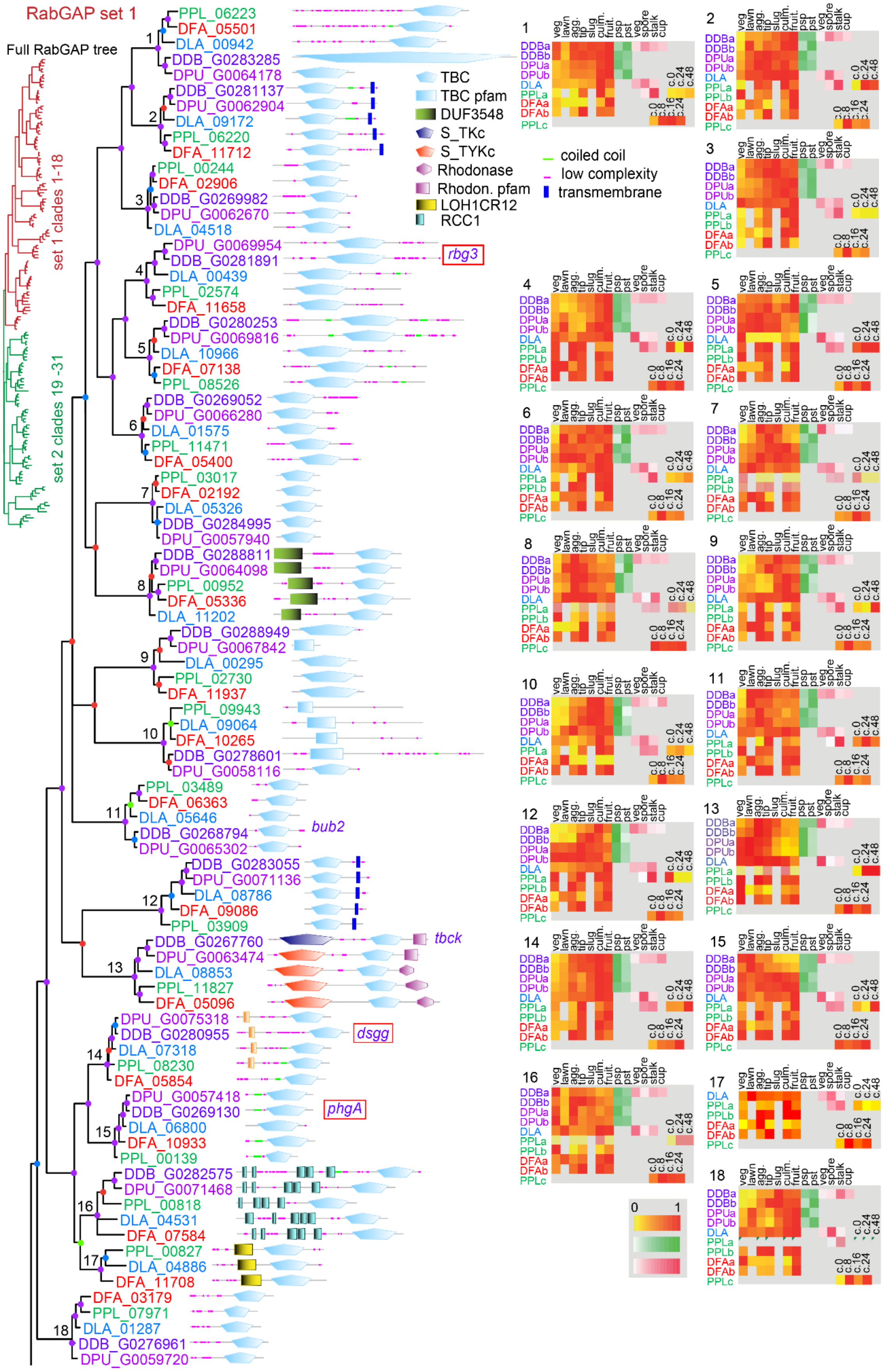
RabGAP set 1
Full RabGAP tree
set 1 clades 1-18
set 2 clades 19 -31
PPL_06223
DFA_05501
DLA_00942
DDB_G0283285
DPU_G0064178
DDB_G0281137
DPU_G0062904
DLA_09172
PPL_06220
DFA_11712
PPL_00244
DFA_02906
DDB_G0269982
DPU_G0062670
DLA_04518
DPU_G0069954
DDB_G0281891
DLA_00439
PPL_02574
DFA_11658
DDB_G0280253
DPU_G0069816
DLA_10966
DFA_07138
PPL_08526
DDB_G0269052
DPU_G0066280
DLA_01575
PPL_11471
DFA_05400
PPL_03017
DFA_02192
DLA_05326
DDB_G0284995
DPU_G0057940
DDB_G0288811
DPU_G0064098
PPL_00952
DFA_05336
DLA_11202
DDB_G0288949
DPU_G0067842
DLA_00295
PPL_02730
DFA_11937
PPL_09943
DLA_09064
DFA_10265
DDB_G0278601
DPU_G0058116
PPL_03489
DFA_06363
DLA_05646
DDB_G0268794
DPU_G0065302
DDB_G0283055
DPU_G0071136
DLA_08786
DFA_09086
PPL_03909
DDB_G0267760
DPU_G0063474
DLA_08853
PPL_11827
DFA_05096
DPU_G0075318
DDB_G0280955
DLA_07318
PPL_08230
DFA_05854
DPU_G0057418
DDB_G0269130
DLA_06800
DFA_10933
PPL_00139
DDB_G0282575
DPU_G0071468
PPL_00818
DLA_04531
DFA_07584
PPL_00827
DLA_04886
DFA_11708
DFA_03179
PPL_07971
DLA_01287
DDB_G0276961
DPU_G0059720
rbg3
bub2
tbck
dsgg
phgA
TBC
TBC pfam
DUF3548
S_TKc
S_TYKc
Rhodonase
Rhodon. pfam
LOH1CR12
RCC1
coiled coil
low complexity
transmembrane
veg lawn agg. tip slug culm. fruit. psp pst veg spore stalk cup
DDBa DDBb DPUa DPUb DLA PPLa PPLb DFAa DFAb PPLc
c.0 c.24 c.48
c.0 c.8 c.16 c.24
0 1

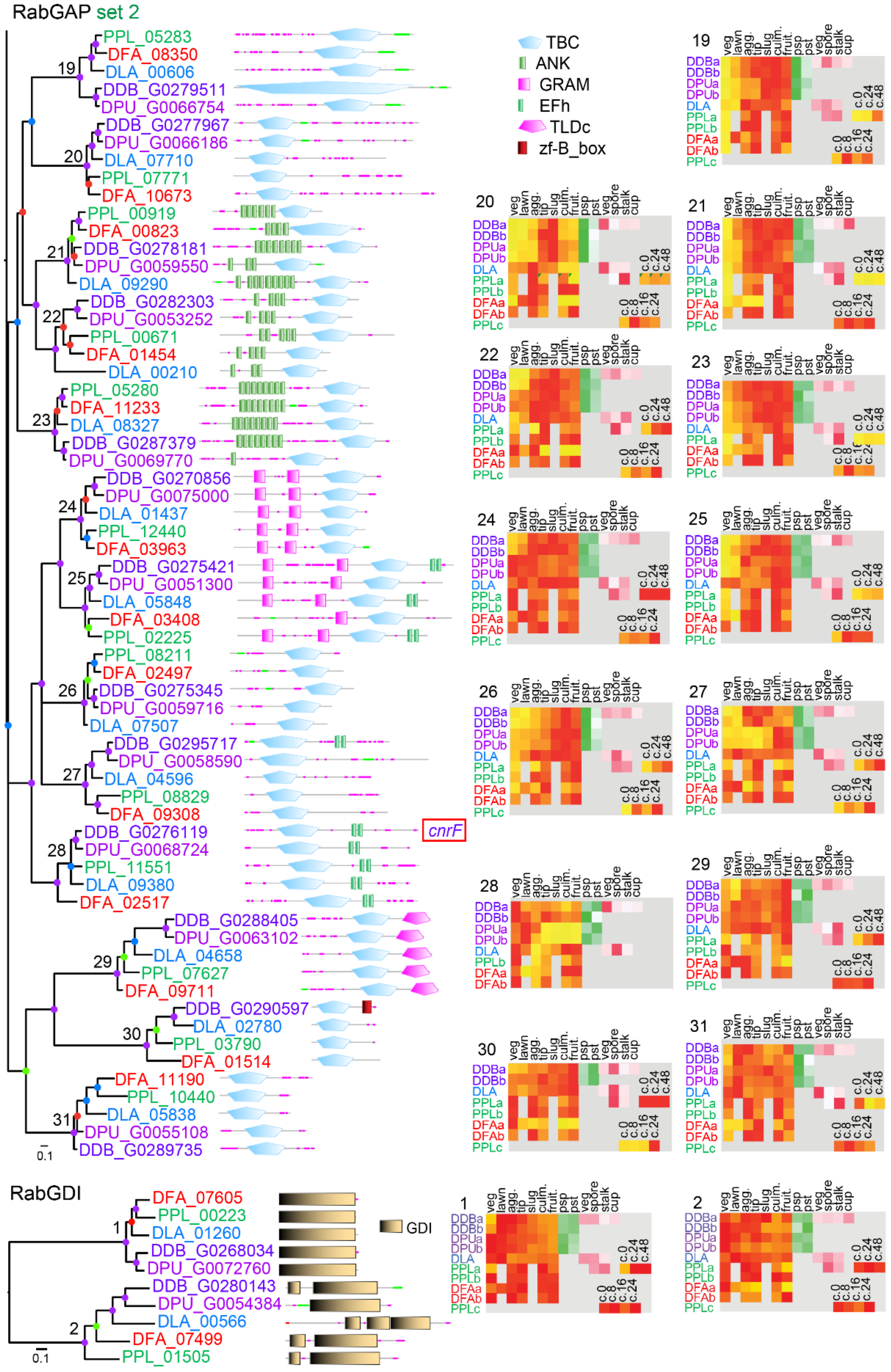

Figure S6. Rab GTPase Activating Proteins and GDP Dissociation Inhibitors

Sequences with the RabGAP TBC domain or RabGDI domains were retrieved from five Dictyostelid genomes with Interpro identifiers IPR000195 and IPR000806, respectively, and by BLAST searches as detailed in Methods. Phylogenetic trees were inferred from the isolated TBC domain sequences and full GDI proteins, and annotated with protein functional domains and heatmaps of developmental and cell-type specific expression of the genes as outlined in Methods and the legend to Fig. S1.

Ran GTPases

The two *Ddis* Ran (Ras-related nuclear protein) GTPases, RanA and RanB, group in our analysis with the Rab GTPases (Fig. S4, set 2, clade 25). Ran GTPases regulate transport of molecules from the cytoplasm to the nucleus and *vice versa* through the nuclear pore complex. They also act on cell cycle progression by regulating mitotic spindle formation (Boudhraa et al. 2020) and are essential for nuclear envelope formation. Ran GTPases differ from most other GTPases in lacking C-terminal hydrophobic modifications for membrane insertion. Instead, they harbour a C-terminal acidic motif (DEDDDL). This motif moves away from the GTP binding domain upon GTP binding to interact with a range of Ran effectors. Ran is activated by an unusual RanGEF with RCC1 domains (Regulator of Chromosome Condensation 1), which resides in the nucleus, and inactivated by RanGAP1, which is cytosolic. This results in a gradient of activated Ran from the nucleus to the cytosol (Matchett et al. 2014). The activity of RanGAP1 requires the accessory protein RanBP1, which is proposed to relieve inhibition of RanGAP1 by the acidic terminus of Ran (Seewald et al. 2002).

Ran guanine nucleotide exchange factors

The RCC1 domains of human RanGEF form a seven-blade beta-propeller structure, which binds to chromatin and interacts with Ran (Renault et al. 1998). However, one or more RCC1 repeats are also found in many unrelated proteins, where they inhibit enzyme activity or act as protein or lipid interaction domains (Hadjebi et al. 2008). *Ddis* contains 24 proteins with RCC1 repeats, of which 23 are conserved throughout Dictyostelia (Fig. S7). BLASTp query of Dictyostelid genomes with human RCC1 yields different members of clades 1, 8 and 9 as top hits, indicating that human RCC1 is not particularly similar to either of these proteins. The clade 8 and 9 proteins are structurally dissimilar from RCC1 with long N-terminal extensions and/or additional domains, while the clade 1, 2, 3, 5, 14 and 24 proteins consist only of a single cluster of RCC1 repeats, like human RCC1. Further evidence is required to identify either of these proteins as a RanGEF. Disruption of DDB_G0285261 results in aberrant cAMP signalling and streaming (Sawai et al. 2008). Two characterized *Ddis* RCC1 proteins, RgbA and GefC also have GAP and GEF domains for the Rho-Rac and Ras families and will be discussed in the next sections. Dd5P2 additionally harbours an inositol-5 phosphatase domain and mildly affects actin polymerization (Loovers et al. 2006). TirA, with an additional Toll/Interleukin1-Receptor domain, mediates *Ddis* resistance to bacterial infections (Zhang et al. 2016).

Ran GTPase activating proteins

The *Dictyostelium* RanGAP is also elusive. Human RanGAP1 consist of LRR repeats that are followed by a RanGAP1_C domain. Yeast RanGAP only consists of the LRR repeats, which harbour the GTPase activating activity (Seewald et al. 2002). *Dictyostelium* has many proteins with LRR repeats, but none was particularly related to either the yeast or human RanGAP1 (personal observation). The RanGAP1_C domain (IPR009109) is required for RanGAP1 localization at the nuclear pore complex of vertebrates, but is not present in the dictyostelid genomes.

Dictyostelids do have two well conserved Ran binding proteins (Fig. S7), indicating that RanGEF and RanGAP are also likely to be present. Upon *Ddis* infection with *Legionella* bacteria, RanA and RanBP1 localise to the *Legionella* containing vacuole. Here RanA is activated by the *Legionella* RCC1 repeat containing protein LegG1. Activated Ran then stabilises microtubuli to promote vacuole motility (Hilbi et al. 2014; Swart et al. 2020).

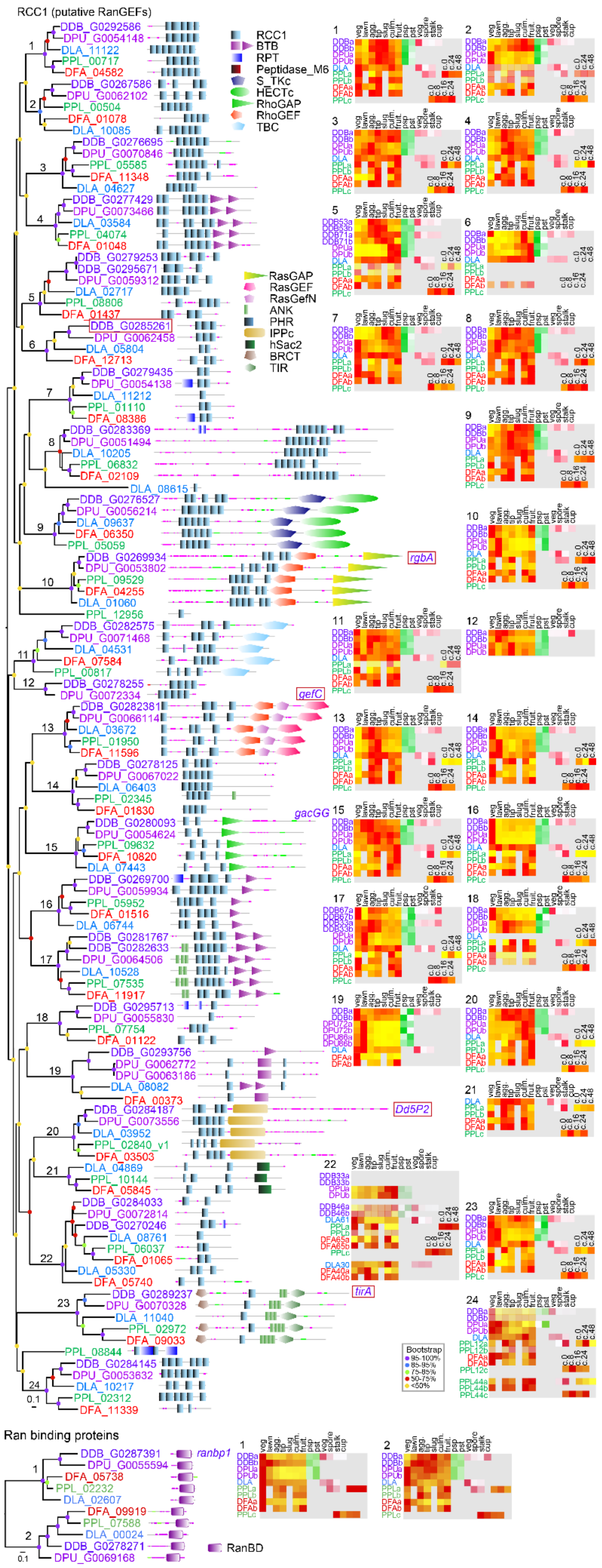

Figure S7. Putative Ran regulators

The guanine nucleotide exchange activity of human RanGEF resides in its cluster of RCC1 (Regulator of Chromosome Condensation 1) domains (Renault et al. 1998). Proteins with RCC1 domains and Ran binding proteins were isolated from Interpro scans of dictyostelid genomes by their identifier IPR009091 and IPR000156, respectively. Sequences were aligned with Clustal Omega and phylogeny were inferred with MrBayes for Ran binding proteins and RAXML (Stamatakis 2014) for RCC1 proteins, which yielded better resolution of the deeper nodes than MrBayes. The trees were annotated with protein domain architectures and transcription profiles as described in the legend to Fig. S1.

RHO-RAC GTPASES

The Rho (Ras homolog) GTPases were initially identified as key regulators of the actin cytoskeleton dynamics. Apart from being implicated in most actin-regulated processes such as vesicle trafficking, cell motility, cytokinesis, adhesion, and morphogenesis, they also regulate processes not directly linked to actin, like NADPH oxidase activation, microtubule organisation, gene expression, cell cycle progression, apoptosis and tumorigenesis (Ueyama 2019). A signature of Rho GTPases is a short insert rich in charged residues, which contributes to specificity of functions against GTPases of other families. Most Rho GTPases also carry a C-terminal CAAX motif, a signal for attachment of an isoprenyl modification, immediately preceded by a polybasic stretch rich in lysine residues. These C-terminal features are important for association to membranes and interaction with Rho guanine nucleotide dissociation inhibitors.

Ddis has 22 Rho GTPases, of which 12 are conserved throughout Dictyostelia (Fig. S8). *Ddis* Rac1a, Rac1b and Rac1c are the result of gene amplifications within clade 7. Similar to other small GTPase families, gene amplification events outside the conserved clades can be observed in all species of Dictyostelia. In addition, all Dictyostelia harbor a divergent atypical Rho GTPase with an N-terminal extension that contains several leucine-rich repeats (clade15). Historically all *Ddis* Rho GTPases have been named Rac, although only a small subset can be assigned to the Rac subfamily of other eukaryotes and no clear RhoA and Cdc42 orthologs emerge based on sequence comparisons (Rivero et al. 2001). RacA is a member of the RhoBTB subfamily of atypical Rho GTPases with links to the ubiquitin dependent degradation machinery.

Functional data exists for a subset of *Ddis* Rac genes (see (Rivero and Xiong 2016) for references). The closely related Rac1a, Rac1b and Rac1c have been relatively widely studied. They are functionally redundant but collectively essential and have roles in cell morphology, motility, chemotaxis, cytokinesis and endocytosis. Disruption of *racA* results in impaired growth with normal pinocytosis and cytokinesis. RacB has been implicated in the regulation of chemotaxis and can be activated by RacGEF1 and a Dock/Elmo complex containing ElmoE. Disruption of *racC* causes a cytokinesis defect and impairs chemotaxis. RacE is essential for cytokinesis by modulating the distribution of cytoskeletal components that ensure cortical stiffness. RacE is also implicated in chemotaxis, where it appears to be important for positioning the directional sensing machinery at the leading front. RacF1 and RacF2 are very closely related and seem to play redundant roles in sexual cell fusion. RacG participates in cell morphology, chemotaxis and endocytosis. RacH associates with ER and Golgi membranes and is specifically implicated in vesicle trafficking. Cells lacking RacH are more susceptible to intracellular proliferation of *Mycobacterium* and *Legionella* due to defective acidification of the proliferative vacuole. Disruption of *racI*, *racJ* and *racL* did not result in growth or development defects, but their participation in other processes has not been addressed.

Rho guanine nucleotide exchange factors

Two distinct classes of RhoGEFs have been described, each characterized by an unrelated short domain that displays nucleotide exchange activity. These classes are the conventional RhoGEFs, featuring a ~180 residues DH (Diffuse B-cell lymphoma Homology) domain and the CZH family, characterized by a ~400 residues CZH2 (CDM-zizimin homology) domain 2, also known as DOCKER or DHR2, Dock (Dedicator Of CytoKinesis) homology region 2 (Laurin and Côté 2014; Fort and Blangy 2017). Dock proteins require the interaction with scaffold proteins of the Elmo (EnguLfment and cell MOtility) family for localization and GEF activity (Xu and Jin 2019). Both DH and Dock-Elmo families are present in Dictyostelids.

Ddis contains 46 DH RhoGEFs distributed in 44 well defined clades conserved throughout Dictyostelia (two *Ddis* RhoGEFs exist each as two variants in clades 3 and 38) (Fig. S9). The DH domain is commonly followed by a pleckstrin-homology (PH) domain that is thought to regulate the exchange activity of the DH domain and acts as a membrane anchor. The PH domain is consistently missing in 6 clades: GefC, GxcTT, RgbA, GxcE, GxcAA and GxcF. Many DH RhoGEFs have been characterized functionally to some extent. GxcA (RacGEF1), a GEF for RacB and Rac1, is responsible for activation of about half of the RacB required for chemotaxis and also binds activated Rap1, which may affect its GEF activity. Nevertheless, *gxcA*$^-$ cells display unimpaired chemotaxis and development (Park et al. 2004; Lee and Jeon 2012). GefC (RasGEFC) carries a RasGEF domain and several RCC1 domains and is commented on in the "Ras and Rap GTPases" section. Roco5 belongs to the Roco family and is mentioned in the "Small families of GTPases" section. GxcT is a potential GEF for RacE and *gxcT*$^-$ cells phenocopy the cytokinesis defect of *racE*$^-$ cells in addition to showing a chemotaxis defect (Wang et al. 2013). Two genes, *gxcJJ* and *gxcKK*, were identified in mutagenesis screens as disrupted in mutants that displaying aberrant cAMP wave formation or decreased socially cooperative development, respectively (Santorelli et al. 2008; Sawai et al. 2008). GxcU is related to human frabin and binds the RhoGAP

Dd5P4 (Luscher et al. 2019). RgbA (RGBARG) contains RCC1, RhoGEF and RasGAP domains. By interacting with RacG and RacH and regulating numerous Ras GTPases, this protein integrates Ras and Rac signaling to allow efficient macropinosome and phagosome formation (Buckley et al. 2020). Three proteins (XacA, XacB, XacC) combine RhoGEF with RhoGAP domains and are discussed with the RhoGAPs below. GxcB (TRIX) appears to be involved in late steps of the endocytosis pathway (Strehle et al. 2006). GxcC binds RacG and RacH among GTPases, and its N-terminus binds activated RapA. *gxcC*⁻ cells displayed a delayed development phenotype with chemotaxis, adhesion, and morphology being unaffected (Plak et al. 2013). GxcDD features an ArfGAP domain and has been discussed in the “Arf-Sar GTPases” section above. MyoM is an unconventional myosin with a DH-PH combination at its tail that displays GEF activity on Rac1. The protein accumulates at the cell cortex and macropinosomes, but *myoM*⁻ cells are not impaired in growth, phagocytosis and development (Geissler et al. 2000). In addition to the RhoGEFs described above, 14 additional RhoGEFs were disrupted (Wang et al. 2013); none of the knockout strains was noticeably impaired in growth and development and they have not been investigated further.

The evolutionarily distinct and smaller family of Dock proteins (Fig. S10) is represented in all Dictyostelids by 8 conserved genes equally distributed into two well defined subfamilies, Dock proper (*dockA-D*) and Zizimin (*zizA-D*). Members of the former contain an N-terminal SH3 domain and C-terminal proline-rich motifs that can mediate intra- or intermolecular binding to SH3 domains, however an SH3 domain is absent in DockC. Functional data are available for 4 Dock genes. Whereas ablation of *zizA* caused no obvious phenotype, ablation of *zizB*, *dockA* and *dockD* suggests variable roles in development and chemotaxis (Pakes et al. 2013). Regarding Elmo, the *Ddis* genome harbours 6 genes that are all conserved in Dictyostelids (Fig. S10). Two have been functionally analysed. ElmoA, acting in a complex with DockD, negatively regulates actin polymerization and appears to be involved in the coordination of phagocytosis and cell migration (Isik et al. 2008; Para et al. 2009). ElmoE forms complexes with DockC and ZizA and interacts with the Gβ subunit of heterotrimeric G proteins to transduce the cAMP receptor signal to the cytoskeleton by activating RacB (Yan et al. 2012).

Rho GTPase activating proteins

The signature of RhoGAPs is a ~150 residue domain, known as the breakpoint cluster region-homology domain, with a characteristic fold and a highly conserved arginine residue important for catalytic activity. The *Ddis* genome harbors 47 genes encoding RhoGAPs (Fig. S11) that are conserved in all or most Dictyostelids (*gacII* is a partial duplication of *xacA* in clade 35, that occurred only in *Ddis*) and members of one clade (48) are absent in *Ddis* and *Dpur*. Notably, the RhoGAP domains of the *Ddis* and *Dpur* Pats1 (clade 37) are vestigial and therefore not identifiable in the domain architecture analyses. Only a small subset of RhoGAPs have been studied functionally. The most salient role of MEGAP (MEntal retardation GAP) proteins (clades 2-5) is to maintain the morphology and functionality of the contractile vacuole, as demonstrated in mutants in which *mgp1*, *mgp2* or both were disrupted (Heath and Insall 2008). GacG is a PkbA/PkbR1 substrate required for efficient micropinocytosis (Williams et al. 2019b). Cells deficient in *gacT* show alterations in various stages of the development cycle (Sawai et al. 2008), and cells deficient in *gacH* display deficient chemotaxis to cAMP and aggregate formation (Nichols et al. 2019).

Dd5P4 is a homolog of human OCRL (OculoCerebRorenal syndrome of Lowe) and features a 5-phosphatase domain that acts on plasma membrane phosphoinositides. Dd5P4 interacts with the RhoGEF GxcU for proper endocytosis and contractile vacuole function. *Dd5P4*⁻ mutants also exhibit increased intracellular replication of *Legionella* as well as random motility due to increased Ras activity at the cell cortex (Loovers et al. 2003; Weber et al. 2009; Li et al. 2018; Luscher et al. 2019). GefD and GflB, 2 out of 3 proteins that combine a RasGEF domain with a RhoGAP domain, have been functionally analyzed and are described in the “Ras-Rap” section. Three RhoGAPs, Roco9, Roco10 and Pats1, belong to the Roco family of small GTPases and are described in the “Small families of GTPases” section.

Out of three proteins that combine RhoGAP and RhoGEF domains (XacA, XacB and XacC), XacA (DdRacGAP1, DRG) is well characterized. Its N-terminal RhoGAP domain has GAP activity on RabD and Rac1 and the C-terminal RhoGEF domains have GEF activity on Rac1. The protein plays dual roles in chemotaxis and actin organization on one hand, and in the regulation of the contractile vacuole system on the other (Knetsch et al. 2001). Knockout strains for *xacB* and *xacC* were not found noticeably impaired in growth and development and have not been investigated further (Wang et al. 2013).

Rho guanine nucleotide dissociation inhibitor

RhoGDI has complex roles in Rho GTPase signaling regulation. It extracts Rho from membranes by interacting with the isoprenyl moiety, maintaining a cytosolic pool and protecting the GTPase from degradation.

RhoGDI inhibits the release of the nucleotide and the GTPase activity of Rho (Garcia-Mata et al. 2011). Two members of this family have been described in *Ddis*, RhoGDI1 (*rdiA)* and RhoGDI2 (*rdiB)*, of which only *rdiA* is conserved in Dictyostelia (Fig. S11). RhoGDI1 interacts with a subset of Rho GTPases (Rac1, RacB, RacC and RacE). *rdiA*$^{-}$ cells show a mild phenotype that includes aberrant cytokinesis, decreased actin polymerization response and enlarged contractile vacuole system (Rivero et al. 2002). RhoGDI2 is unusually short and does not interact with Rho GTPases. It is expressed at late stages of development, but its ablation does not disrupt development (unpublished observations).

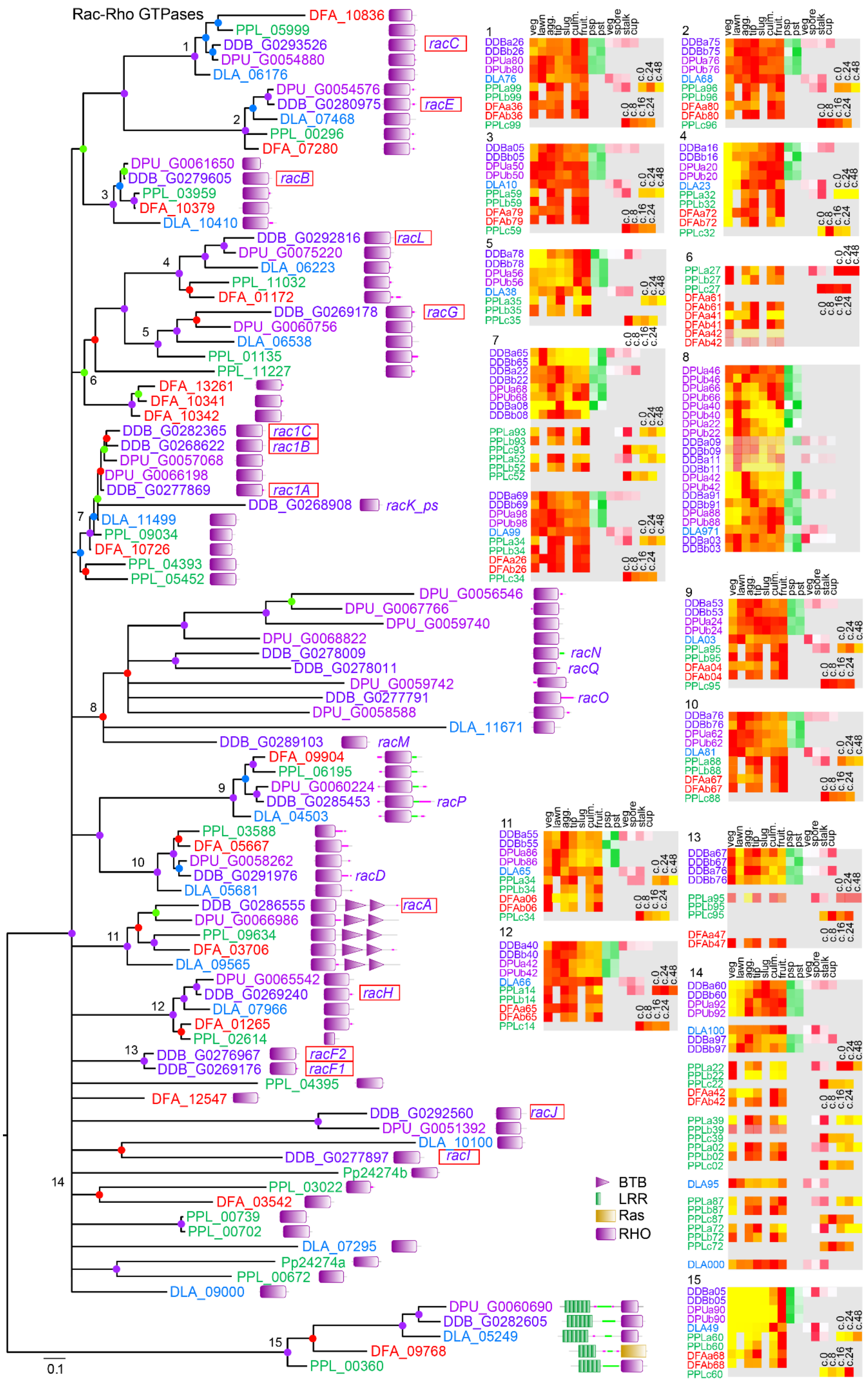

Figure S8. Conservation and change of Rac-Rho GTPases across Dictyostelia

The Rac-Rho GTP-ases were identifed as a separate clade in a pilot tree of all Dictyostelid GTPases, see Methods. The sequences in this clade were further supplemented with hits of BLAST queries of Dictyostelid genomes using Rac-Rho sequences as bait. A final tree was then inferred with was annotated with protein domain architectures and gene expression profiles, as outlined in the legend to Fig. S1.

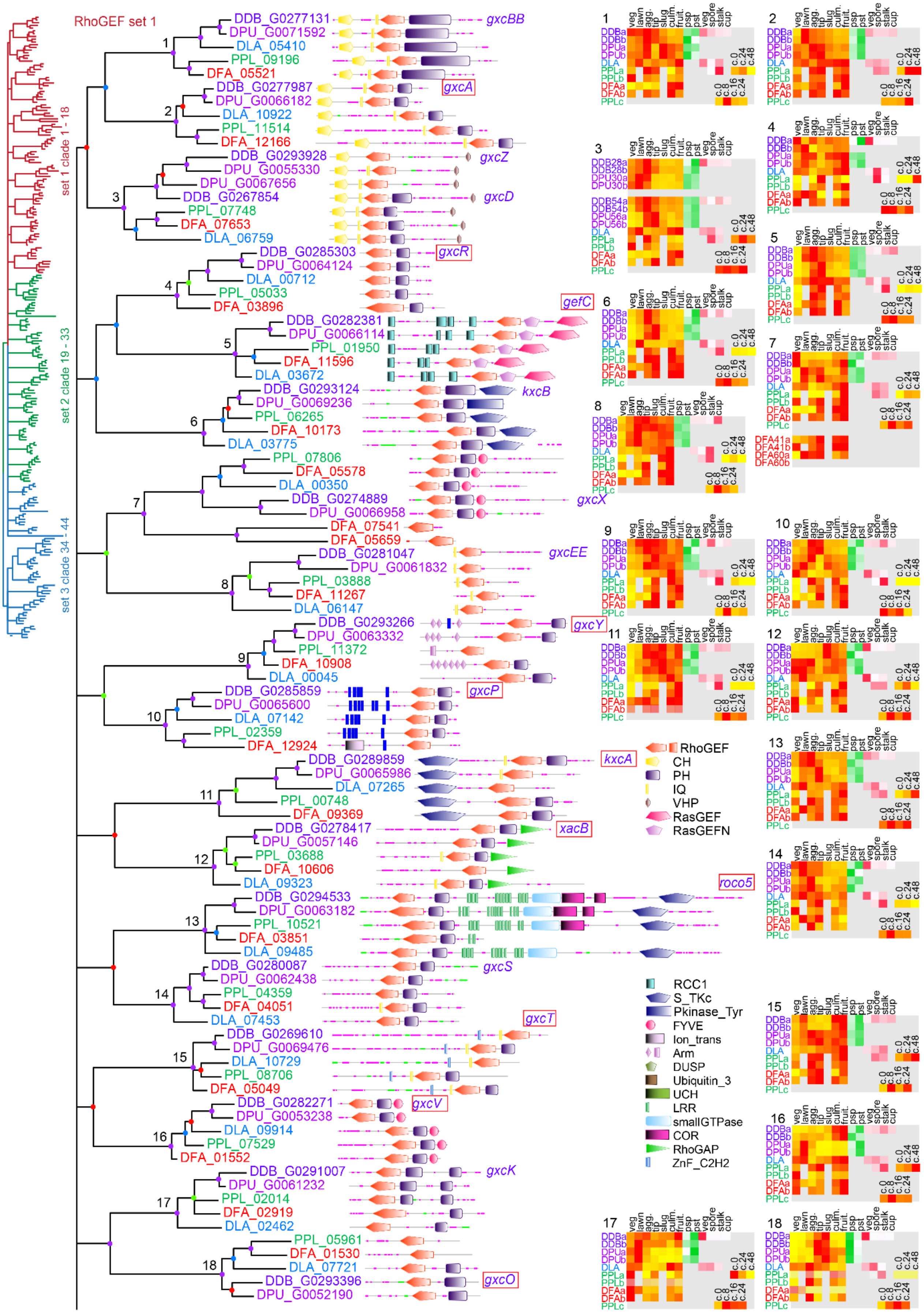

RhoGEF set 1
set 1 clade 1 - 18
set 2 clade 19 - 33
set 3 clade 34 - 44
1
DDB_G0277131
DPU_G0071592
DLA_05410
PPL_09196
DFA_05521
gxcBB
2
DDB_G0277987
DPU_G0066182
DLA_10922
PPL_11514
DFA_12166
gxcA
3
DDB_G0293928
DPU_G0055330
DPU_G0067656
DDB_G0267854
PPL_07748
DFA_07653
DLA_06759
gxcZ
gxcD
4
DDB_G0285303
DPU_G0064124
DLA_00712
PPL_05033
DFA_03896
gxcR
5
DDB_G0282381
DPU_G0066114
PPL_01950
DFA_11596
DLA_03672
gefC
6
DDB_G0293124
DPU_G0069236
PPL_06265
DFA_10173
DLA_03775
kxcB
7
PPL_07806
DFA_05578
DLA_00350
DDB_G0274889
DPU_G0066958
DFA_07541
DFA_05659
gxcX
8
DDB_G0281047
DPU_G0061832
PPL_03888
DFA_11267
DLA_06147
gxcEE
9
DDB_G0293266
DPU_G0063332
PPL_11372
DFA_10908
DLA_00045
gxcY
10
DDB_G0285859
DPU_G0065600
DLA_07142
PPL_02359
DFA_12924
gxcP
11
DDB_G0289859
DPU_G0065986
DLA_07265
PPL_00748
DFA_09369
kxcA
12
DDB_G0278417
DPU_G0057146
PPL_03688
DFA_10606
DLA_09323
xacB
13
DDB_G0294533
DPU_G0063182
PPL_10521
DFA_03851
DLA_09485
roco5
14
DDB_G0280087
DPU_G0062438
PPL_04359
DFA_04051
DLA_07453
gxcS
15
DDB_G0269610
DPU_G0069476
DLA_10729
PPL_08706
DFA_05049
gxcT
16
DDB_G0282271
DPU_G0053238
DLA_09914
PPL_07529
DFA_01552
gxcV
17
DDB_G0291007
DPU_G0061232
PPL_02014
DFA_02919
DLA_02462
gxcK
18
PPL_05961
DFA_01530
DLA_07721
DDB_G0293396
DPU_G0052190
gxcO
veg lawn agg. tip slug culm. fruit. psp pst veg spore stalk cup
DDBa DDBb DPUa DPUb DLA PPLa PPLb DFAa DFAb PPLc
c.0 c.24 c.48
c.0 c.8 c.16 c.24
DDB28a DDB28b DPU30a DPU30b
DDB54a DDB54b DPU56a DPU56b
DFA41a DFA41b DFA60a DFA60b
RhoGEF
CH
PH
IQ
VHP
RasGEF
RasGEFN
RCC1
S_TKc
Pkinase_Tyr
FYVE
Ion_trans
Arm
DUSP
Ubiquitin_3
UCH
LRR
smallGTPase
COR
RhoGAP
ZnF_C2H2

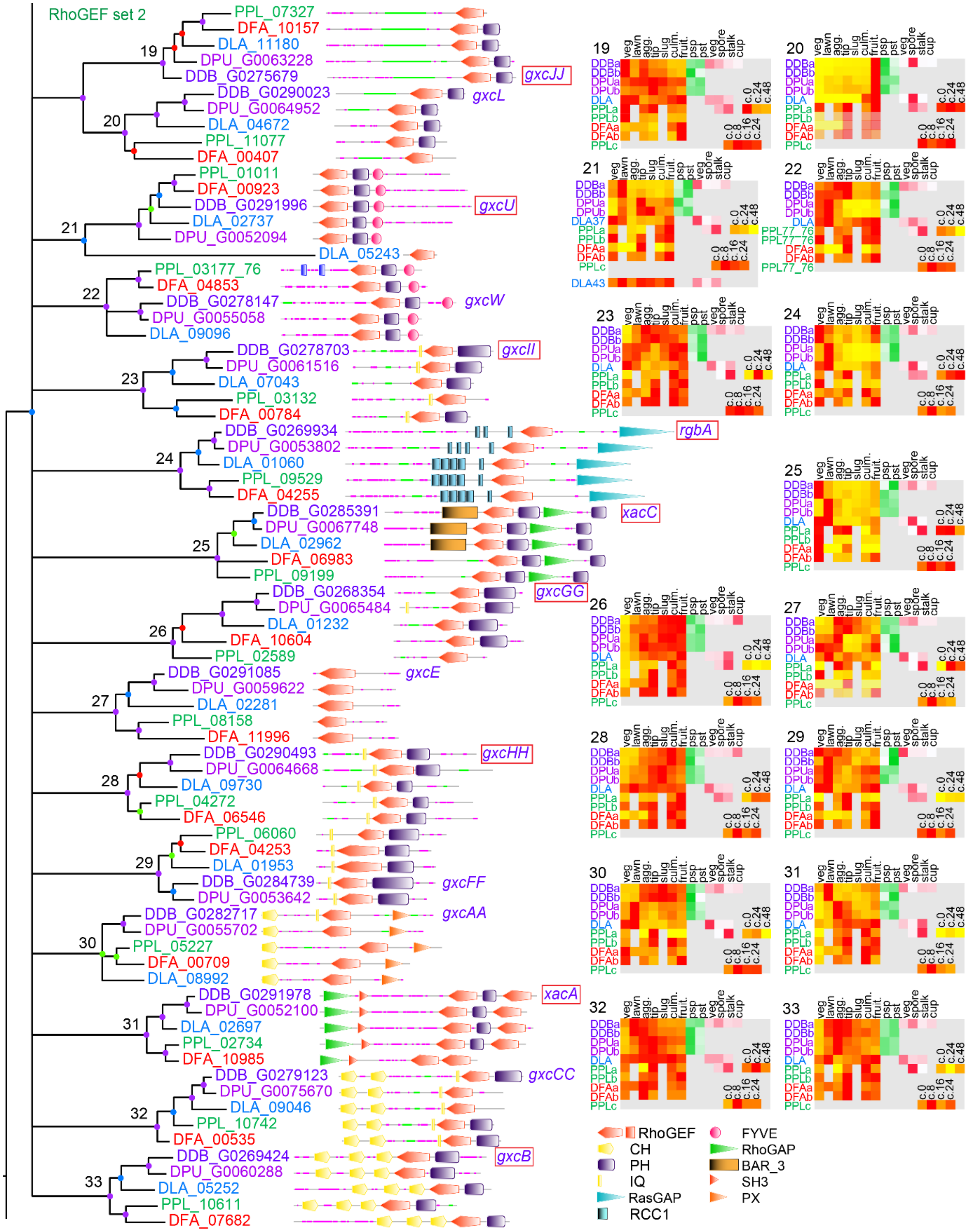
RhoGEF set 2
PPL_07327
DFA_10157
DLA_11180
DPU_G0063228
DDB_G0275679
gxcJJ
DDB_G0290023
gxcL
DPU_G0064952
DLA_04672
PPL_11077
DFA_00407
PPL_01011
DFA_00923
DDB_G0291996
gxcU
DLA_02737
DPU_G0052094
DLA_05243
PPL_03177_76
DFA_04853
DDB_G0278147
gxcW
DPU_G0055058
DLA_09096
DDB_G0278703
gxcII
DPU_G0061516
DLA_07043
PPL_03132
DFA_00784
DDB_G0269934
rgbA
DPU_G0053802
DLA_01060
PPL_09529
DFA_04255
DDB_G0285391
xacC
DPU_G0067748
DLA_02962
DFA_06983
PPL_09199
DDB_G0268354
gxcGG
DPU_G0065484
DLA_01232
DFA_10604
PPL_02589
DDB_G0291085
gxcE
DPU_G0059622
DLA_02281
PPL_08158
DFA_11996
DDB_G0290493
gxcHH
DPU_G0064668
DLA_09730
PPL_04272
DFA_06546
PPL_06060
DFA_04253
DLA_01953
DDB_G0284739
gxcFF
DPU_G0053642
DDB_G0282717
gxcAA
DPU_G0055702
PPL_05227
DFA_00709
DLA_08992
DDB_G0291978
xacA
DPU_G0052100
DLA_02697
PPL_02734
DFA_10985
DDB_G0279123
gxcCC
DPU_G0075670
DLA_09046
PPL_10742
DFA_00535
DDB_G0269424
gxcB
DPU_G0060288
DLA_05252
PPL_10611
DFA_07682
19 20 21 22 23 24 25 26 27 28 29 30 31 32 33
veg lawn agg. tip slug culm. fruit. psp pst veg spore stalk cup
DDBa DDBb DPUa DPUb DLA PPLa PPLb DFAa DFAb PPLc
DLA37 DLA43 PPL77_76
c.0 c.24 c.48
c.0 c.8 c.16 c.24
RhoGEF
CH
PH
IQ
RasGAP
RCC1
FYVE
RhoGAP
BAR_3
SH3
PX

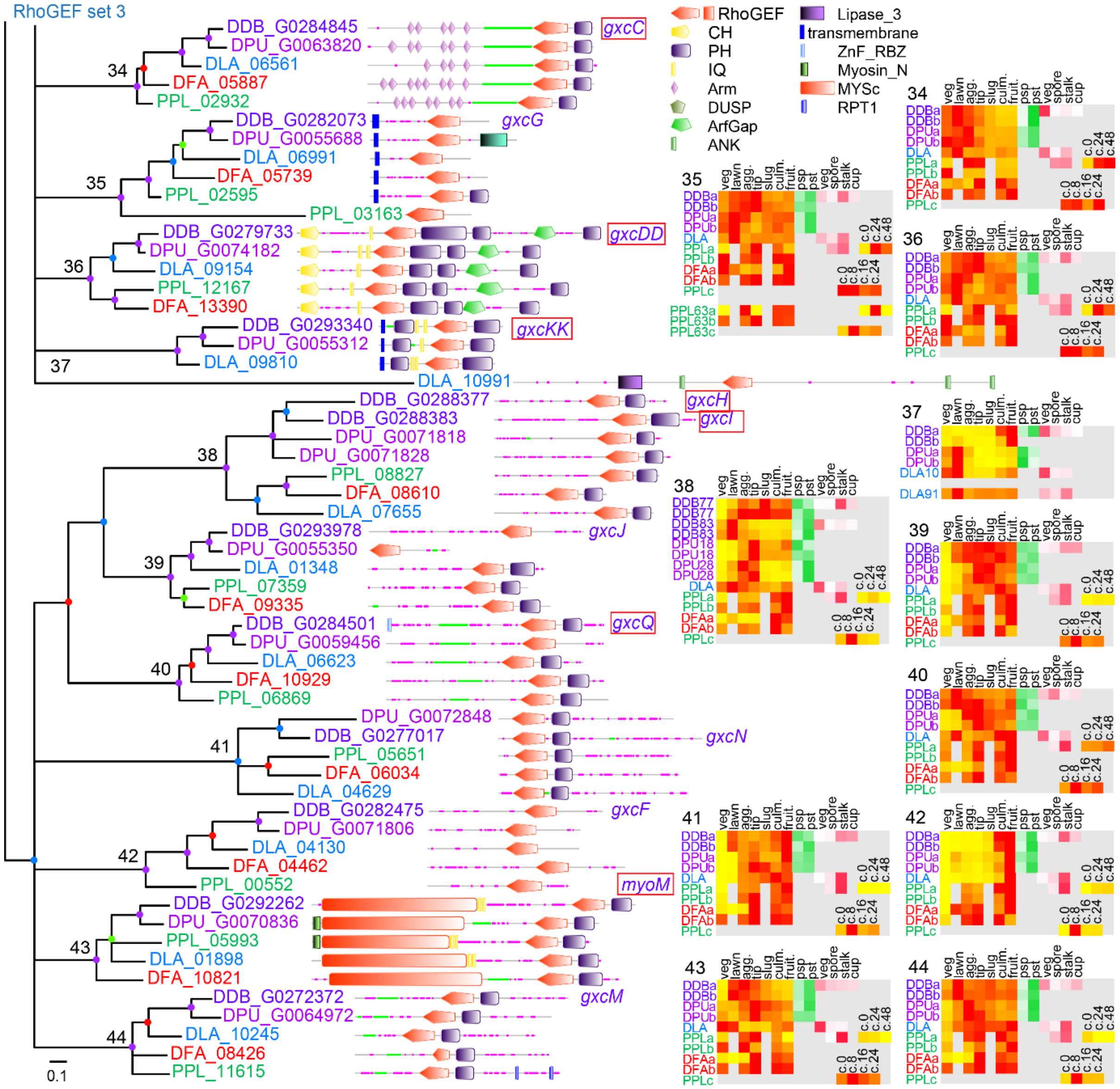

Figure S9. Rho Guanine Nucleotide Exchange Factors – DH family

Putative RhoGEFs were identified by query of Interpro scans of the five Dictyostelid proteomes with the domain identifier IPR000219 and BLAST searches as detailed in Methods. A phylogenetic tree was inferred from the isolated RhoGEF domain sequences from all retrieved proteins and annotated with protein functional domains and gene expression profiles as outlined in the legend to Fig. S1.

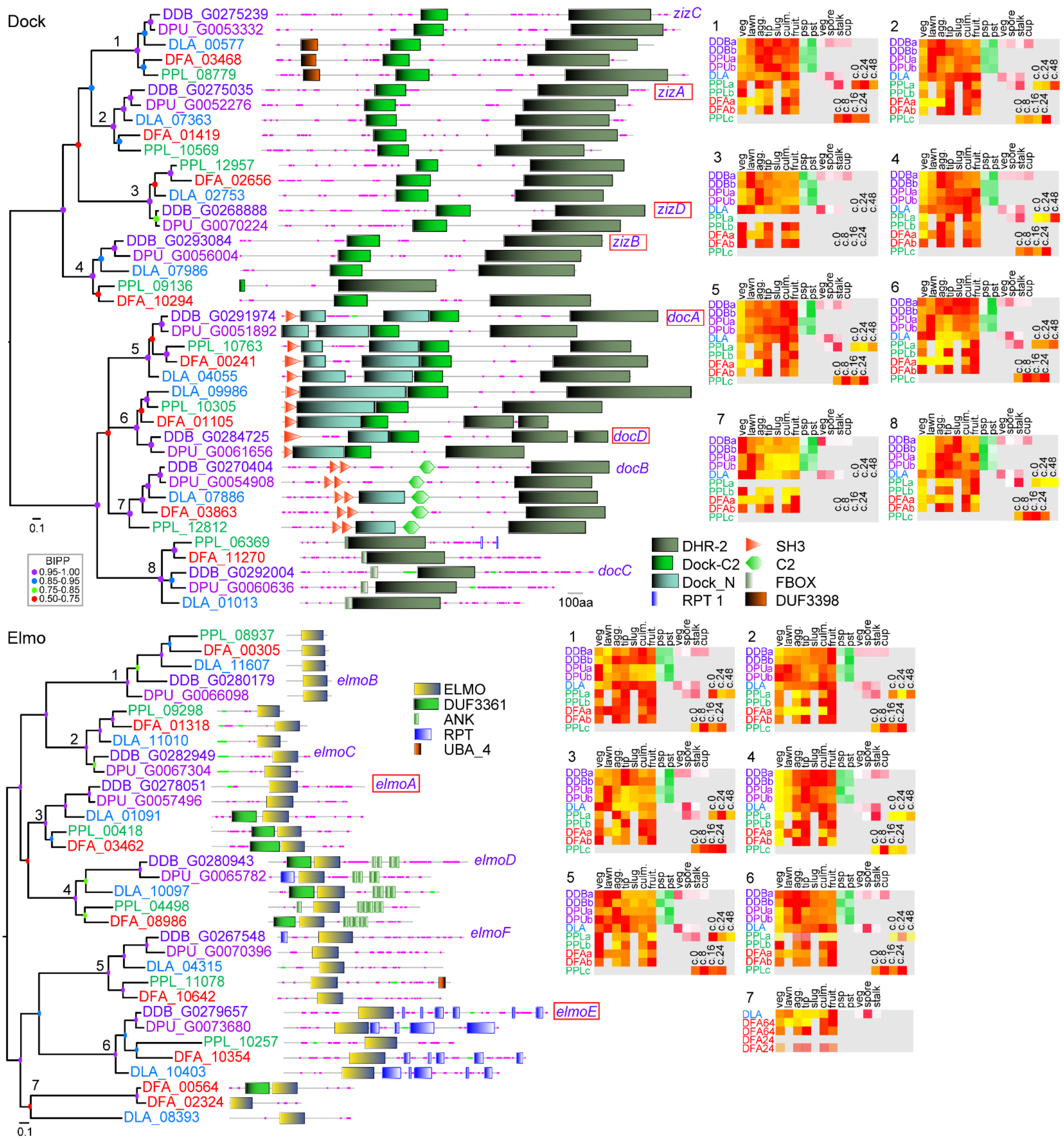

Figure S10. Rho Guanine Nucleotide Exchange Factors – Dock-Elmo family
Putative Dock and Elmo proteins were identified by query of Interpro scans of Dictyostelid proteomes with domain identifiers IPR027357 (DHR-2) / IPR037808 (Dock-2) for Dock and IPR006816 for ELMO and by BLAST searches as detailed in Methods. Phylogenetic trees were inferred and annotated with protein functional domains and gene expression profiles as outlined in the legend to Fig. S1.

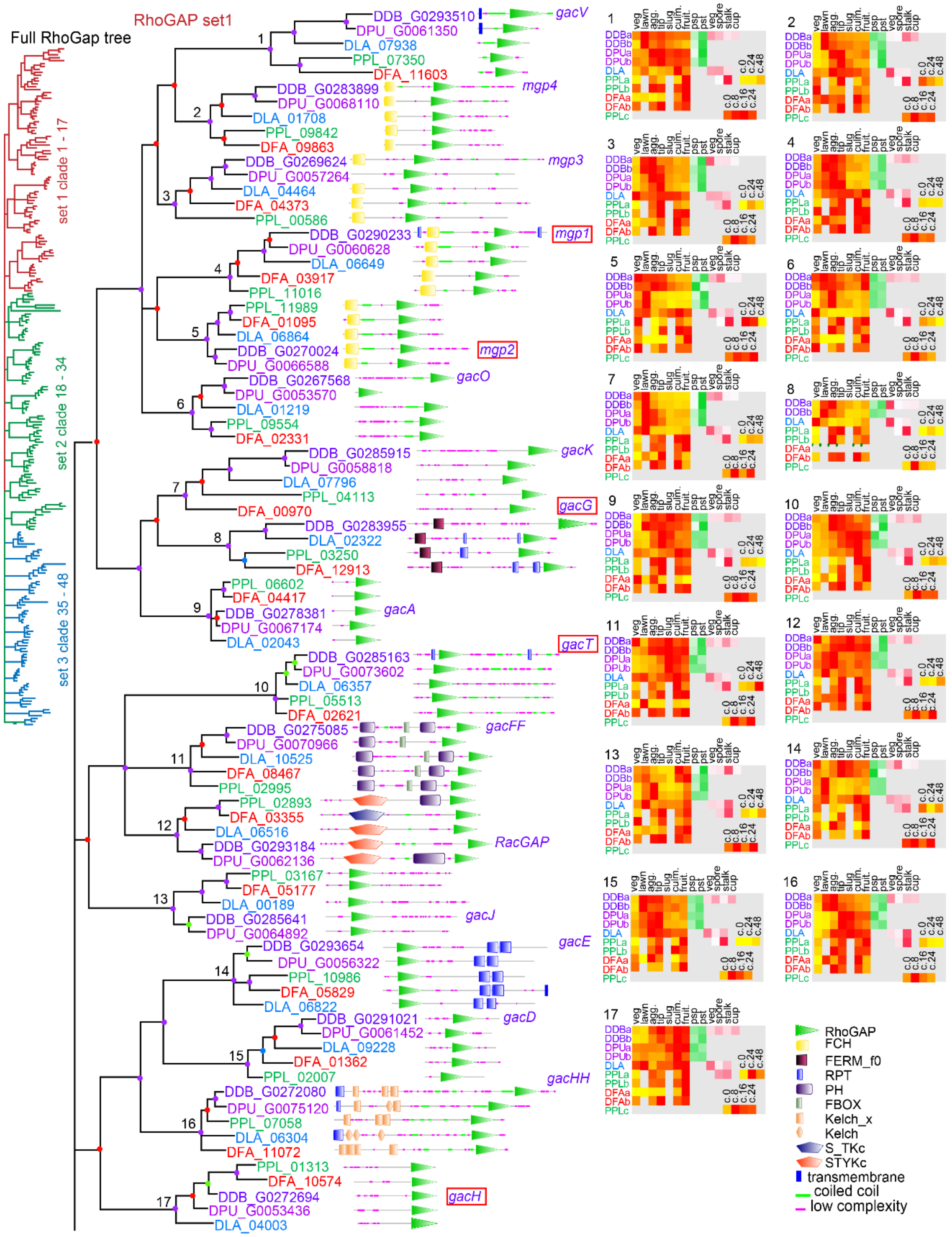
Full RhoGap tree
RhoGAP set1
set 1 clade 1 - 17
set 2 clade 18 - 34
set 3 clade 35 - 48
1
DDB_G0293510
DPU_G0061350
DLA_07938
PPL_07350
DFA_11603
gacV
2
DDB_G0283899
DPU_G0068110
DLA_01708
PPL_09842
DFA_09863
mgp4
3
DDB_G0269624
DPU_G0057264
DLA_04464
DFA_04373
PPL_00586
mgp3
4
DDB_G0290233
DPU_G0060628
DLA_06649
DFA_03917
PPL_11016
mgp1
5
PPL_11989
DFA_01095
DLA_06864
DDB_G0270024
DPU_G0066588
mgp2
6
DDB_G0267568
DPU_G0053570
DLA_01219
PPL_09554
DFA_02331
gacO
7
DDB_G0285915
DPU_G0058818
DLA_07796
PPL_04113
DFA_00970
gacK
8
DDB_G0283955
DLA_02322
PPL_03250
DFA_12913
gacG
9
PPL_06602
DFA_04417
DDB_G0278381
DPU_G0067174
DLA_02043
gacA
10
DDB_G0285163
DPU_G0073602
DLA_06357
PPL_05513
DFA_02621
gacT
11
DDB_G0275085
DPU_G0070966
DLA_10525
DFA_08467
PPL_02995
gacFF
12
PPL_02893
DFA_03355
DLA_06516
DDB_G0293184
DPU_G0062136
RacGAP
13
PPL_03167
DFA_05177
DLA_00189
DDB_G0285641
DPU_G0064892
gacJ
14
DDB_G0293654
DPU_G0056322
PPL_10986
DFA_05829
DLA_06822
gacE
15
DDB_G0291021
DPU_G0061452
DLA_09228
DFA_01362
PPL_02007
gacD
16
DDB_G0272080
DPU_G0075120
PPL_07058
DLA_06304
DFA_11072
gacHH
17
PPL_01313
DFA_10574
DDB_G0272694
DPU_G0053436
DLA_04003
gacH
veg lawn agg. tip slug culm. fruit. psp pst veg spore stalk cup
DDBa DDBb DPUa DPUb DLA PPLa PPLb DFAa DFAb PPLc
c.0 c.24 c.48
c.0 c.8 c.16 c.24
RhoGAP
FCH
FERM_f0
RPT
PH
FBOX
Kelch_x
Kelch
S_TKc
STYKc
transmembrane
coiled coil
low complexity

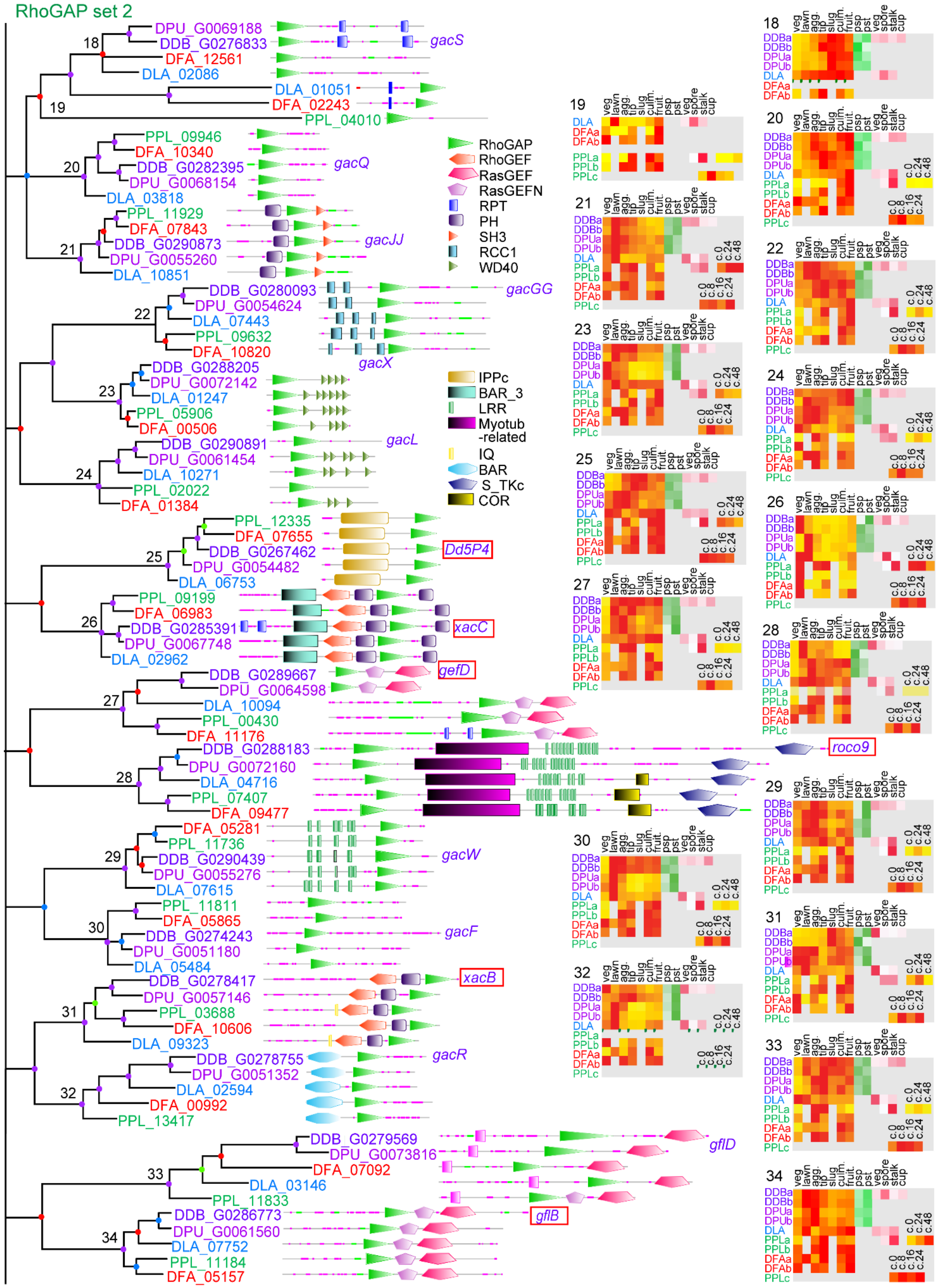
RhoGAP set 2
18
DPU_G0069188
DDB_G0276833
gacS
DFA_12561
DLA_02086
DLA_01051
DFA_02243
19
PPL_04010
20
PPL_09946
DFA_10340
DDB_G0282395
gacQ
DPU_G0068154
DLA_03818
21
PPL_11929
DFA_07843
DDB_G0290873
gacJJ
DPU_G0055260
DLA_10851
22
DDB_G0280093
gacGG
DPU_G0054624
DLA_07443
PPL_09632
DFA_10820
gacX
23
DDB_G0288205
DPU_G0072142
DLA_01247
PPL_05906
DFA_00506
24
DDB_G0290891
gacL
DPU_G0061454
DLA_10271
PPL_02022
DFA_01384
25
PPL_12335
DFA_07655
DDB_G0267462
Dd5P4
DPU_G0054482
DLA_06753
26
PPL_09199
DFA_06983
DDB_G0285391
xacC
DPU_G0067748
DLA_02962
gefD
27
DDB_G0289667
DPU_G0064598
DLA_10094
PPL_00430
DFA_11176
28
DDB_G0288183
roco9
DPU_G0072160
DLA_04716
PPL_07407
DFA_09477
29
DFA_05281
PPL_11736
DDB_G0290439
gacW
DPU_G0055276
DLA_07615
30
PPL_11811
DFA_05865
DDB_G0274243
gacF
DPU_G0051180
DLA_05484
31
DDB_G0278417
xacB
DPU_G0057146
PPL_03688
DFA_10606
DLA_09323
32
DDB_G0278755
gacR
DPU_G0051352
DLA_02594
DFA_00992
PPL_13417
33
DDB_G0279569
DPU_G0073816
gflD
DFA_07092
DLA_03146
PPL_11833
34
DDB_G0286773
gflB
DPU_G0061560
DLA_07752
PPL_11184
DFA_05157
RhoGAP
RhoGEF
RasGEF
RasGEFN
RPT
PH
SH3
RCC1
WD40
IPPc
BAR_3
LRR
Myotub
-related
IQ
BAR
S_TKc
COR
veg
lawn
agg.
tip
slug
culm.
fruit.
psp
pst
veg
spore
stalk
cup
DDBa
DDBb
DPUa
DPUb
DLA
PPLa
PPLb
PPLc
DFAa
DFAb
c.0
c.8
c.16
c.24
c.0
c.24
c.48

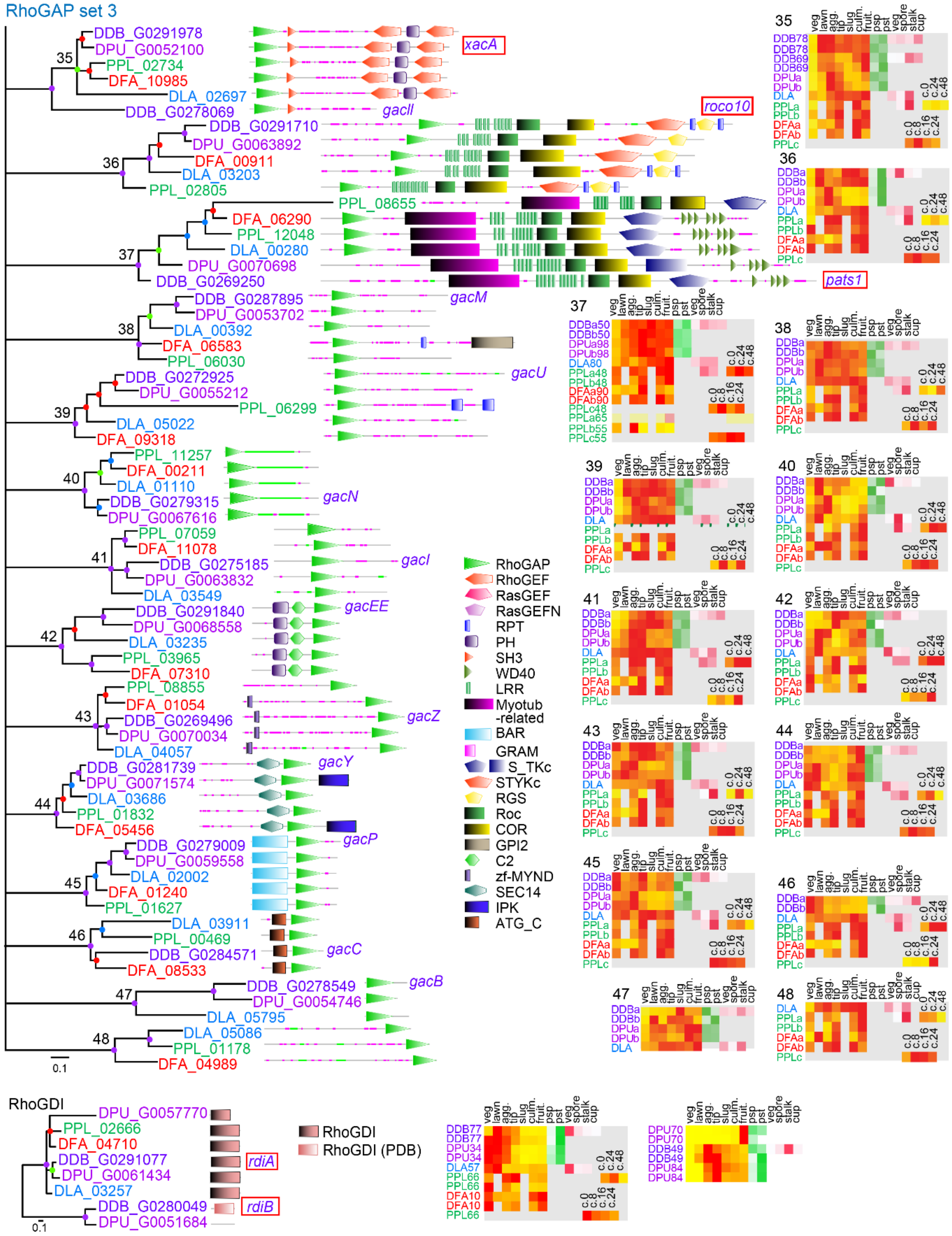

Figure S11. Rho GTPase Activating Proteins and Rho GDP-Dissociation Inhibitor
Sequences with RhoGAP and RhoGDI domains were retrieved from five Dictyostelid genomes with Interpro identifiers IPR000198 and IPR000406, respectively and by BLAST searches as detailed in Methods. Phylogenetic trees were inferred from the isolated RhoGAP domain sequences and full GDI proteins, and annotated with protein functional domains and gene expression profiles as outlined in Methods and the legend to Fig. S1.

RAS AND RAP GTPASES

The Ras (Rat sarcoma virus) GTPases were initially identified as viral oncogenes that were highly identical to human proteins. The human proteins were found to mediate growth factor activation of the cell cycle and are rendered oncogenic by mutations that keep Ras in a GTP-bound state. In addition to cell proliferation, Ras GTPases are also involved in regulating cell migration, differentiation, apoptosis and cell adhesion. Activation of phosphatidyl-inositol 3 kinase (PI3K) and of the Mitogen Activated Protein (MAP) kinase cascade are major functions of Ras GTPases in humans (Simanshu et al. 2017).

Ddis has 36 related Ras, Rap (Ras-proximate) and Rsm (Ras superfamily member) GTPases, of which 18 are conserved throughout Dictyostelia (Fig. S12). RasB, activated by GefQ regulates both cytokinesis, cell polarity and phagocytosis, by acting on myosin II (Mondal et al. 2008). RasS is activated by GefB and deletion of either *rasS* or *gefB* causes mild defects in motility and macropinocytosis (Wilkins et al. 2000a). RasG has critical roles in chemotaxis, cytokinesis and macropinocytosis, and can be activated by either GflB, GefR and GefF (Kae et al. 2007; Williams et al. 2019a), and inactivated by the GAPs NfaA, NgaP (C2GAP1) or IqgC (Zhang et al. 2008; Xu et al. 2017; Marinović et al. 2019). RasD is likely activated by GefE (Chattwood et al. 2013) and *rasD⁻* cells are defective in slug phototaxis and thermotaxis (Wilkins et al. 2000b), and in prestalk differentiation (Gruenheit et al. 2018). RasC, activated by GefA, is an essential intermediate of cAMP-induced chemotaxis and adenylate cyclase A activation (Lim et al. 2001; Bolourani et al. 2006).

RapA has major roles in cell adhesion and in myosin assembly during chemotaxis and cytokinesis (Jeon et al. 2007; Plak et al. 2014). RapA is activated by both GefU (GbpD) and GflB (Kortholt et al. 2006; Liu et al. 2016) and inactivated by Rapgap1 and Rapgap3 (Jeon et al. 2007; Jeon et al. 2009). RapC null mutants show defective cytokinesis, reduced motility and formation of multiple tips on aggregates (Park et al. 2018). *RasY* and *rasZ*, two members of a set of five amplifed *Ddis ras* genes, are upregulated after infection with *Mycobacterium marinum* and *Legionella pneumophila* bacteria (Kjellin et al. 2019), suggesting that the many species-specific amplifications of small GTPases may reflect niche-specific adaptations. Disruption of the highly gamete-enriched *rasZ* did not cause alterations in growth rate, asexual development, sexual cell fusion and zygote development (Muramoto et al. 2005).

Phr was detected by proteomics as a member of the Sca1 complex that regulates F-actin dynamics at the leading edge of chemotaxing cells. However, whereas deletion of other members of the Sca1 complex severely impaired aggregation, loss of Phr and its interactor GefH only showed a small delay of development (Charest et al. 2010). Loss of Rheb increases phagocytosis, while loss of the Rheb inactivating GAP, Tsc2, suppresses phagocytosis. These effects require the TOR complex2 components Rictor, Sin1 and Lst8 (Rosel et al. 2012).

Ras guanine nucleotide exchange factors.

The *Ddis* genome contains 29 RasGEFs that cluster in 28 clades (*gefI* and *gefJ* in cluster 1 are the result of a gene duplication occurring in *Ddis* only), of which 26 clades are conserved throughout Dictyostelia (Fig. S13). Seventeen Ras GTPases have been functionally investigated. GefA, GefH, GefB, GefQ and GefE are discussed above with their target GTPases. GefS shows prolonged phosphorylation in *pten⁻* cells, but *gefS⁻* cells have no obvious phenotype (Tang et al. 2011). *GefR*, *gefG*, *gefC* and *gefD* null cells show no obvious developmental phenotype, but display aberrant Ras activation at the leading edge (Secko et al. 2004; Wilkins et al. 2005; Kortholt et al. 2013). Mutants with an insertion in *gefV or gefL* display aberrant cAMP waves and developmental defects (Sawai et al. 2008). *GflB⁻* cells show defects in phagocytosis, cytokinesis and chemotaxis (Senoo et al. 2016; Inaba et al. 2017). GbpC (GefT) is a multidomain Roco protein that is discussed in the section “Small families of GTPases”. *gbpD⁻* mutants show improved chemotaxis due to suppression of lateral pseudopod formation (Bosgraaf et al. 2005). g*efM⁻* mutants form small flat aggregates that do not develop further. cAMP induced chemotaxis and adenylate cyclase activation are impaired in *gefM⁻,* but guanylate cyclase is hyperactivated (Arigoni et al. 2005). GefF could not be knocked out, suggesting involvement in growth (Wilkins et al. 2005).

Ras GTPase activating proteins

The *Ddis* genome contains 15 rasGAP genes which are conserved throughout Dictyostelia and 10 of those have been functionally analysed (Fig. S14). RGBARG (*rgbA*) regulates simultaneously Rho and Ras during macropinosome and phagosome formation (see “Rho GTPases” section); in particular it can inactivate RasB, RasD, RasG, RasS and RapA (Buckley et al. 2020). *AxeB* (neurofibromin1) harbours a massive deletion in *Ddis* axenic mutants, which allows cells to form larger pinosomes and phagosomes. This suggests that the normal function of this RasGAP is to constrain Ras activity to a smaller area of the cell cortex (Bloomfield et al. 2015). NgaP, IqgC and NfaA were discussed above with their target GTPases. C2gapB (RasGAP2) and RapGAP3

inhibit Ras activity at the rear of the cell (Li et al. 2018). RgaA (RasGAP1) null cells are defective in cytokinesis and arrest development at the slug stage, with differentiation of only a few spores and no stalk cells (Lee et al. 1997). GapA (IqgB) acts together with myosin II and cortexillin in mechanical stress sensing, cytokinesis, and regulation of Ras activity during chemotaxis (Kee et al. 2012; Lee et al. 2010). DG1112 was identified as the disrupted gene in an insertional mutant with very thin stalks (http://dictybase.org/gene/DDB_G0276463) and in a separate mutagenesis screen DDB_G0284177 showed aberrant culmination (Sawai et al. 2008).

Rap GTPase activating proteins

RapGAPs have a conserved RapGAP domain of ~200 amino acids and 10 well conserved genes are present in Dictyostelia (Fig. S15). Rapgap1 and Rapgap3, which inactivate RapA, and Tcs2, which inactivates Rheb were discussed above. *RapgapB*$^{-}$ cells show higher levels of GTP-bound RapA, decreased cell-cell adhesion and aberrant prestalk gene expression (Parkinson et al. 2009). *Rapgap9*$^{-}$ cells are multinucleate and show defective chemotaxis (Mun et al. 2014).

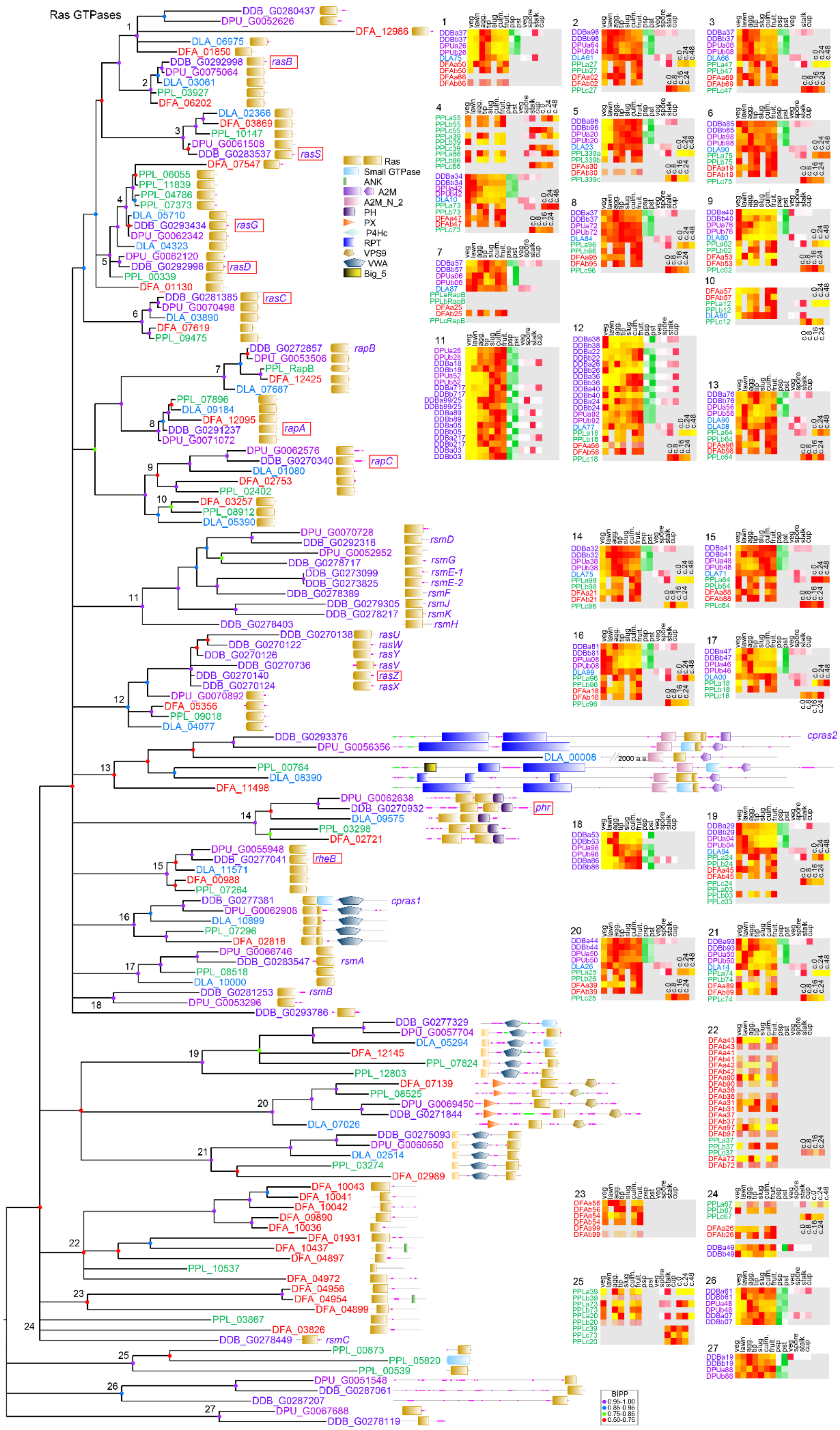
Ras GTPases
DDB_G0280437
DPU_G0052626
DFA_12986
DLA_06975
DFA_01850
DDB_G0292998
rasB
DPU_G0075064
DLA_03061
PPL_03927
DFA_06202
DLA_02366
DFA_03869
PPL_10147
DPU_G0061508
DDB_G0283537
rasS
DFA_07547
PPL_06055
PPL_11839
PPL_04786
PPL_07373
DLA_05710
DDB_G0293434
rasG
DPU_G0062342
DLA_04323
DPU_G0062120
DDB_G0292996
rasD
PPL_00339
DFA_01130
DDB_G0281385
rasC
DPU_G0070498
DLA_03890
DFA_07619
PPL_09475
DDB_G0272857
rapB
DPU_G0053506
PPL_RapB
DFA_12425
DLA_07687
PPL_07896
DLA_09184
DFA_12095
DDB_G0291237
rapA
DPU_G0071072
DPU_G0062576
DDB_G0270340
rapC
DLA_01080
DFA_02753
PPL_02402
DFA_03257
PPL_08912
DLA_05390
DPU_G0070728
rsmD
DDB_G0292318
DPU_G0052952
rsmG
DDB_G0278717
DDB_G0273099
rsmE-1
DDB_G0273825
rsmE-2
DDB_G0278389
rsmF
DDB_G0279305
rsmJ
DDB_G0278217
rsmK
DDB_G0278403
rsmH
DDB_G0270138
rasU
DDB_G0270122
rasW
DDB_G0270126
rasY
DDB_G0270736
rasV
DDB_G0270140
rasZ
DDB_G0270124
rasX
DPU_G0070892
DFA_05356
PPL_09018
DLA_04077
DDB_G0293376
cpras2
DPU_G0056356
DLA_00008
2000 a.a.
PPL_00764
DLA_08390
DFA_11498
DPU_G0062638
DDB_G0270932
phr
DLA_09575
PPL_03298
DFA_02721
DPU_G0055948
DDB_G0277041
rheB
DLA_11571
DFA_00988
PPL_07264
DDB_G0277381
cpras1
DPU_G0062908
DLA_10899
PPL_07296
DFA_02818
DPU_G0066746
DDB_G0283547
rsmA
PPL_08518
DLA_10000
DDB_G0281253
rsmB
DPU_G0053296
DDB_G0293786
DDB_G0277329
DPU_G0057704
DLA_05294
DFA_12145
PPL_07824
PPL_12803
DFA_07139
PPL_08525
DPU_G0069450
DDB_G0271844
DLA_07026
DDB_G0275093
DPU_G0060650
DLA_02514
PPL_03274
DFA_02989
DFA_10043
DFA_10041
DFA_10042
DFA_09890
DFA_10036
DFA_01931
DFA_10437
DFA_04897
PPL_10537
DFA_04972
DFA_04956
DFA_04954
DFA_04899
PPL_03867
DFA_03826
DDB_G0278449
rsmC
PPL_00873
PPL_05820
PPL_00539
DPU_G0051548
DDB_G0287061
DDB_G0287207
DPU_G0067688
DDB_G0278119
Ras
Small GTPase
ANK
A2M
A2M_N_2
PH
PX
P4Hc
RPT
VPS9
VWA
Big_5
BIPP
veg
lawn
agg.
tip
slug
culm.
fruit.
psp
pst
spore
stalk
cup

Figure S12. Conservation and change in the Ras-like GTPases across Dictyostelia.
The GTP-ases in Dictyostelid genomes were identified from Interpro scans as outlined in Methods and a pilot phylogenetic tree was prepared from all aligned sequences, in which the Ras and Rap GTPases formed a single clade. The sequences in this clade were further supplemented by BLAST queries using Ras and Rap sequences as bait. A final tree was constructed, which was annotated with protein functional domains and gene expression profiles as outlined in Methods and the legend to Fig. S1.

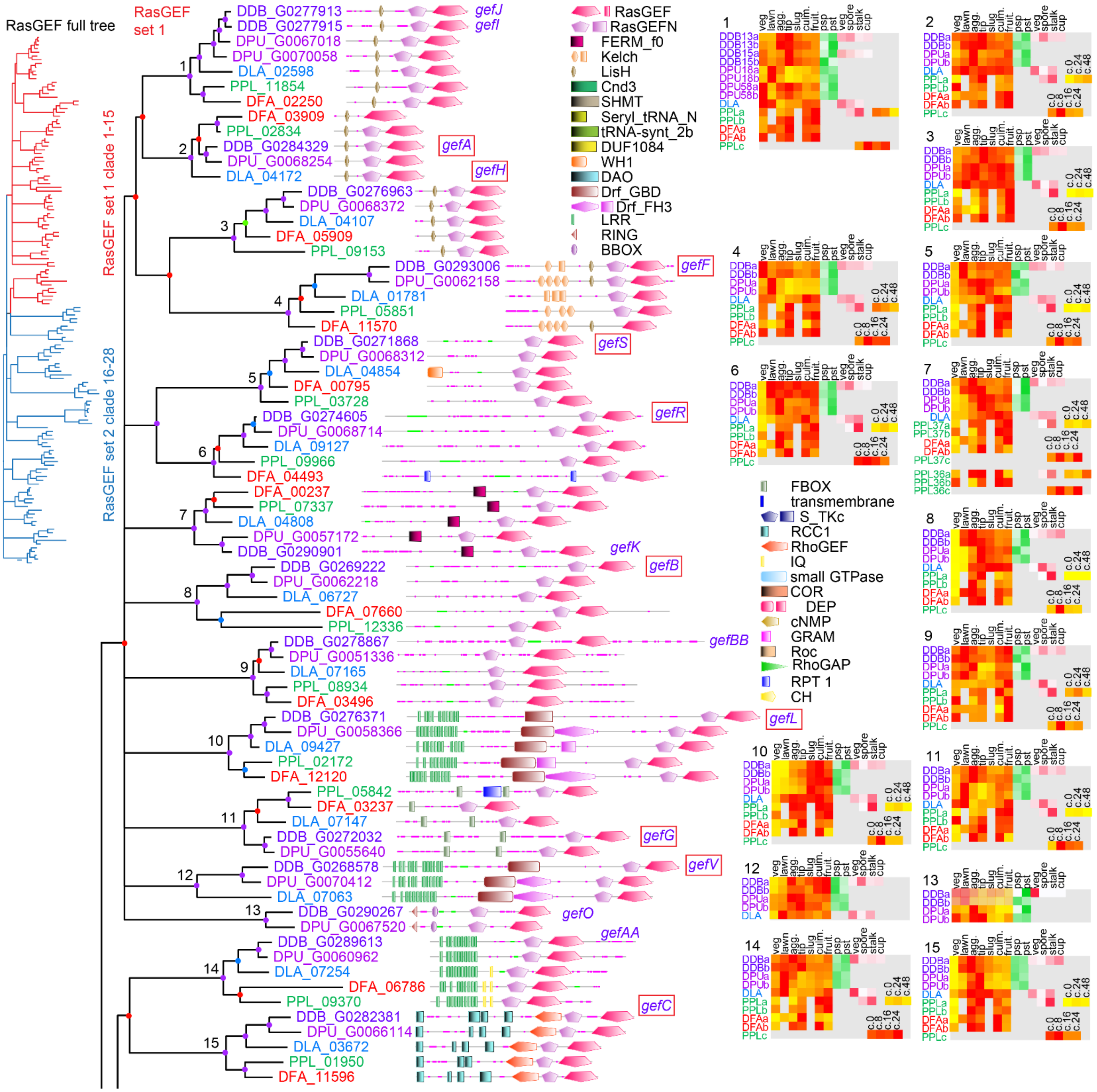

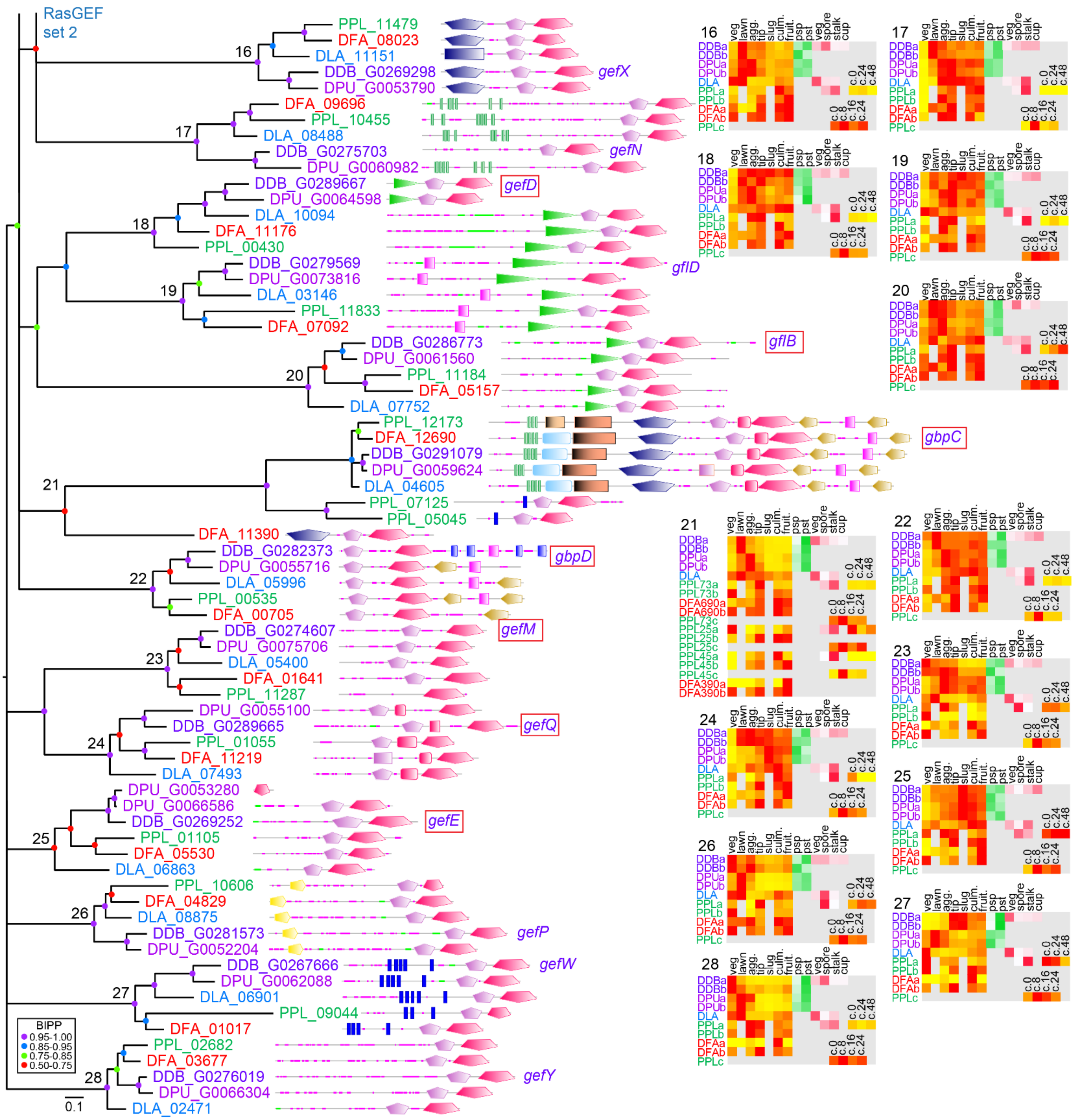

Figure S13. Ras Guanine Nucleotide Exchange Factors

Proteins with RasGEF domains were identified in Interpro scans of Dictyostelid genomes with Interpro identifier IPR023578 and by BLAST search as detailed in Methods. Phylogenetic trees were inferred from the isolated RasGEF sequences and annotated with protein functional domains and gene expression profiles as outlined in Methods and the legend to Fig. S1.

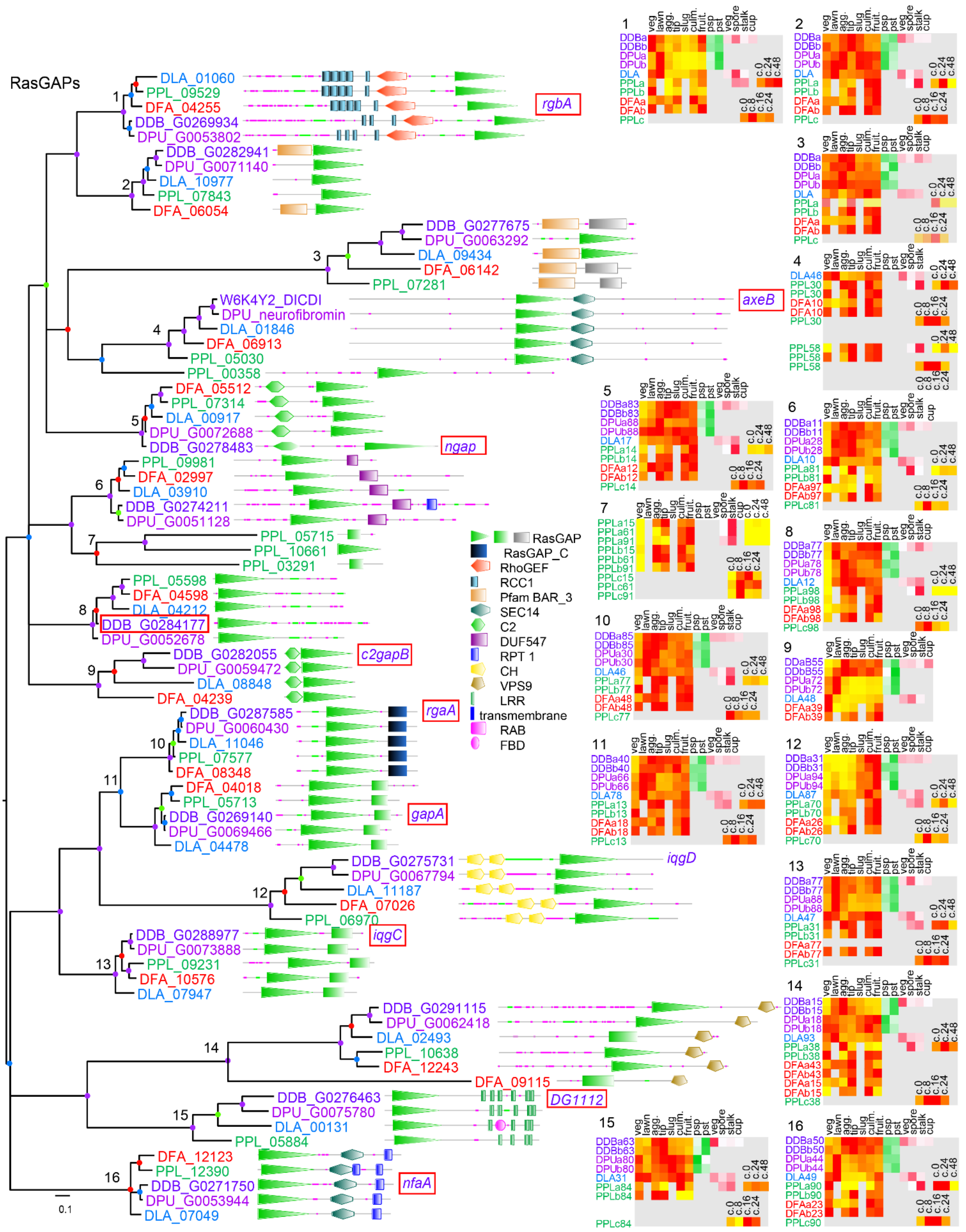

Figure S14. Ras GTPase activating proteins
Proteins with RasGAP domains were identified in Interpro scans of Dictyostelid proteomes with Interpro identifier IPR001936 and by BLAST search as detailed in Methods. Phylogenetic trees were inferred from the isolated RasGAP sequences, and annotated with protein functional domains and gene expression profiles as outlined in Methods and the legend to Fig. S1.

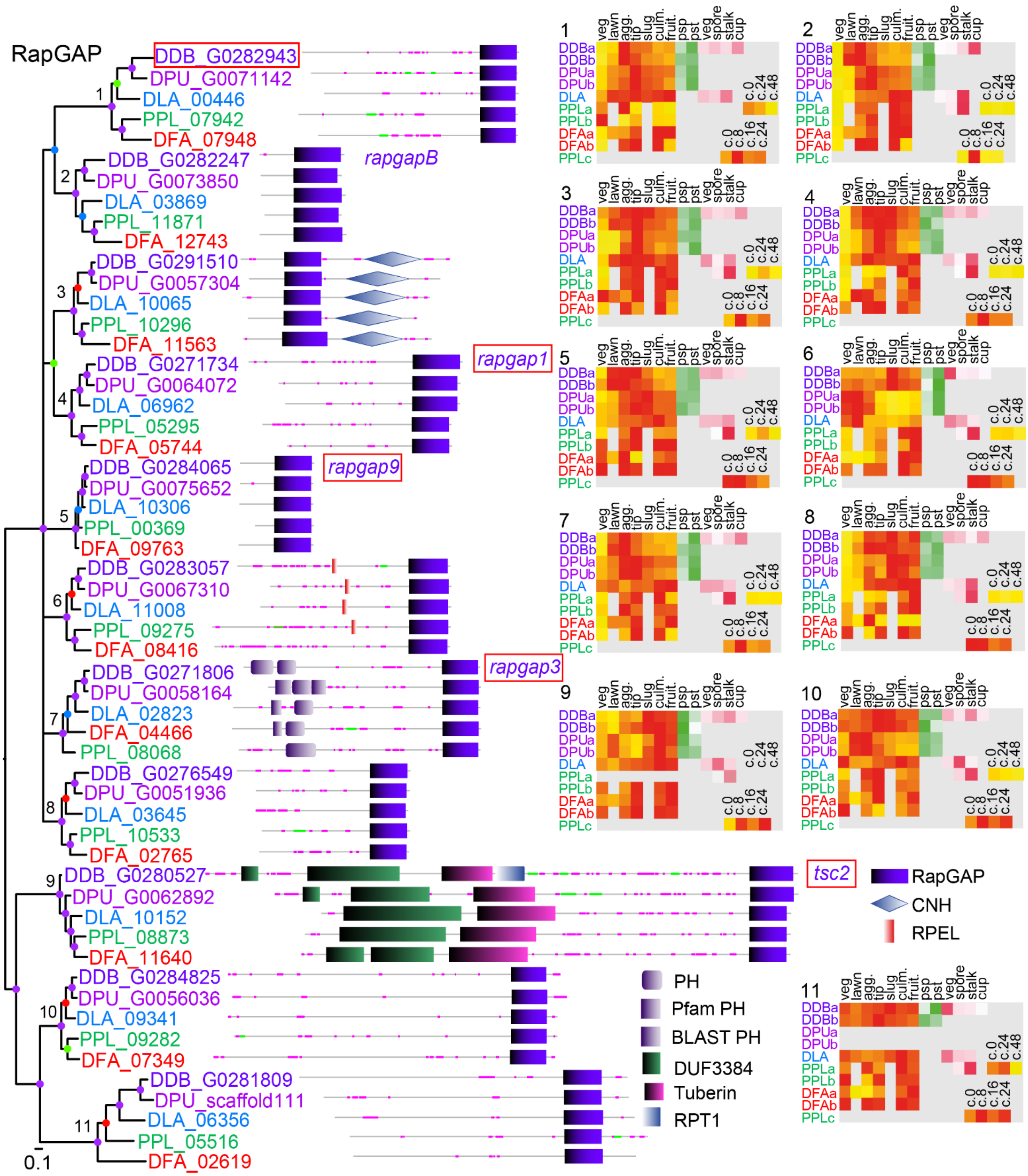

Figure S15. Rap GTPase Activating Proteins
Proteins with RapGAP domains were identified in Interpro scans of Dictyostelid proteomes with Interpro identifier IPR000331 and by BLAST search as detailed in Methods. Phylogenetic trees were inferred from the isolated RasGAP sequences, and annotated with protein functional domains and gene expression profiles as outlined in Methods and the legend to Fig. S1.

SMALL FAMILIES OF GTPASES

Some GTPases separated out as 5 distinct long branched clades in GTPase phylogenies. The largest group among those are the Roco GTPases. They are characterised by a tandem Roc (Ras of complex proteins) and COR (C-terminal of Roc) domain in addition to a range of other domains, amongst which protein kinase, RasGEF, RhoGEF and RhoGAP domains. The Roco GTPases were first identified in Dictyostelia, but are widely conserved in pro- and eukaryotes (Wauters et al. 2019). In human, mutations in the Roco GTPase LRRK2 are a cause of Parkinson's disease. The *Ddis* genome harbours 11 *roco* genes that subdivide into 9 clusters conserved throughout Dicytostelia (note that *qkgA* exists as two identical copies in the Ax4 strain due to partial duplication of chromosome 2). Cluster 1 has undergone gene expansion in *Ddis*. All *roco* genes have been functionally analysed (Fig. S16). QkgA mediates both chemoattractant-induced actin polymerisation (Kicka et al. 2011) and inhibition of cell proliferation by the autocrine proliferation repressor AprA (Abe et al. 2003; Phillips and Gomer 2012). Deletion of *roco11* increases fruiting body size, while deletion of *roco4* causes reduced cellulose synthesis by prestalk cells, resulting in feeble stalks (van Egmond and van Haastert 2010). Three Roco family proteins, Pats1, Roco9 and Roco10 carry an N-terminal RhoGAP domain. Pats1 is required for cytokinesis (Abysalh et al. 2003), while disruption of roco9 and roco10 did not reveal any apparent alteration in growth, cell division and development (van Egmond and van Haastert 2010). GbpC harbours additional cGMP binding domains, a RasGEF and a protein kinase domain and mediates cAMP-induced chemotaxis. Here, cAMP increases intracellular cGMP, which, after binding to GbpC, stimulates the intrinsic RasGEF domain to activate the intrinsic Roc GTPase domain, which in turn activates the intrinsic protein kinase (van Egmond et al. 2008). Roco5 harbours an N-terminal RhoGEF domain; a lesion in *roco5* results in aberrant cAMP signalling, slug migration and fruiting body formation (Sawai et al. 2008). Disruption of roco6, roco7 and roco8 did not result in altered cell growth, cell division and development (van Egmond and van Haastert 2010).

The 4 other small families have from one to three deeply conserved members in Dictyostelia. The Miro (Mitochondrial Rho) GTPase GemA has been functionally analyzed in *Ddis;* its loss causes a decrease in ATP content and mitochondrial mass and as a consequence decreased cell growth (Vlahou et al. 2011). The GPN (Gly-Pro-Asn) loop GTPases were studied in yeast and vertebrates. Gpn1 mediates RNA polymerase II transport to the nucleus, while Gpn2 and Gpn3 play roles in biogenesis of RNA polymerases II and III (Staresincic et al. 2011; Minaker et al. 2013). The Rag (Ras-related GTPases) mediate intracellular amino acid sensing by mTORC1 activation in other organisms (Kim and Kim 2016). The GTPase domain of the single Roco-like (Rol) GTPase is most similar to that of the Roco proteins, but it lacks their signature COR domain and complex domain architecture. Disruption of the gene encoding Rol did not result in an obvious phenotype (Torija et al. 2006).

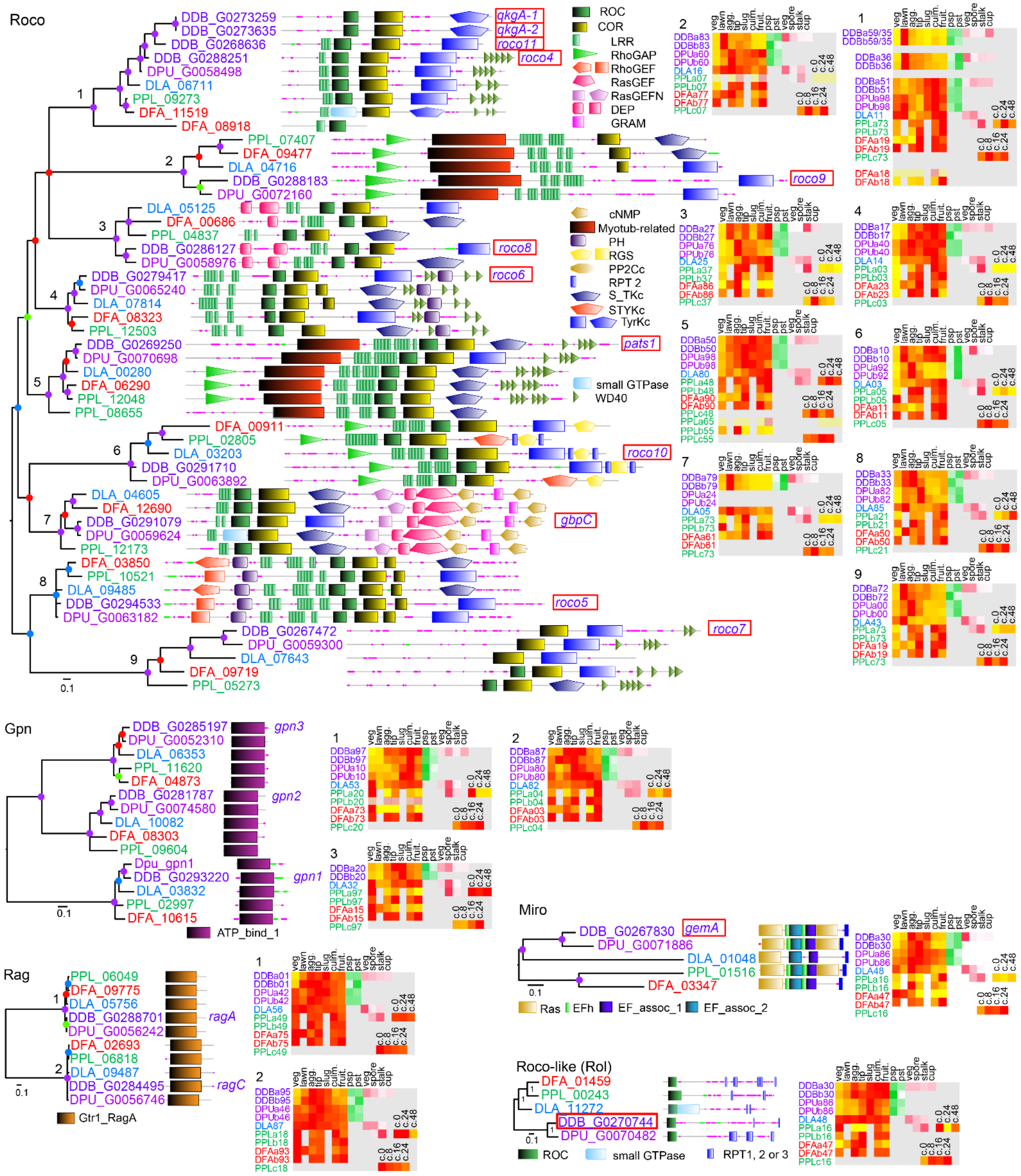

Figure S16. Roco, Gpn, Rag, Miro and Rol GTPases across Dictyostelia.

All Dictyostelid GTP-ases were identified from Interpro scans and collected into a single pilot phylogenetic tree as described in methods. The Roco, Gpn, Rag, Miro and Rol GTPases occupied distinct clades that were separated by long branches from the four major Arf-Sar, Rab-Ran, Rho-Rac and Ras-Rap clades. The GTPase domains of these smaller groupings were separately aligned and phylogenetic trees were inferred, which were annotated with functional domains and expression profiles as outlined in the legend to Fig. S1.

CLUSTER ANALYSIS

Figure S17. Annotated hierarchical tree of group 4 transcription profiles.

Hierarchical tree obtained by cluster analysis of the expression profiles of group 4 GTPase and regulator genes, as shown in in figure 6, but annotated with all gene names or Dictybase locus tags without the DDB_G0 prefix, the experimentally identified protein interactions, the effects of gene manipulation on phenotype and the protein localization at the branch tips. The figure is derived from Supplemental_Table_S4.xlsx, sheet “Group4”, which contains additional annotation with association of proteins with specific organelles and transcriptional changes in response to different food bacteria. The sheets “Fullprofile”, “BranchII”, “DdisOnly” and “Group4Complete” in Table S4.xlsx also archive the annotated hierarchical trees obtained with other subsets of the expression profiles of the five investigated species, and for group 4 with complete instead of alternative linkage.
Due to its size Figure S17 had to be displayed as a separate PDF file: Supplemental_Figure_S17.pdf

Figure S18. Comparison of hierarchical trees

The “Group4” based hierarchical tree was compared with trees based on the “Ddisonly”, “BranchII” and “Fullprofile” sets of transcription data using the dendextend package in R (Galili 2015). Sets of two trees are juxtaposed and the positions of genes in the “Group4” tree are connected by lines to their positions in each of the other trees. The figure shows that despite different overall tree topologies, most genes also cluster together when other subsets of the transcription data are used for the analyses. Note that the trees in figure S18 are horizontally flipped compared to the tree in figure 6.
Due to its size Figure S18 is displayed as a separate PDF file: Supplemental_Figure_S18.pdf

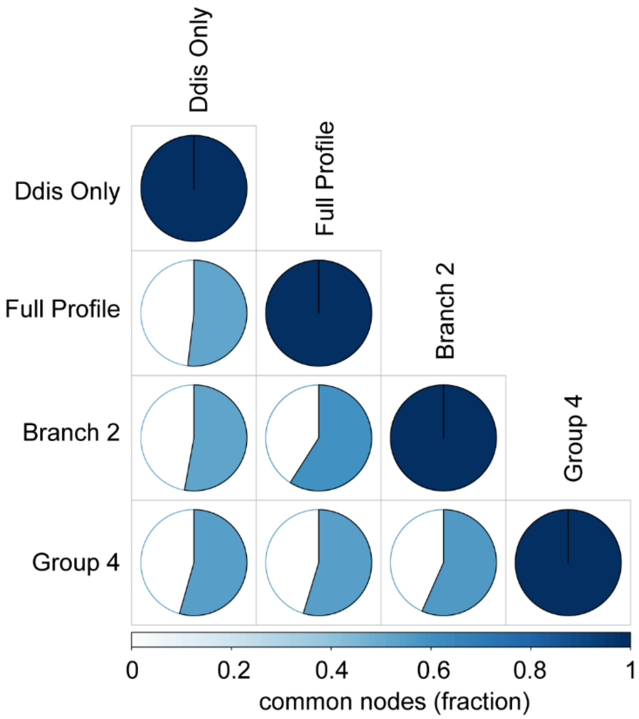

Figure S19. Common nodes in hierarchical trees derived from different transcriptome subsets

The fractions of common nodes in the tree comparisons of Figure S18 were calculated and plotted using the dendextend and corrplot packages in R (Galili 2015).

Figure S20. Statistical support for clustered genes

The four hierarchical trees obtained with the “Group4”, “Ddisonly”, “BranchII” and “Fullprofile” set of transcription data were bootstrapped with 1000 replicates using the pvclust package in R (Suzuki and Shimodaira 2006). Trees are displayed both as cladograms with the AU (Approximately Unbiased) p-value at the nodes and as dendrograms with the SI (Selective Inference) p-values at the nodes (Shimodaira and Terada 2019), both expressed as percentages. Nodes with values of 95% or higher are strongly statistically supported.
Due to its size Figure S20 is displayed as a separate PDF file: Supplemental_Figure_S20.pdf

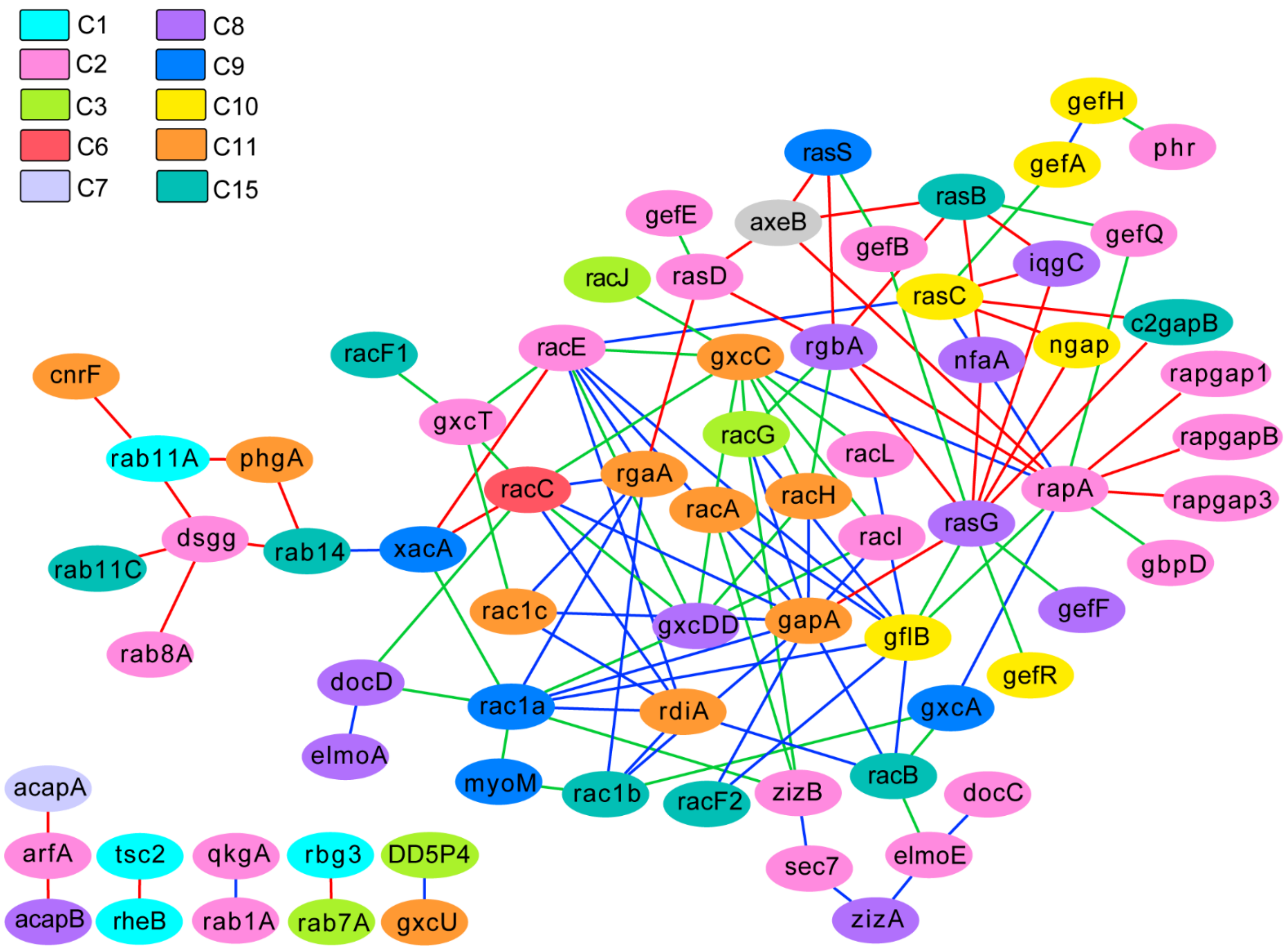

Figure S21. Network of established interactions between GTPases and their primary regulators
Experimentally confirmed interactions between GTPases, GEF, GAPs and other direct regulators as listed in Table S3.xlsx, sheet 4 were visualized as a network using Cytoscape (Shannon et al. 2003). The nodes are colour coded according to their positions in different clusters as identified by cluster analysis of Group 4 transcription data (see figure 6). Green and red lines indicate that a node has a GEF or GAP activity, respectively, on the GTPase it is connected to. Blue lines indicate interaction of a different nature.

SUPPLEMENTAL TABLES

Supplemental_Table_S1.xlsx. This spreadsheet collates that data on the presence of GTPases in taxon group representative Dictyostelia and the conservation of their functional domains and cell-type specific gene expression

Supplemental_Table_S2.xlsx. This spreadsheet collates that data on the presence of GEFs, GAPs and other direct GTPase regulators in taxon group representative Dictyostelia and the conservation of their functional domains and cell-type specific gene expression

Supplemental_Table_S3.xlsx. This spreadsheet contains literature data on experimentally established interactions between GTPases and their direct regulators, the cellular localization of the proteins and their functions as established by gene manipulation.

Supplemental_Table_S4.xlsx. This spreadsheet contains the input and results of the hierarchical cluster analysis of GTPases and their direct regulators based on similarity between gene expression profiles

Supplemental_Tables_S1-S4 are presented as separate supplemental files.

Supplemental_Table_S5. Recognized interactions per cluster over different hierarchical trees

Full profile			branch II			Group 4			Group 4 Comp.link.			*Ddis* Only		
Cluster	Interac. code	Interac. /cluster	Cluster	Interac. code	Interac. /cluster	Cluster	Interac. code	Interac. /cluster	Cluster	Interac. code	Interac. /cluster	Cluster	Interac. code	Interac. /cluster
1			1	$18	1	1	$13	1	1			1	$11	1
2	$13	1		$9	5	2	$16	1	2				$12	1
3				$10	4		$19	2	3	$14	1		$9	1
4				$7	2		$20	1		$17	2	2		
5				$11	2		$12	5		$5	1	3		
6				$14	2		$25	1	4			4	$10	3
7	$23	1		$19	1		$17	3	5	$12	1		$17	3
	$10	7		$20	1		$10	6		$10	3		$14	5
	$14	3		$23	1		$7	1		$17	1		$15	4
	$17	4		$24	2		$14	2		$13	1		$16	3
	$2	1		$17	1		$9	2	6	$10	2		$18	2
	$16	1		$25	1		$31	2		$14	2		$19	4
	$19	1	2	$12	1		$18	1		$19	1		$23	1
	$12	5	3	$11	1		$2	1		$20	1		$24	2
	$7	1		$20	2	3	$17	1		$11	1		$25	2
	$18	1		$9	3	4			7				$9	2
	$9	2		$10	3	5			8				$20	3
	$31	2		$17	1	6			9	$14	2	5	$14	3
8				$14	2	7				$19	1		$19	1
9	$14	4		$25	1	8	$14	2	10	$11	1		$20	2
	$15	3		$19	1		$19	1		$12	1		$9	2
	$16	2	4	$11	1		$20	1		$15	1		$10	2
	$19	3		$29	1		$24	1	11	$9	3		$11	3
	$25	1	5				$10	2		$10	3		$29	1
	$20	2	6				$9	3		$14	2	6		
	$10	2	7	$14	2		$7	2		$15	1	7		
	$9	1		$15	2		$11	1		$16	1	8		
10				$19	2	9	$14	2		$19	1	9		
11	$11	3		$25	1		$15	2		$20	1	10		
	$12	1		$16	1		$19	1		$17	2	11		
	$20	2	8			10	$29	1		$25	2	12	$10	6
	$9	1	9				$11	3		$18	2		$9	3
	$10	1	10				$12	1		$24	1		$2	1
	$14	1	11	$18	1		$20	1	12				$1	1
	$17	1	12	$13	1		$9	1	13	$25	1		$14	2
	$29	1	13	$18	1	11	$16	3		$17	1		$19	1
12	$9	5		$31	1		$4	1		$19	1		$17	2
	$20	1		$9	1		$17	3		$2	2		$31	3
	$14	2		$2	1		$14	3		$12	3		$12	3
	$17	1		$10	6		$15	2		$10	2		$25	1
	$18	1		$12	4		$18	1	14	$10	1		$11	1
	$19	1		$31	1		$19	3		$7	1		$18	2
	$23	1		$14	4		$25	3		$9	1	13	$12	1
	$24	2		$19	2		$9	1		$12	1		$10	1
	$25	2		$20	1		$20	2		$23	1		$17	3
	$11	2		$23	1		$23	1	15	$9	1		$14	1
	$10	4		$17	4		$24	2		$19	1	14		
	$7	2		$7	1		$10	1						
13				$16	1	12								
14			14			13								
15			15		3	14								
16						15	$2	1						
18							$18	1						
19							$10	1						
							$9	1						
**total**		**83**			**82**			**85**			**59**			**83**

Clusters in hierarchical trees obtained from different subsets of the transcription profiles or with complete instead of average linkage were examined for the presence of two or more previously identified interactions between GTPases, GEFs and GAPs, as were listed with an alphanumerical ($n_) and colour code in Supplemental_File_S3. The number of interactions per cluster equals the number of identical codes minus 1.

REFERENCES

Abe T, Langenick J, Williams JG. 2003. Rapid generation of gene disruption constructs by in vitro transposition and identification of a Dictyostelium protein kinase that regulates its rate of growth and development. *Nucleic Acids Res* **31**: e107.

Abysalh JC, Kuchnicki LL, Larochelle DA. 2003. The identification of Pats1, a novel gene locus required for cytokinesis in Dictyostelium discoideum. *Molecular Biology of the Cell* **14**: 14-25.

Albert S, Will E, Gallwitz D. 1999. Identification of the catalytic domains and their functionally critical arginine residues of two yeast GTPase-activating proteins specific for Ypt/Rab transport GTPases. *EMBO J* **18**: 5216-5225.

Arigoni M, Bracco E, Lusche DF, Kae H, Weeks G, Bozzaro S. 2005. A novel Dictyostelium RasGEF required for chemotaxis and development. *BMC Cell Biol* **6**.

Bailo N, Cosson P, Charette SJ, Paquet VE, Doublet P, Letourneur F. 2014. Defective lysosome maturation and Legionella pneumophila replication in Dictyostelium cells mutant for the Arf GAP ACAP-A. *J Cell Sci* **127**: 4702-4713.

Becker M, Matzner M, Gerisch G. 1999. Drainin required for membrane fusion of the contractile vacuole in Dictyostelium is the prototype of a protein family also represented in man. *EMBO J* **18**: 3305-3316.

Bloomfield G, Traynor D, Sander SP, Veltman DM, Pachebat JA, Kay RR. 2015. Neurofibromin controls macropinocytosis and phagocytosis in Dictyostelium. *eLife* **4**.

Bolourani P, Spiegelman GB, Weeks G. 2006. Delineation of the roles played by RasG and RasC in cAMP-dependent signal transduction during the early development of Dictyostelium discoideum. *Molecular Biology of the Cell* **17**: 4543-4550.

Bosgraaf L, Waijer A, Engel R, Visser AJ, Wessels D, Soll D, van Haastert PJ. 2005. RasGEF-containing proteins GbpC and GbpD have differential effects on cell polarity and chemotaxis in Dictyostelium. *J Cell Sci* **118**: 1899-1910.

Boudhraa Z, Carmona E, Provencher D, Mes-Masson A-M. 2020. Ran GTPase: A Key Player in Tumor Progression and Metastasis. *Frontiers in Cell and Developmental Biology* **8**.

Buckley CM, Pots H, Gueho A, Vines JH, Munn CJ, Phillips BA, Gilsbach B, Traynor D, Nikolaev A, Soldati T et al. 2020. Coordinated Ras and Rac Activity Shapes Macropinocytic Cups and Enables Phagocytosis of Geometrically Diverse Bacteria. *Curr Biol* **30**: 2912-2926.e2915.

Buczynski G, Bush J, Zhang LY, Rodrigues-Paris J, Cardelli JRA. 1997. Evidence for a recycling role for rab7 in regulating a late step in endocytosis and in retention of lysosomal enzymes in dictyostelium discoideum. *Molecular Biology Of The Cell* **8**: 1343-1360.

Bush J, Temesvari L, Rodriquez-Paris J, Buczynski G, Cardelli J. 1996. A role for a Rab4-like GTPase in endocytosis and in regulation of contractile vacuole structure and function in Dictyostelium discoideum. *MolBiolCell* **7**: 1623-1638.

Casanova JE. 2007. Regulation of Arf activation: the Sec7 family of guanine nucleotide exchange factors. *Traffic* **8**: 1476-1485.

Charest PG, Shen Z, Lakoduk A, Sasaki AT, Briggs SP, Firtel RA. 2010. A Ras signaling complex controls the RasC-TORC2 pathway and directed cell migration. *Dev Cell* **18**: 737-749.

Chattwood A, Nagayama K, Bolourani P, Harkin L, Kamjoo M, Weeks G, Thompson CR. 2013. Developmental lineage priming in Dictyostelium by heterogeneous Ras activation. *eLife* **2**: e01067.

Chen PW, Randazzo PA, Parent CA. 2010. ACAP-A/B are ArfGAP homologs in *Dictyostelium* involved in sporulation but not in chemotaxis. *PLoS One* **5**: e8624.

Dias M, Blanc C, Thazar-Poulot N, Ben Larbi S, Cosson P, Letourneur F. 2013. Dictyostelium ACAP-A is an ArfGAP involved in cytokinesis, cell migration and actin cytoskeleton dynamics. *J Cell Sci* **126**: 756-766.

Dragoi IA, OHalloran TJ. 1998. Cloning and characterization of a dictyostelium gene encoding a small gtpase of the rab11 family. *Journal Of Cellular Biochemistry* **70**: 29-37.

Du F, Edwards K, Shen Z, Sun B, De Lozanne A, Briggs S, Firtel RA. 2008. Regulation of contractile vacuole formation and activity in Dictyostelium. *EMBO J* **27**: 2064-2076.

Essid M, Gopaldass N, Yoshida K, Merrifield C, Soldati T. 2012. Rab8a regulates the exocyst-mediated kiss-and-run discharge of the Dictyostelium contractile vacuole. *Mol Biol Cell* **23**: 1267-1282.

Forbes G, Chen ZH, Kin K, Lawal HM, Schilde C, Yamada Y, Schaap P. 2019. Phylogeny-wide conservation and change in developmental expression, cell-type specificity and functional domains of the transcriptional regulators of social amoebas. *BMC Genomics* **20**: 890.

Fort P, Blangy A. 2017. The Evolutionary Landscape of Dbl-Like RhoGEF Families: Adapting Eukaryotic Cells to Environmental Signals. *Genome biology and evolution* **9**: 1471-1486.

Galili T. 2015. dendextend: an R package for visualizing, adjusting and comparing trees of hierarchical clustering. *Bioinformatics* **31**: 3718-3720.

Garcia-Mata R, Boulter E, Burridge K. 2011. The 'invisible hand': regulation of RHO GTPases by RHOGDIs. *Nature reviews Molecular cell biology* **12**: 493-504.

Geissler H, Ullmann R, Soldati T. 2000. The tail domain of myosin M catalyses nucleotide exchange on Rac1 GTPases and can induce actin-driven surface protrusions. *Traffic* **1**: 399-410.

Gloeckner G, Lawal HM, Felder M, Singh R, Singer G, Weijer CJ, Schaap P. 2016. The multicellularity genes of dictyostelid social amoebas. *Nature communications* **7**: 12085.

Gruenheit N, Parkinson K, Brimson CA, Kuwana S, Johnson EJ, Nagayama K, Llewellyn J, Salvidge WM, Stewart B, Keller T et al. 2018. Cell Cycle Heterogeneity Can Generate Robust Cell Type Proportioning. *Dev Cell* **47**: 494-508.e494.

Hadjebi O, Casas-Terradellas E, Garcia-Gonzalo FR, Rosa JL. 2008. The RCC1 superfamily: from genes, to function, to disease. *Biochim Biophys Acta* **1783**: 1467-1479.

Hadwiger JA. 2013. Role of the Vps9-domain protein RgfA in Dictyostelium chemotaxis and development. *Can J Microbiol* **59**: 22-27.

Harris E, Cardelli J. 2002. RabD, a Dictyostelium Rab14-related GTPase, regulates phagocytosis and homotypic phagosome and lysosome fusion. *Journal of Cell Science* **115**: 3703-3713.

Heath RJ, Insall RH. 2008. Dictyostelium MEGAPs: F-BAR domain proteins that regulate motility and membrane tubulation in contractile vacuoles. *J Cell Sci* **121**: 1054-1064.

Hilbi H, Rothmeier E, Hoffmann C, Harrison CF. 2014. Beyond Rab GTPases Legionella activates the small GTPase Ran to promote microtubule polymerization, pathogen vacuole motility, and infection. *Small GTPases* **5**: e972859.

Homma Y, Hiragi S, Fukuda M. 2020. Rab family of small GTPases: an updated view on their regulation and functions. *The FEBS journal* doi:10.1111/febs.15453.

Inaba H, Yoda K, Adachi H. 2017. The F-actin-binding RapGEF GflB is required for efficient macropinocytosis in Dictyostelium. *J Cell Sci* **130**: 3158-3172.

Ishida M, E. Oguchi M, Fukuda M. 2016. Multiple Types of Guanine Nucleotide Exchange Factors (GEFs) for Rab Small GTPases. *Cell Structure and Function* **41**: 61-79.

Isik N, Brzostowski JA, Jin T. 2008. An Elmo-like protein associated with myosin II restricts spurious F-actin events to coordinate phagocytosis and chemotaxis. *Dev Cell* **15**: 590-602.

Jeon TJ, Lee DJ, Lee S, Weeks G, Firtel RA. 2007. Regulation of Rap1 activity by RapGAP1 controls cell adhesion at the front of chemotaxing cells. *Journal of Cell Biology* **179**: 833-843.

Jeon TJ, Lee S, Weeks G, Firtel RA. 2009. Regulation of Dictyostelium morphogenesis by RapGAP3. *Dev Biol* **328**: 210-220.

Kae H, Kortholt A, Rehmann H, Insall R, Van Haastert PJM, Spiegelman GB, Weeks G. 2007. Cyclic AMP signalling in Dictyostelium: G-proteins activate separate Ras pathways using specific RasGEFs. *EMBO Rep* **8**: 477-482.

Kee YS, Ren Y, Dorfman D, Iijima M, Firtel R, Iglesias PA, Robinson DN. 2012. A mechanosensory system governs myosin II accumulation in dividing cells. *Mol Biol Cell* **23**: 1510-1523.

Khurana T, Brzostowski JA, Kimmel AR. 2005. A Rab21/LIM-only/CH-LIM complex regulates phagocytosis via both activating and inhibitory mechanisms. *Embo Journal* **24**: 2254-2264.

Kibler K, Nguyen TL, Svetz J, Van Driessche N, Ibarra M, Thompson C, Shaw C, Shaulsky G. 2003. A novel developmental mechanism in Dictyostelium revealed in a screen for communication mutants. *Dev Biol* **259**: 193-208.

Kicka S, Shen Z, Annesley SJ, Fisher PR, Lee S, Briggs S, Firtel RA. 2011. The LRRK2-related Roco kinase Roco2 is regulated by Rab1A and controls the actin cytoskeleton. *Mol Biol Cell* **22**: 2198-2211.

Kim J, Kim E. 2016. Rag GTPase in amino acid signaling. *Amino acids* **48**: 915-928.

Kim JJ, Lipatova Z, Segev N. 2016. TRAPP Complexes in Secretion and Autophagy. *Frontiers in Cell and Developmental Biology* **4**.

Kin K, Forbes G, Cassidy A, Schaap P. 2018. Cell-type specific RNA-Seq reveals novel roles and regulatory programs for terminally differentiated Dictyostelium cells. *BMC Genomics* **19**: 764.

Kjellin J, Pränting M, Bach F, Vaid R, Edelbroek B, Li Z, Hoeppner MP, Grabherr M, Isberg RR, Hagedorn M et al. 2019. Investigation of the host transcriptional response to intracellular bacterial infection using Dictyostelium discoideum as a host model. *BMC Genomics* **20**: 961.

Knetsch ML, Schäfers N, Horstmann H, Manstein DJ. 2001. The Dictyostelium Bcr/Abr-related protein DRG regulates both Rac- and Rab-dependent pathways. *Embo j* **20**: 1620-1629.

Kortholt A, Keizer-Gunnink I, Kataria R, Van Haastert PJ. 2013. Ras activation and symmetry breaking during Dictyostelium chemotaxis. *J Cell Sci* **126**: 4502-4513.

Kortholt A, Rehmann H, Kae H, Bosgraaf L, Keizer-Gunnink I, Weeks G, Wittinghofer A, Van Haastert PJM. 2006. Characterization of the GbpD-activated Rap1 pathway regulating adhesion and cell polarity in Dictyostelium discoideum. *Journal of Biological Chemistry* **281**: 23367-23376.

Kuwayama H, Miyanaga Y, Urushihara H, Ueda M. 2013. A RabGAP regulates life-cycle duration via trimeric G-protein cascades in Dictyostelium discoideum. *PLoS One* **8**: e81811.

Lamber EP, Siedenburg AC, Barr FA. 2019. Rab regulation by GEFs and GAPs during membrane traffic. *Curr Opin Cell Biol* **59**: 34-39.

Laurin M, Côté JF. 2014. Insights into the biological functions of Dock family guanine nucleotide exchange factors. *Genes & development* **28**: 533-547.

Lee MR, Jeon TJ. 2012. Cell migration: regulation of cytoskeleton by Rap1 in Dictyostelium discoideum. *Journal of microbiology (Seoul, Korea)* **50**: 555-561.

Lee S, Escalante R, Firtel RA. 1997. A Ras GAP is essential for cytokinesis and spatial patterning in Dictyostelium. *Development* **124**: 983-996.

Lee S, Shen Z, Robinson DN, Briggs S, Firtel RA. 2010. Involvement of the cytoskeleton in controlling leading-edge function during chemotaxis. *Mol Biol Cell* **21**: 1810-1824.

Levine TP, Daniels RD, Wong LH, Gatta AT, Gerondopoulos A, Barr FA. 2013. Discovery of new Longin and Roadblock domains that form platforms for small GTPases in Ragulator and TRAPP-II. *Small GTPases* **4**: 62-69.

Li X, Edwards M, Swaney KF, Singh N, Bhattacharya S, Borleis J, Long Y, Iglesias PA, Chen J, Devreotes PN. 2018. Mutually inhibitory Ras-PI(3,4)P2 feedback loops mediate cell migration. *Proc Natl Acad Sci U S A* **115**: E9125-e9134.

Lim CJ, Spiegelman GB, Weeks G. 2001. RasC is required for optimal activation of adenylyl cyclase and Akt/PKB during aggregation. *Embo J* **20**: 4490-4499.

Liu Y, Lacal J, Veltman Douwe M, Fusetti F, van Haastert Peter JM, Firtel Richard A, Kortholt A. 2016. A Galpha-Stimulated RapGEF Is a Receptor-Proximal Regulator of *Dictyostelium* Chemotaxis. *Dev Cell* **37**: 458-472.

Loovers HM, Postma M, Keizer-Gunnink I, Huang YE, Devreotes PN, van Haastert PJM. 2006. Distinct roles of PI(3,4,5)P-3 during chemoattractant signaling in Dictyostelium: A quantitative in vivo analysis by inhibition of PI3-kinase. *Molecular Biology of the Cell* **17**: 1503-1513.

Loovers HM, Veenstra K, Snippe H, Pesesse X, Erneux C, van Haastert PJ. 2003. A diverse family of inositol 5-phosphatases playing a role in growth and development in Dictyostelium discoideum. *J Biol Chem* **278**: 5652-5658.

Luscher A, Fröhlich F, Barisch C, Littlewood C, Metcalfe J, Leuba F, Palma A, Pirruccello M, Cesareni G, Stagi M et al. 2019. Lowe syndrome-linked endocytic adaptors direct membrane cycling kinetics with OCRL in Dictyostelium discoideum. *Mol Biol Cell* **30**: 2268-2282.

Maringer K, Yarbrough A, Sims-Lucas S, Saheb E, Jawed S, Bush J. 2016. Dictyostelium discoideum RabS and Rab2 colocalize with the Golgi and contractile vacuole system and regulate osmoregulation. *J Biosci* **41**: 205-217.

Marinović M, Mijanović L, Šoštar M, Vizovišek M, Junemann A, Fonović M, Turk B, Weber I, Faix J, Filić V. 2019. IQGAP-related protein IqgC suppresses Ras signaling during large-scale endocytosis. *Proc Natl Acad Sci U S A* **116**: 1289-1298.

Matchett KB, McFarlane S, Hamilton SE, Eltuhamy YSA, Davidson MA, Murray JT, Faheem AM, El-Tanani M. 2014. Ran GTPase in Nuclear Envelope Formation and Cancer Metastasis. In *Cancer Biology and the*

Nuclear Envelope: Recent Advances May Elucidate Past Paradoxes, doi:10.1007/978-1-4899-8032-8_15 (ed. EC Schirmer, JI de las Heras), pp. 323-351. Springer New York, New York, NY.

Minaker SW, Filiatrault MC, Ben-Aroya S, Hieter P, Stirling PC. 2013. Biogenesis of RNA polymerases II and III requires the conserved GPN small GTPases in Saccharomyces cerevisiae. *Genetics* **193**: 853-864.

Mondal S, Bakthavatsalam D, Steimle P, Gassen B, Rivero F, Noegel AA. 2008. Linking Ras to myosin function: RasGEF Q, a Dictyostelium exchange factor for RasB, affects myosin II functions. *J Cell Biol* **181**: 747-760.

Mondal S, Neelamegan D, Rivero F, Noegel AA. 2007. GxcDD, a putative RacGEF, is involved in Dictyostelium development. *BMC Cell Biol* **8**.

Muller R, Herr C, Sukumaran SK, Omosigho NN, Plomann M, Riyahi TY, Stumpf M, Swaminathan K, Tsangarides M, Yiannakou K et al. 2013. The cytohesin paralog Sec7 of Dictyostelium discoideum is required for phagocytosis and cell motility. *Cell Commun Signal* **11**: 54.

Mun H, Lee MR, Jeon TJ. 2014. RapGAP9 regulation of the morphogenesis and development in Dictyostelium. *Biochem Biophys Res Commun* **446**: 428-433.

Muramoto T, Takeda S, Furuya Y, Urushihara H. 2005. Reverse genetic analyses of gamete-enriched genes revealed a novel regulator of the cAMP signaling pathway in Dictyostelium discoideum. *Mech Dev* **122**: 733-743.

Nichols JME, Paschke P, Peak-Chew S, Williams TD, Tweedy L, Skehel M, Stephens E, Chubb JR, Kay RR. 2019. The Atypical MAP Kinase ErkB Transmits Distinct Chemotactic Signals through a Core Signaling Module. *Dev Cell* **48**: 491-505.e499.

Pakes NK, Veltman DM, Williams RS. 2013. Zizimin and Dock guanine nucleotide exchange factors in cell function and disease. *Small GTPases* **4**: 22-27.

Para A, Krischke M, Merlot S, Shen Z, Oberholzer M, Lee S, Briggs S, Firtel RA. 2009. Dictyostelium Dock180-related RacGEFs regulate the actin cytoskeleton during cell motility. *Mol Biol Cell* **20**: 699-707.

Parikh A, Miranda ER, Katoh-Kurasawa M, Fuller D, Rot G, Zagar L, Curk T, Sucgang R, Chen R, Zupan B et al. 2010. Conserved developmental transcriptomes in evolutionarily divergent species. *Genome Biol* **11**: R35.

Park B, Kim H, Jeon TJ. 2018. Loss of RapC causes defects in cytokinesis, cell migration, and multicellular development of Dictyostelium. *Biochem Biophys Res Commun* **499**: 783-789.

Park KC, Rivero F, Meili R, Lee S, Apone F, Firtel RA. 2004. Rac regulation of chemotaxis and morphogenesis in Dictyostelium. *Embo J* **23**: 4177-4189.

Parkinson K, Baines AE, Keller T, Gruenheit N, Bragg L, North RA, Thompson CR. 2014. Calcium-dependent regulation of Rab activation and vesicle fusion by an intracellular P2X ion channel. *Nat Cell Biol* **16**: 87-98.

Parkinson K, Bolourani P, Traynor D, Aldren NL, Kay RR, Weeks G, Thompson CR. 2009. Regulation of Rap1 activity is required for differential adhesion, cell-type patterning and morphogenesis in Dictyostelium. *J Cell Sci* **122**: 335-344.

Phillips JE, Gomer RH. 2012. A secreted protein is an endogenous chemorepellant in Dictyostelium discoideum. *Proc Natl Acad Sci U S A* **109**: 10990-10995.

Plak K, Keizer-Gunnink I, van Haastert PJ, Kortholt A. 2014. Rap1-dependent pathways coordinate cytokinesis in Dictyostelium. *Mol Biol Cell* **25**: 4195-4204.

Plak K, Veltman D, Fusetti F, Beeksma J, Rivero F, Van Haastert PJ, Kortholt A. 2013. GxcC connects Rap and Rac signaling during Dictyostelium development. *BMC Cell Biol* **14**: 6.

Renault L, Nassar N, Vetter I, Becker J, Klebe C, Roth M, Wittinghofer A. 1998. The 1.7 A crystal structure of the regulator of chromosome condensation (RCC1) reveals a seven-bladed propeller. *Nature* **392**: 97-101.

Rivero F, Dislich H, Glockner G, Noegel AA. 2001. The Dictyostelium discoideum family of Rho-related proteins. *Nucleic Acids Res* **29**: 1068-1079.

Rivero F, Illenberger D, Somesh BP, Dislich H, Adam N, Meyer AK. 2002. Defects in cytokinesis, actin reorganization and the contractile vacuole in cells deficient in RhoGDI. *Embo Journal* **21**: 4539-4549.

Rivero F, Xiong H. 2016. Rho Signaling in Dictyostelium discoideum. *Int Rev Cell Mol Biol* **322**: 61-181.

Ronquist F, Huelsenbeck JP. 2003. MrBayes 3: Bayesian phylogenetic inference under mixed models. *Bioinformatics* **19**: 1572-1574.

Rosel D, Khurana T, Majithia A, Huang X, Bhandari R, Kimmel AR. 2012. TOR complex 2 (TORC2) in Dictyostelium suppresses phagocytic nutrient capture independently of TORC1-mediated nutrient sensing. *J Cell Sci* **125**: 37-48.

Rupper A, Lee K, Knecht D, Cardelli J. 2001. Sequential activities of phosphoinositide 3-kinase, pkb/akt, and rab7 during macropinosome formation in dictyostelium. *Mol Biol Cell* **12**: 2813-2824.

Santorelli LA, Thompson CRL, Villegas E, Svetz J, Dinh C, Parikh A, Sucgang R, Kuspa A, Strassmann JE, Queller DC et al. 2008. Facultative cheater mutants reveal the genetic complexity of cooperation in social amoebae. *Nature* **451**: 1107-1110.

Sawai S, Guan XJ, Kuspa A, Cox EC. 2008. High-throughput analysis of spatio-temporal dynamics in *Dictyostelium*. *Genome Biol* **8**: R144 (115 pages).

Schultz J, Milpetz F, Bork P, Ponting CP. 1998. SMART, a simple modular architecture research tool: identification of signaling domains. *Proc Natl Acad Sci USA* **95**: 5857-5864.

Secko DM, Insall RH, Spiegelman GB, Weeks G. 2004. The identification of Dictyostelium phosphoproteins altered in response to the activation of RasG. *Proteomics* **4**: 2629-2639.

Seewald MJ, Körner C, Wittinghofer A, Vetter IR. 2002. RanGAP mediates GTP hydrolysis without an arginine finger. *Nature* **415**: 662-666.

Senoo H, Cai H, Wang Y, Sesaki H, Iijima M. 2016. The novel RacE-binding protein GflB sharpens Ras activity at the leading edge of migrating cells. *Mol Biol Cell* **27**: 1596-1605.

Shannon P, Markiel A, Ozier O, Baliga NS, Wang JT, Ramage D, Amin N, Schwikowski B, Ideker T. 2003. Cytoscape: a software environment for integrated models of biomolecular interaction networks. *Genome Res* **13**: 2498-2504.

Shimodaira H, Terada Y. 2019. Selective Inference for Testing Trees and Edges in Phylogenetics. *Frontiers in Ecology and Evolution* **7**.

Shina MC, Muller R, Blau-Wasser R, Glockner G, Schleicher M, Eichinger L, Noegel AA, Kolanus W. 2010. A cytohesin homolog in Dictyostelium amoebae. *PLoS One* **5**: e9378.

Simanshu DK, Nissley DV, McCormick F. 2017. RAS Proteins and Their Regulators in Human Disease. *Cell* **170**: 17-33.

Stamatakis A. 2014. RAxML Version 8: A tool for Phylogenetic Analysis and Post-Analysis of Large Phylogenies. *Bioinformatics* doi:10.1093/bioinformatics/btu033.

Staresincic L, Walker J, Dirac-Svejstrup AB, Mitter R, Svejstrup JQ. 2011. GTP-dependent binding and nuclear transport of RNA polymerase II by Npa3 protein. *The Journal of biological chemistry* **286**: 35553-35561.

Strehle A, Schleicher M, Faix J. 2006. Trix, a novel Rac guanine-nucleotide exchange factor from Dictyostelium discoideum is an actin-binding protein and accumulates at endosomes. *Eur J Cell Biol* **85**: 1035-1045.

Suzuki R, Shimodaira H. 2006. Pvclust: an R package for assessing the uncertainty in hierarchical clustering. *Bioinformatics* **22**: 1540-1542.

Swart AL, Steiner B, Gomez-Valero L, Schütz S, Hannemann M, Janning P, Irminger M, Rothmeier E, Buchrieser C, Itzen A et al. 2020. Divergent Evolution of Legionella RCC1 Repeat Effectors Defines the Range of Ran GTPase Cycle Targets. *mBio* **11**.

Sztul E, Chen PW, Casanova JE, Cherfils J, Dacks JB, Lambright DG, Lee FS, Randazzo PA, Santy LC, Schürmann A et al. 2019. ARF GTPases and their GEFs and GAPs: concepts and challenges. *Mol Biol Cell* **30**: 1249-1271.

Tang M, Iijima M, Kamimura Y, Chen L, Long Y, Devreotes P. 2011. Disruption of PKB signaling restores polarity to cells lacking tumor suppressor PTEN. *Mol Biol Cell* **22**: 437-447.

Tanna CE, Goss LB, Ludwig CG, Chen PW. 2019. Arf GAPs as Regulators of the Actin Cytoskeleton-An Update. *International journal of molecular sciences* **20**.

Thomas LL, Fromme JC. 2020. Extensive GTPase crosstalk regulates Golgi trafficking and maturation. *Curr Opin Cell Biol* **65**: 1-7.

Torija P, Vicente JJ, Rodrigues TB, Robles A, Cerdan S, Sastre L, Calvo RM, Escalante R. 2006. Functional genomics in Dictyostelium: MidA, a new conserved protein, is required for mitochondrial function and development. *Journal of Cell Science* **119**: 1154-1164.

Ueyama T. 2019. Rho-Family Small GTPases: From Highly Polarized Sensory Neurons to Cancer Cells. *Cells* **8**.

van Egmond WN, Kortholt A, Plak K, Bosgraaf L, Bosgraaf S, Keizer-Gunnink I, van Haastert PJ. 2008. Intramolecular activation mechanism of the Dictyostelium LRRK2 homolog Roco protein GbpC. *J Biol Chem* **283**: 30412-30420.

van Egmond WN, van Haastert PJ. 2010. Characterization of the Roco protein family in Dictyostelium discoideum. *Eukaryot Cell* **9**: 751-761.

Vlahou G, Elias M, von Kleist-Retzow JC, Wiesner RJ, Rivero F. 2011. The Ras related GTPase Miro is not required for mitochondrial transport in Dictyostelium discoideum. *Eur J Cell Biol* **90**: 342-355.

Wang Y, Senoo H, Sesaki H, Iijima M. 2013. Rho GTPases orient directional sensing in chemotaxis. *Proc Natl Acad Sci U S A* **110**: E4723-4732.

Wauters L, Versées W, Kortholt A. 2019. Roco Proteins: GTPases with a Baroque Structure and Mechanism. *International journal of molecular sciences* **20**.

Weber SS, Ragaz C, Hilbi H. 2009. The inositol polyphosphate 5-phosphatase OCRL1 restricts intracellular growth of Legionella, localizes to the replicative vacuole and binds to the bacterial effector LpnE. *Cell Microbiol* **11**: 442-460.

Wilkins A, Chubb JR, Insall RH. 2000a. A novel Dictyostelium RasGEF is required for normal endocytosis, cell motility and multicellular development. *Curr Biol* **10**: 1427-1437.

Wilkins A, Khosla M, Fraser DJ, Spiegelman GB, Fisher PR, Weeks G, Insall RH. 2000b. Dictyostelium RasD is required for normal phototaxis, but not differentiation. *Genes Dev* **14**: 1407-1413.

Wilkins A, Szafranski K, Fraser DJ, Bakthavatsalam D, Muller R, Fisher PR, Glockner G, Eichinger L, Noegel AA, Insall RH. 2005. The Dictyostelium genome encodes numerous RasGEFs with multiple biological roles. *Genome Biol* **6**.

Williams TD, Paschke PI, Kay RR. 2019a. Function of small GTPases in Dictyostelium macropinocytosis. *Philos Trans R Soc Lond B Biol Sci* **374**: 20180150.

Williams TD, Peak-Chew SY, Paschke P, Kay RR. 2019b. Akt and SGK protein kinases are required for efficient feeding by macropinocytosis. *J Cell Sci* **132**.

Xu X, Jin T. 2019. ELMO proteins transduce G protein-coupled receptor signal to control reorganization of actin cytoskeleton in chemotaxis of eukaryotic cells. *Small GTPases* **10**: 271-279.

Xu X, Wen X, Veltman DM, Keizer-Gunnink I, Pots H, Kortholt A, Jin T. 2017. GPCR-controlled membrane recruitment of negative regulator C2GAP1 locally inhibits Ras signaling for adaptation and long-range chemotaxis. *Proc Natl Acad Sci U S A* **114**: E10092-e10101.

Yan J, Mihaylov V, Xu X, Brzostowski JA, Li H, Liu L, Veenstra TD, Parent CA, Jin T. 2012. A Gβγ effector, ElmoE, transduces GPCR signaling to the actin network during chemotaxis. *Dev Cell* **22**: 92-103.

Zhang S, Charest PG, Firtel RA. 2008. Spatiotemporal regulation of Ras activity provides directional sensing. *Curr Biol* **18**: 1587-1593.

Zhang X, Zhuchenko O, Kuspa A, Soldati T. 2016. Social amoebae trap and kill bacteria by casting DNA nets. *Nature communications* **7**: 10938.
