# Supplementary figures and images for: Interactome and evolutionary conservation of Dictyostelid small GTPases and their direct regulators
Source: Small GTPases. 2021 Oct 5;13(1):239–54. doi: 10.1080/21541248.2021.1984829 (PMC8923023; doi:10.1080/21541248.2021.1984829)

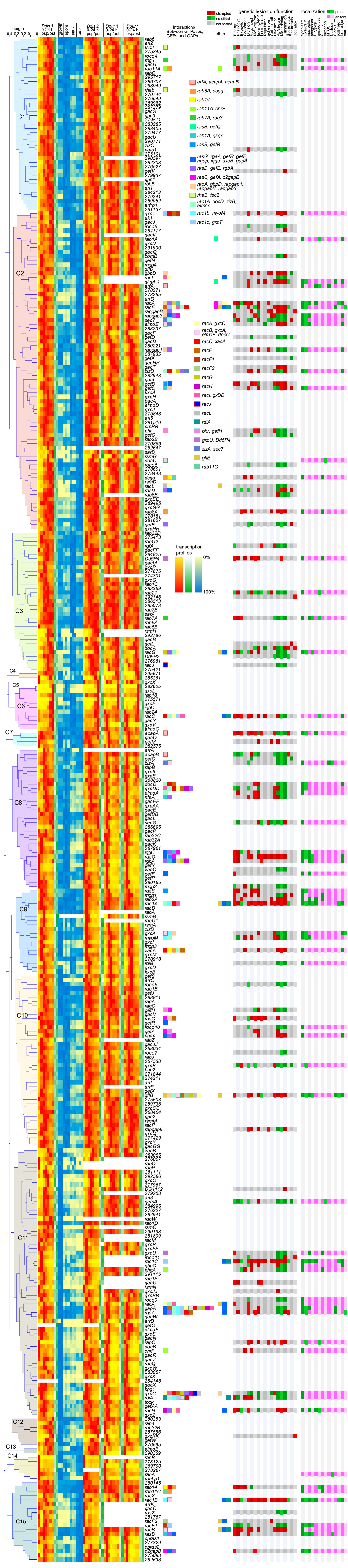

Supplement: Supplemental Material [file KSGT_A_1984829_SM5747.zip › supplementary/Supplemental_Fig_S17.pdf]

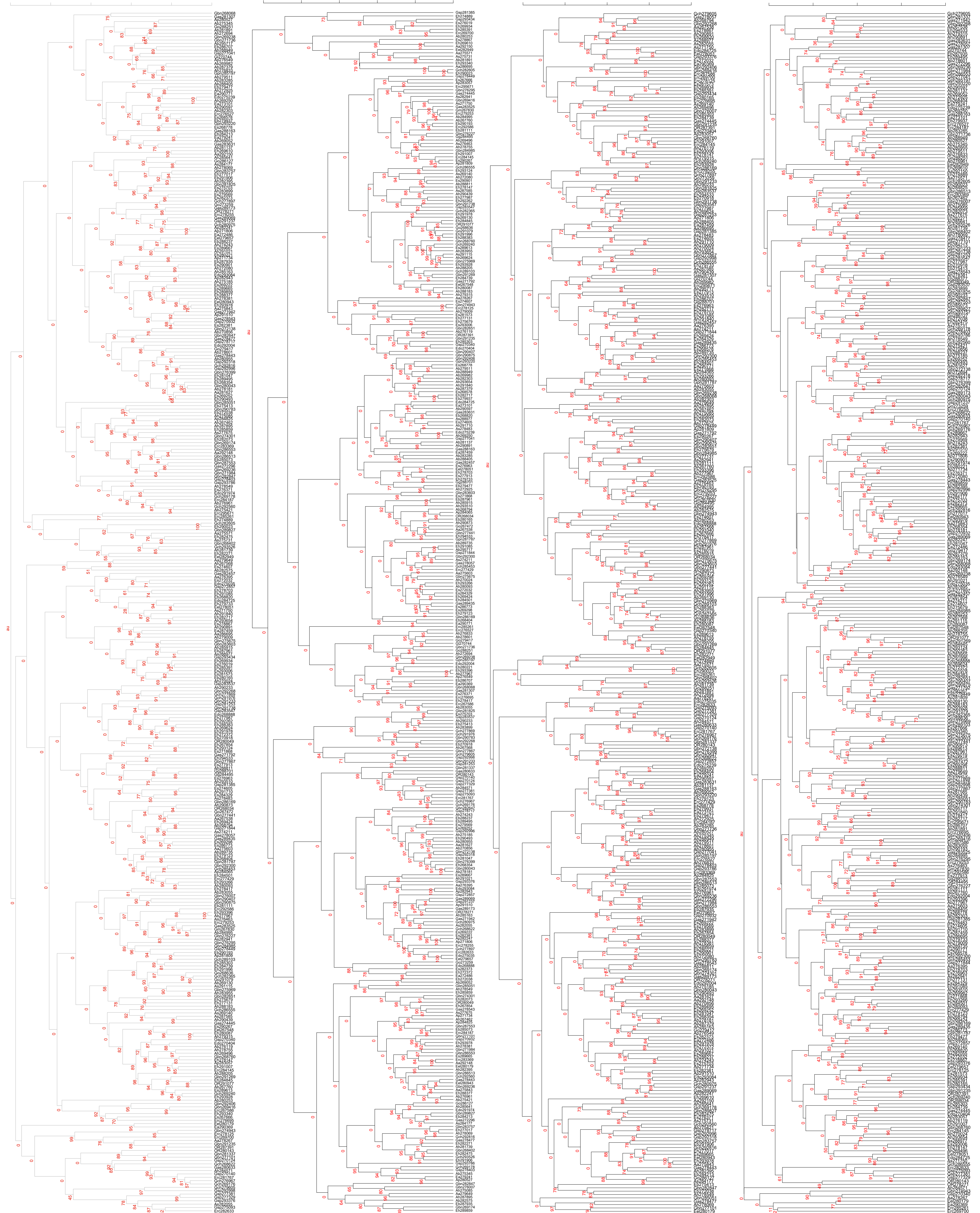

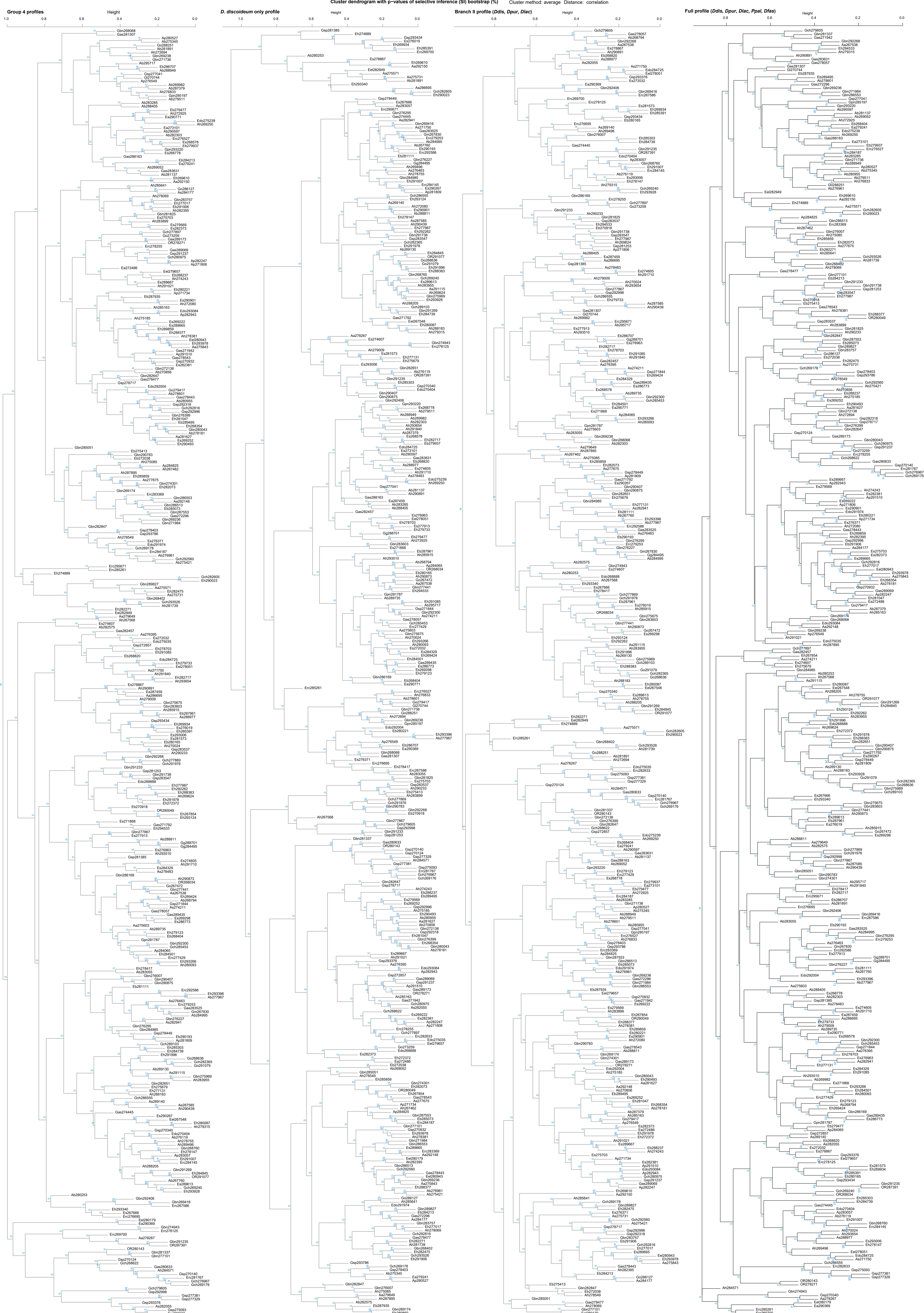

Supplement: Supplemental Material [file KSGT_A_1984829_SM5747.zip › supplementary/Supplemental_Fig_S20.pdf]
